# Supplementary material for: Identification of Small-Molecule Bioactive Constituents from the Leaves of Vaccinium bracteatum Confirms It as a Potential Functional Food with Health Benefits
Source: Foods. 2023 Jan 1;12(1):177. doi: 10.3390/foods12010177 (PMC9818789; doi:10.3390/foods12010177)

***Supplementary materials* for**

Identification of small-molecule bioactive constituents from the leaves of *Vaccinium bracteatum* confirms it as a potential functional food with health benefits

Yin-Yin Wang[a,b], Jun-Sheng Zhang[b], Xin-Xin Wang[b], Lin-Lin Tian[b], Yu-Peng Li[b], Chao Wang[b], Ren-Fen Ma[a,b], Yi-Ke Yin[b], Jie Bao[b], Hua Zhang[b,]*

[a] *School of Chemistry and Chemical Engineering, University of Jinan, Jinan 250022, China* [b] *School of Biological Science and Technology, University of Jinan, Jinan 250022, China*

* Corresponding author at: School of Biological Science and Technology, University of Jinan, Jinan 250022, China.

Email address: bio_zhangh@ujn.edu.cn (H. Zhang)

List of Contents

Table S1. ^{1}H and ^{13}C NMR data of compounds **1–4**…………………………………...6

Table S2. ^{1}H and ^{13}C NMR data of compounds **5–8**…………………………………...7

Table S3. Specific optical rotations and ECD Cotton effects for **9–18**……………........8

Table S4. Structural classes of all the isolated compounds……………………….......9

Materials and Methods

Instrumentation and Reagents……………………………………………….……….10

ECD calculation………………………………………………………………..…….10

Figure S1. $\Delta\delta$ (δ_S–δ_R) values in ppm of MTPA esters for **5** and **8**……………………11

Figure S2. Conformations of the Mo_2^{4+} complexes for **5r** and **8r**, and their respective ECD spectrum……………………………………………………………………...…11

Figure S3. The ^{1}H NMR spectrum of **1** (CD_3OD)…………………………………12

Figure S4. The ^{13}C and DEPT NMR spectra of **1** (CD_3OD) ...………………………12

Figure S5. The ^{1}H-^{1}H COSY spectrum of **1** (CD_3OD)……………….……………..13

Figure S6. The HSQC spectrum of **1** (CD_3OD)…………………………….……..13

Figure S7. The HMBC spectrum of **1** (CD_3OD)……………………………………14

Figure S8. The NOESY spectrum of **1** (CD_3OD)……………………………………14

Figure S9. The (+)-HR-ESIMS spectrum of **1**.………………………………………15

Figure S10. The ^{1}H NMR spectrum of **2** (CD_3OD)……………………………..16

Figure S11. The ^{13}C and DEPT NMR spectra of **2** (CD_3OD)…………………….....16

Figure S12. The ^{1}H-^{1}H COSY spectrum of **2** (CD_3OD)………………………......17

Figure S13. The HSQC spectrum of **2** in (CD_3OD) …………………………………17

Figure S14. The HMBC spectrum of **2** (CD_3OD)……………………………………18

Figure S15. The NOESY spectrum of **2** (CD_3OD)…………………………………..18

Figure S16. The (+)-HR-ESIMS spectrum of **2**.…………………………………...19

Figure S17. The ^{1}H NMR spectrum of **3** (CD_3OD)..………………………………20

Figure S18. The ^{13}C and DEPT NMR spectra of **3** (CD_3OD)..……………………..20

Figure S19. The ^{1}H-^{1}H COSY spectrum of **3** (CD_3OD)...……………………………21

Figure S20. The HSQC spectrum of **3** (CD_3OD)……………………………………..21

Figure S21. The HMBC spectrum of **3** (CD_3OD)…….………………………………22

Figure S22. The NOESY spectrum of **3** (CD_3OD)…..………………………………..22

Figure S23. The (+)-HR-ESIMS spectrum of **3**…… ………………………………..23

Figure S24. The ^{1}H NMR spectrum of **4** (CD_3OD)..…………………………………24

Figure S25. The ^{13}C and DEPT NMR spectra of **4** (CD_3OD)…..……………………..24

Figure S26. The ^{1}H-^{1}H COSY spectrum of **4** (CD_3OD)……………………………..25

Figure S27. The HSQC spectrum of **4** (CD_3OD)..……………………………………25

Figure S28. The HMBC spectrum of **4** (CD_3OD)…….………………………………26

Figure S29. The NOESY spectrum of **4** (CD_3OD)…………………………………26

Figure S30. The (+)-HR-ESIMS spectrum of **4**………………………………………27

Figure S31. The ^{1}H NMR spectrum of **5** (CD_3OD)…...………………………………28

Figure S32. The ^{13}C and DEPT NMR spectra of **5** (CD_3OD)…………………………28

Figure S33. The ^{1}H-^{1}H COSY spectrum of **5** (CD_3OD). ……………………………..29

Figure S34. The HSQC spectrum of **5** (CD_3OD)……..………………………………29

Figure S35. The HMBC spectrum of **5** (CD_3OD)…….………………………………30

Figure S36. The NOESY spectrum of **5** (CD_3OD)…...………………………………30

Figure S37. ^{1}H NMR spectra of (*S*) and (*R*)-MTPA esters of **5** (CD_3OD) ……………31

Figure S38. The (+)-HR-ESIMS spectrum of **5**………………………………………31

Figure S39. The ^{1}H NMR spectrum of **5r** ($CDCl_3$)…...………………………………32

Figure S40. The ^{13}C NMR spectrum of **5r** ($CDCl_3$)…..………………………………32

Figure S41. The ^{1}H-^{1}H COSY spectrum of **5r** ($CDCl_3$)...……………………………33

Figure S42. The HSQC spectrum of **5r** ($CDCl_3$)……..………………………………33

Figure S43. The HMBC spectrum of **5r** ($CDCl_3$)…….………………………………34

Figure S44. The NOESY spectrum of **5r** ($CDCl_3$)……………………………………34

Figure S45. The 1D-NOE spectrum of **5r** ($CDCl_3$, H-4b)……………………………35

Figure S46. The ^{1}H NMR spectrum of **5r** (CD_3OD)….………………………………35

Figure S47 The ^{13}C and DEPT NMR spectra of **5r** (CD_3OD)………………………..36

Figure S48. The ^{1}H NMR spectrum of **6** (CD_3OD)…...………………………………37

Figure S49. The ^{13}C and DEPT NMR spectra of **6** (CD_3OD)..………………………..37

Figure S50. The ^{1}H-^{1}H COSY spectrum of **6** (CD_3OD).……………………………..38

Figure S51. The HSQC spectrum of **6** (CD_3OD)……..………………………………38

Figure S52. The HMBC spectrum of **6** (CD_3OD)…….………………………………39

Figure S53. The NOESY spectrum of **6** (CD_3OD)…...………………………………39

Figure S54. The (+)-HR-ESIMS spectrum of **6**………………………………………40

Figure S55. The ^{1}H NMR spectrum of **7** (CD_3OD)…...………………………………41

Figure S56. The ^{13}C and DEPT NMR spectra of **7** (CD_3OD)..………………………..41

Figure S57. The ^{1}H-^{1}H COSY spectrum of **7** (CD_3OD)...……………………………42

Figure S58. The HSQC spectrum of **7** (CD_3OD)……..………………………………42

Figure S59. The HMBC spectrum of **7** (CD_3OD)…….………………………………43

Figure S60. The NOESY spectrum of **7** (CD_3OD)…...………………………………43

Figure S61. The ^{1}H NMR spectrum of **7** ($CDCl_3$)…….……………………………44

Figure S62. The ^{13}C and DEPT NMR spectra of **7** ($CDCl_3$)..…………………………44

Figure S63. The (+)-HR-ESIMS spectrum of **7**………………………………………45

Figure S64. The ^{1}H NMR spectrum of **8** (CD_3OD)…...………………………………46

Figure S65. The ^{13}C and DEPT NMR spectra of **8** (CD_3OD)..………………………..46

Figure S66. The ^{1}H-^{1}H COSY spectrum of **8** (CD_3OD)...……………………………47

Figure S67. The HSQC spectrum of **8** (CD_3OD)……..………………………………47

Figure S68. The HMBC spectrum of **8** (CD_3OD)…….………………………………48

Figure S69. The NOESY spectrum of **8** (CD_3OD)…...………………………………48

Figure S70. The ^{1}H NMR spectrum of **8** (DMSO-d_6)………………………………49

Figure S71. The ^{13}C and DEPT NMR spectra of **8** (DMSO-d_6) ……………………...49

Figure S72. ^{1}H NMR spectra of (*S*) and (*R*)-MTPA esters of **8** (CD_3OD)……………50

Figure S73. The (+)-HR-ESIMS spectrum of **8**………………………………………50

Figure S74. The ^{1}H NMR spectrum of **8r** ($CDCl_3$)…...………………………………51

Figure S75. The ^{13}C and DEPT NMR spectra of **8r** ($CDCl_3$)…………………………51

Figure S76. The ^{1}H-^{1}H COSY spectrum of **8r** ($CDCl_3$)………………………………52

Figure S77. The NOESY spectrum of **8r** ($CDCl_3$)……………………………………52

Figure S78. The ^{1}H NMR spectrum of **8r** (CD_3OD)….…………………………………53

Figure S79. The ^{13}C and DEPT NMR spectra of **8r** (CD_3OD)..……………………….53

Figure S80. The ^1H NMR spectrum of **9** (CD_3OD)…...……………………………..54

Figure S81. The ^{13}C and DEPT NMR spectra of **9** (CD_3OD)………………………….54

Figure S82. The ^1H-^1H COSY spectrum of **9** (CD_3OD)...……………………………55

Figure S83. The HSQC spectrum of **9** (CD_3OD)……..………………………………55

Figure S84. The HMBC spectrum of **9** (CD_3OD)……..…………………………….…56

Figure S85. The NOESY spectrum of **9** (CD_3OD)…...………………………………56

Figure S86. The (+)-HR-ESIMS spectrum of **9**………………………………………57

Figure S87. The ^1H NMR spectrum of **21** (CD_3OD)…..………………………………58

Figure S88. The ^{13}C and DEPT NMR spectra of **21** (CD_3OD)..……………………….58

Figure S89. The ^1H-^1H COSY spectrum of **21** (CD_3OD).……………………………59

Figure S90. The HSQC spectrum of **21** (CD_3OD)……………………………………59

Figure S91. The HMBC spectrum of **21** (CD_3OD)…...………………………………60

Figure S92. The NOESY spectrum of **21** (CD_3OD)…..………………………………60

Table S1. ^{1}H and ^{13}C NMR data of compounds **1–4** in CD_3OD.

	**1**		**2**		**3**		**4**	
	δ_C	δ_H, multi. (J in Hz)	δ_C	δ_H, multi. (J in Hz)	δ_C	δ_H, multi. (J in Hz)	δ_C	δ_H, multi. (J in Hz)
1	98.0	5.16, d (3.1)	98.0	5.06, d (3.1)	90.5	5.58, d (2.6)	90.5	5.53, d (3.0)
3	92.1	4.96, dd (3.1, 1.2)	92.1	4.93, dd (3.1, 1.2)	92.2	4.95, dd (3.0, 1.2)	92.2	4.93, dd (3.1, 1.2)
4	34.9	a 2.25, ddd (13.9, 10.5, 1.2)	34.9	a 2.22, ddd (14.0, 10.5, 1.2)	34.8	a 2.32[a], m	34.8	a 2.31[c], ddd (13.9, 10.5, 1.2)
		b 1.57, dd (13.9, 3.1)		b 1.55, dd (14.0, 3.1)		b 1.57, dd (14.1, 3.2)		b 1.55, dd (13.9, 3.1)
5	39.9	2.45, brdd (10.5, 4.8)	39.9	2.42, brdd (10.5, 4.8)	39.4	2.53[b], m	39.4	2.51, brdd (10.5, 4.8)
6	77.3	4.05, dd (6.9, 2.0)	77.3	4.02, dd (6.9, 2.1)	77.3	4.06, dd (6.9, 1.9)	77.3	4.03, dd (6.9, 1.9)
7	46.8	a 2.31, dd (14.9, 6.9)	46.9	a 2.26, dd (14.9, 6.9)	47.0	a 2.32[a], dd (15.0, 6.9)	46.9	a 2.28[c], dd (15.0, 6.9)
		b 1.82, ddd (14.9, 2.0, 1.8)		b 1.75, ddd (14.9, 2.1, 1.7)		b 1.84, ddd (15.0, 1.9, 1.6)		b 1.79, ddd (15.0, 1.9, 1.8)
8	82.2		82.1		82.5		82.2	
9	40.4	2.56, dd (4.8, 3.1)	40.5	2.48, dd (4.8, 3.1)	41.7	2.52[b], dd (4.9, 2.6)	41.6	2.47, dd (4.8, 3.0)
10	66.3	a 4.34, d (11.5)	66.3	a 4.30, d (11.6)	66.4	a 4.35, d (11.5)	66.3	a 4.32, d (11.6)
		b 4.14, d (11.5)		b 4.10, d (11.6)		b 4.15, d (11.5)		b 4.10, d (11.6)
1-OMe	55.3	3.42, s	55.3	3.39				
1′	127.1		127.6		127.1		127.6	
2′(6′)	131.3	7.47, d (8.6)	133.7	7.64, d (8.7)	131.3	7.48, d (8.7)	133.8	7.67, d (8.7)
3′(5′)	116.8	6.81, d (8.6)	115.9	6.76, d (8.7)	116.8	6.81, d (8.7)	115.9	6.76, d (8.7)
4′	161.3		160.2		161.4		160.2	
7′	147.1	7.66, d (15.9)	145.5	6.90, d (12.8)	147.1	7.66, d (15.9)	145.8	6.90, d (12.8)
8′	114.7	6.36, d (15.9)	116.1	5.82, d (12.8)	114.7	6.37, d (15.9)	116.0	5.81, d (12.8)
9′	168.7		167.8		168.7		167.7	

[a–c] Data with the same superscript indicate overlapping signals

Table S2. ^{1}H and ^{13}C NMR data of compounds **5–8** in CD_3OD and **5r** and **8r** in $CDCl_3$.

	**5**		**6**		**7**		**8**		**5r**		**8r**	
No.	δ_C	δ_H, multi. (J in Hz)	δ_C	δ_H, multi. (J in Hz)	δ_C	δ_H, multi. (J in Hz)	δ_C	δ_H, multi. (J in Hz)	δ_C	δ_H, multi. (J in Hz)	δ_C	δ_H, multi. (J in Hz)
1	99.1	5.01, d (5.5)	99.1	4.96, d (5.4)	99.0	5.07, d (6.9)	100.4	4.85*	98.2	4.95, d (4.8)	99.2	4.72, d (5.9)
3	99.7	4.77, dd (8.6, 4.6)	99.6	4.75, dd (8.6, 4.6)	100.1	4.82, dd (8.8, 5.2)	99.4	4.78, dd (6.0, 4.0)	97.9	4.80, dd (8.0, 4.1)	98.5	4.74, dd (6.2, 3.8)
4	32.8	a 2.04, ddd (13.3, 5.3, 4.6)	32.8	a 2.03, ddd (13.2, 5.4, 4.6)	34.7	a 2.10, dt (13.6, 5.2)	30.0	a 1.87, m	30.9	a 2.00, ddd (13.8, 5.9, 4.1)	29.1	1.88, m
		b 1.56, ddd (13.3, 12.2, 8.6)		b 1.54, ddd (13.2, 11.8, 8.6)		b 1.39, td (13.6, 8.8)		b 1.84, m		b 1.48, ddd (13.8, 11.1, 8.0)		1.85, ddd (13.8, 6.1, 3.8)
5	44.3	2.18[a], m	44.2	2.14[b], m	42.4	2.83, m	42.0	2.25[c], m	43.6	2.27, dddd (11.1, 8.5, 5.9, 3.8)	41.4	2.24, m
6	77.3	4.14, ddd (6.8, 6.1, 4.6)	77.3	4.11, ddd (7.0, 6.3, 3.8)	138.9	6.00, dd (5.7, 2.6)	75.3	4.14, m	75.8	4.22, ddd (6.2, 4.9, 3.8)	74.5	4.15, q like (7.4)
7	46.7	2.18[a], dd (13.7, 6.1)	46.6	2.12[b], dd (13.7, 6.3)	134.8	5.81, dd (5.7, 1.5)	46.2	2.10, dd (13.5, 6.5)	46.1	a 2.16, dd (14.4, 6.2)	45.1	2.09, dd (13.7, 6.8)
		1.83, dd (13.7, 6.8)		1.77, dd (13.7, 7.0)				1.76, dd (13.5, 8.0)		b 1.79, dd (14.4, 4.9)		1.65, dd (13.7, 7.8)
8	80.0		79.9		83.8		79.0		80.3		79.4	
9	48.3	2.22, dd (9.8, 5.5)	48.2	2.16[b], dd (9.8, 5.7)	48.4	2.21, dd (8.7, 6.9)	47.3	2.25[c], m	46.7	2.23, dd (8.5, 4.8)	46.3	2.19, dd (9.4, 6.0)
10	71.1	4.22, d (11.1)	70.9	4.16, d (11.1)	70.2	4.35, d (11.0)	71.5	4.20, d (11.1)	69.9	a 3.56, d (10.9)	70.1	3.51, d (11.1)
		4.09, d (11.1)		4.05, d (11.1)		4.03, d (11.0)		4.10, d (11.1)		b 3.54, d (10.9)		3.49, d (11.1)
1-OMe	56.1	3.464[d], s	56.1	3.44, s	55.7	3.47, s	56.1	3.454[e], s	55.7	3.49, s	55.89[f]	3.47, s
3-OMe	55.8	3.462[d], s	55.8	3.45, s	56.5	3.49, s	55.9	3.450[e] s	55.9	3.48, s	55.87[f]	3.49, s
1′	127.2		127.6		127.1		127.1			8-OH, 3.16 (s)		
2′(6′)	131.2	7.48, d (8.6)	133.7	7.66, d (8.7)	131.2	7.46, d (8.6)	131.2	7.47, d (8.7)		10-OH, 2.72 (brs)		
3′(5)′	116.8	6.81, d (8.6)	115.9	6.76, d (8.7)	116.8	6.81, d (8.6)	116.8	6.81, d (8.7)		6-OH, 2.04 (brs)		
4′	161.3		160.1		161.3		161.3					
7′	146.8	7.67, d (15.9)	145.3	6.88, d (12.8)	146.8	7.64, d (15.9)	146.9	7.67, d (16.0)				
8′	115.0	6.37, d (15.9)	116.3	5.82, d (12.8)	115.0	6.34, d (15.9)	114.9	6.36, d (16.0)				
9′	169.1		168.1		169.0		169.0					

[a–c] Data with the same superscript indicate overlapping signals; [d–f] Assignments with the same superscript may be interchangeable; * Overlapped by solvent peak.

Table S3. Specific optical rotations and ECD Cotton effects for **9–18**.

No.	$[\alpha]_D^{25}$ (g/100 mL)[a]	ECD (mg/mL)[b]: λ (Δε) nm
**9a**	+23.0 (0.10)	(0.12): 203 (3.99), 214 (–0.73), 228 (–0.63), 243 (1.56), 281 (0.68)
**9b**	–21.0 (0.10)	(0.11): 203 (–3.38), 214 (0.23), 228 (0.49), 243 (–1.23), 281(–0.42)
**10a**	–30.1 (0.05)	(0.025): 199 (–4.19), 210 (0.90), 226 (1.64), 239 (–4.16), 282 (–1.05)
**10b**	+40.1 (0.05)	(0.025): 199 (5.99), 210 (–2.34), 226 (–2.74), 239 (4.97), 282 (1.05)
**11a**	–75.1 (0.10)	(0.02): 207 (1.36), 236 (–1.78)
**11b**	+58.1 (0.10)	(0.01): 207 (–0.71), 236 (1.00)
**12a**	+14.0 (0.10)	(0.013): 202 (–7.69), 211 (9.69), 227 (–1.54) , 241 (1.46), 293 (1.62)
**12b**	–13.0 (0.10)	(0.013): 202 (7.53), 211 (–10.02), 227 (1.87), 241 (–1.43), 293 (–1.54)
**13a**	+10.0 (0.10)	(0.05): 203 (–13.12), 211 (12.44), 225 (–4.03), 242 (1.25), 292 (2.14)
**13b**	–12.0 (0.05)	(0.025): 203 (5.80), 212 (–7.55), 225 (2.23), 242 (–1.07), 292 (–1.49)
**14a**	+19.0 (0.10)	(0.093): 202 (–8.65), 211 (9.78), 225 (–1.55), 242 (2.89), 294 (1.00)
**14b**	–28.1 (0.10)	(0.06): 202 (15.35), 211 (–18.79), 226 (2.72), 242 (–5.34), 294 (–1.85)
**15a**	+14.0 (0.05)	(0.03): 206 (–27.58), 216 (8.24), 243 (4.48), 292 (1.38)
**15b**	–26.1 (0.05)	(0.035): 206 (22.08), 216 (–12.89), 242 (–5.31), 292 (–2.89)
**16a**	+38.7 (0.06)	(0.12): 204 (9.09), 216 (–6.46), 243 (4.07), 273 (1.17)
**16b**	–12.0 (0.04)	(0.08): 204 (–5.19), 216 (2.84), 243 (–2.35), 273 (–0.54)
**17a**	–40.2 (0.10)	(0.10): 197 (15.86), 208 (–10.99), 238 (–5.45)
**17b**	+25.1 (0.10)	(0.10): 197 (–12.93), 208 (8.68), 237 (4.29)
**18a**	–19.0 (0.10)	(0.045): 207 (3.87), 219 (–0.77), 246 (–2.81)
**18b**	+23.0 (0.10)	(0.045): 207 (–4.52), 219 (0.41), 246 (2.73)

[a] Measured in $CHCl_3$; [b] Measured in MeOH.

Table S4. Structural classes of all the isolated compounds

No.	Structural class (subclass/type)
**1**	terpenoid (monoterpenoid/iridoid)
**2**	terpenoid (monoterpenoid/iridoid)
**3**	terpenoid (monoterpenoid/iridoid)
**4**	terpenoid (monoterpenoid/iridoid)
**5**	terpenoid (monoterpenoid/iridoid)
**6**	terpenoid (monoterpenoid/iridoid)
**7**	terpenoid (monoterpenoid/iridoid)
**8**	terpenoid (monoterpenoid/iridoid)
**9**	lignan (oxyneolignan/8-O-4′ type)
**10**	lignan (oxyneolignan/8-O-4′ type)
**11**	lignan (oxyneolignan/8-O-4′ type)
**12**	lignan (benzofuran type)
**13**	lignan (benzofuran type)
**14**	lignan (benzofuran type)
**15**	lignan (benzofuran type)
**16**	lignan (arylnaphthalene type)
**17**	lignan (tetrahydrofuran type)
**18**	lignan (tetrahydrofuran type)
**19**	terpenoid (monoterpenoid/iridoid)
**20**	terpenoid (nor-sesquiterpenoid)
**21**	lignan (neolignan/8-3′ type)
**22**	phenylpropanoid

Materials and Methods

Instrumentation and Reagents

Optical rotations were measured on a Rudolph VI polarimeter (Rudolph Research Analytical, Hackettstown, USA) with a 10 cm length cell. NMR experiments were recorded on a Bruker Avance DRX600 spectrometer (Bruker BioSpin AG, Fallanden, Switzerland) and referenced to residual solvent peaks ($CDCl_3$: δ_H 7.26, δ_C 77.16; methanol-d_4: δ_H 3.31, δ_C 49.00; DMSO-d_6: δ_H 2.50, δ_C 39.52). ESIMS and HR-ESIMS analyses were carried out on Agilent 6460 Triple Quad and 6545 Q-TOF MS instruments (Agilent Technologies Inc., Waldbronn, Germany), respectively. All normal HPLC separations were performed using an Agilent 1260 series LC instrument (Agilent Technologies Inc., Waldbronn, Germany) coupled with an Agilent SB-C_{18} column (9.4 × 250 mm, Agilent Technologies Inc., Santa Clara, USA) unless specified. Column chromatography (CC) was performed on D101-macroporous absorption resin (Sinopharm Chemical Reagent Co., Ltd., Shanghai), MCI gel (CHP20P, Mitsubishi Chemical Corporation, Tokyo, Japan), reversed phase C_{18} silica gel (Merck KGaA, Darmstadt, Germany), Sephadex LH-20 (GE Healthcare Bio-Sciences AB, Uppsala, Sweden) and silica gel (300–400 mesh; Qingdao Marine Chemical Co. Ltd., Qingdao, China). All solvents used for CC were of analytical grade (Tianjin Fuyu Fine Chemical Co. Ltd., Tianjin, China) and solvents used for HPLC were of HPLC grade (Oceanpak Alexative Chemical Ltd., Goteborg, Sweden). Pre-coated silica gel GF_{254} plates (Qingdao Marine Chemical Co. Ltd., Qingdao, China) were used for TLC monitoring. The optical density (OD) was measured on a Microplate Reader (Tecan, Switzerland).

ECD calculation

The ChemDraw Pro 14.1 software with MM2 force field was used to establish the initial conformations of compounds 16 and 21. Conformational searches using mixed torsional/Low-mode sampling method with MMFFs force field in an energy window of 3.01 kcal/mol were carried out by means of the conformational search module in the Maestro 10.2 software. The re-optimization and the following TD-DFT calculations of the re-optimized conformations were all performed with Gaussian 09 software at the B3LYP/6-311G(d,p) level *in vacuo* for **16** and **21**. Frequency analysis was performed as well to confirm that the re-optimized conformers were at the energy minima. Finally, the SpecDis 1.70 software was used to obtain the Boltzmann-averaged ECD spectra. The calculated ECD spectra were then compared with the experimental ones.

Figure S1. $\Delta\delta$ (δ_S–δ_R) values in ppm of MTPA esters for **5** and **8**.

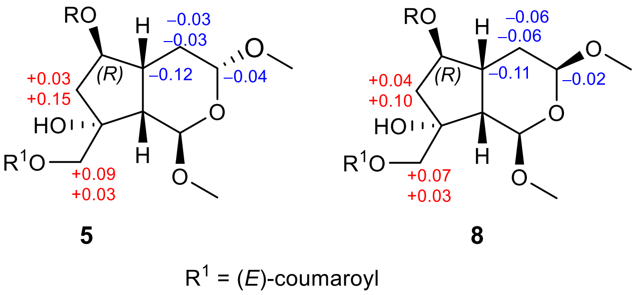

Figure S2. Conformations of the Mo_2^{4+} complexes for **5r** and **8r**, and their respective ECD spectrum.

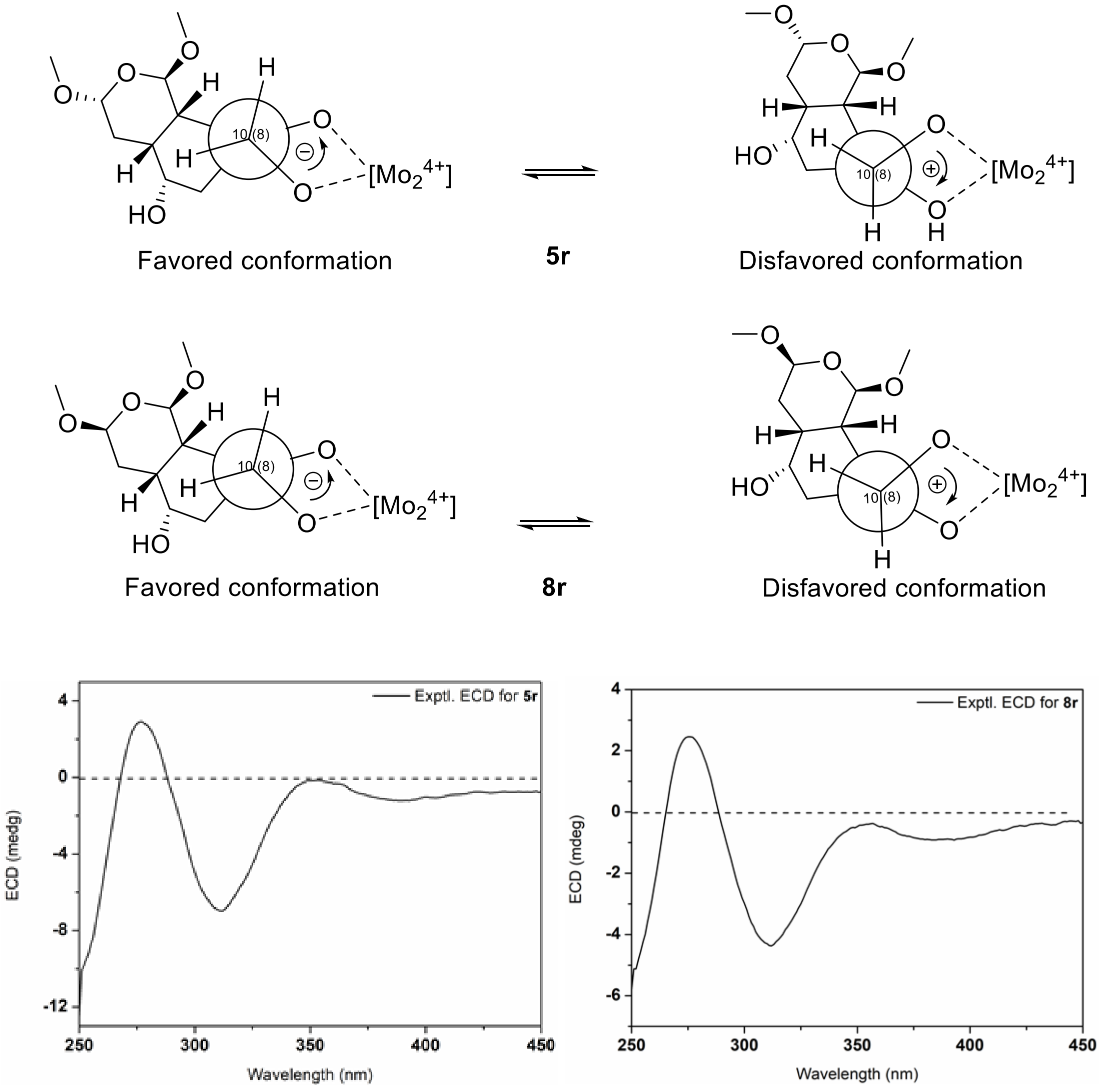

Figure S3. The ^{1}H NMR spectrum of **1** (CD_3OD).

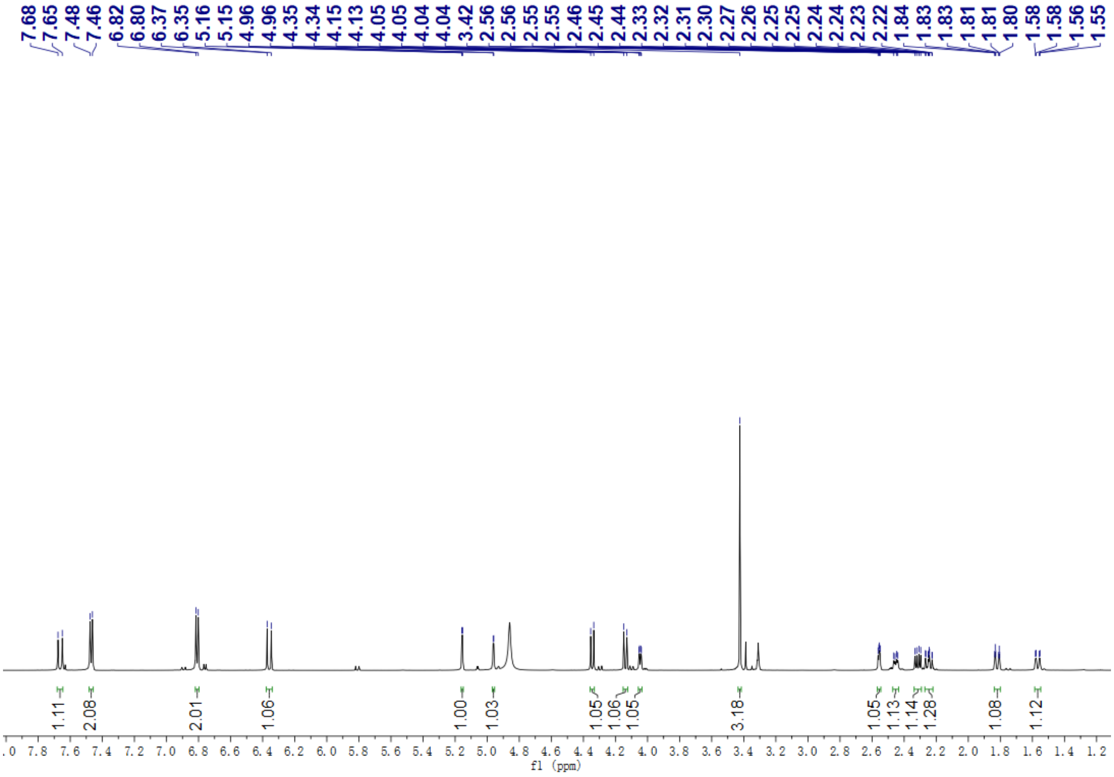

Figure S4. The ^{13}C and DEPT NMR spectra of **1** (CD_3OD).

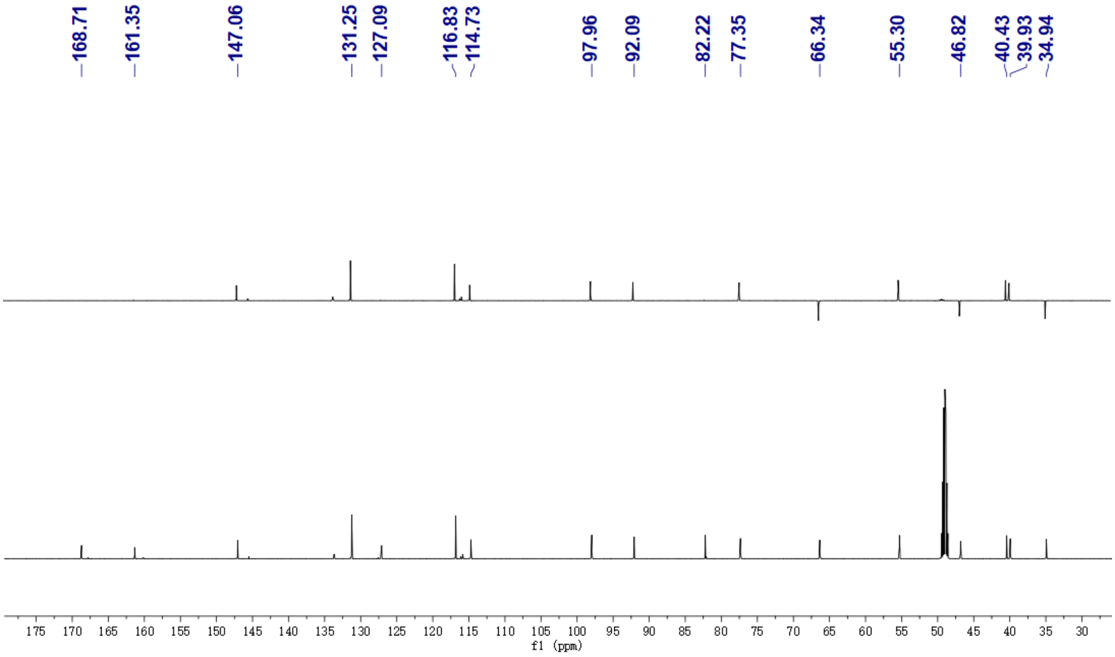

Figure S5. The ^{1}H-^{1}H COSY spectrum of **1** (CD_3OD).

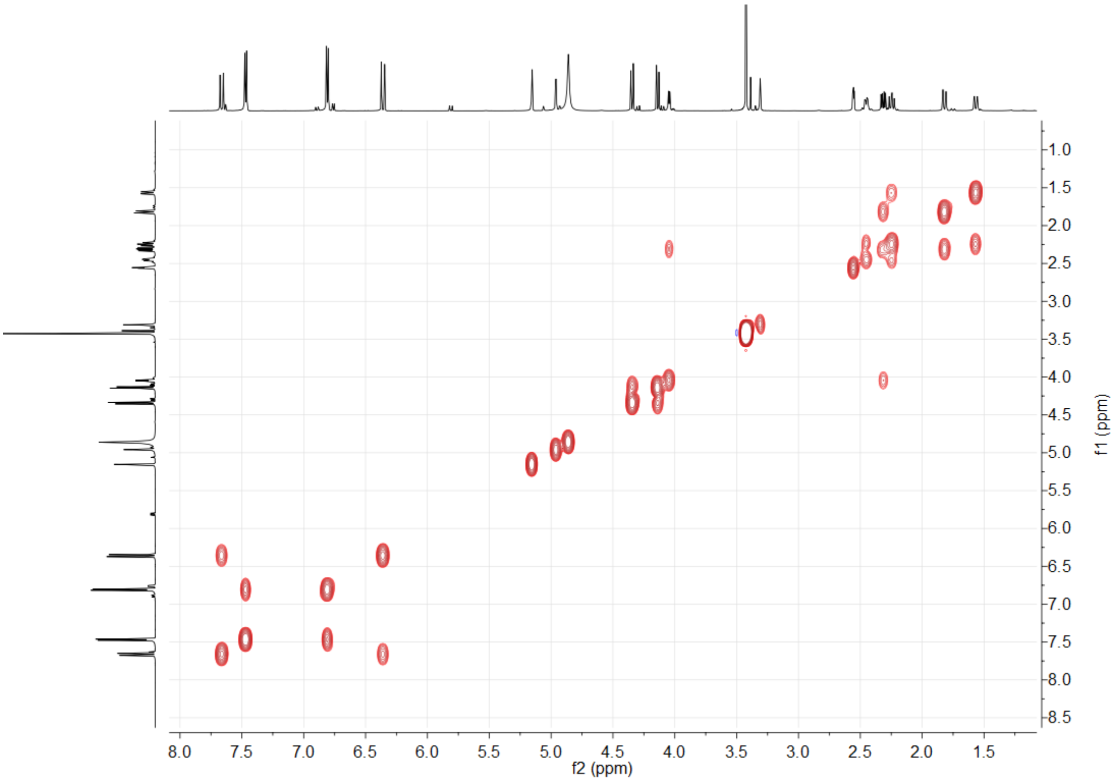

Figure S6. The HSQC spectrum of **1** (CD_3OD).

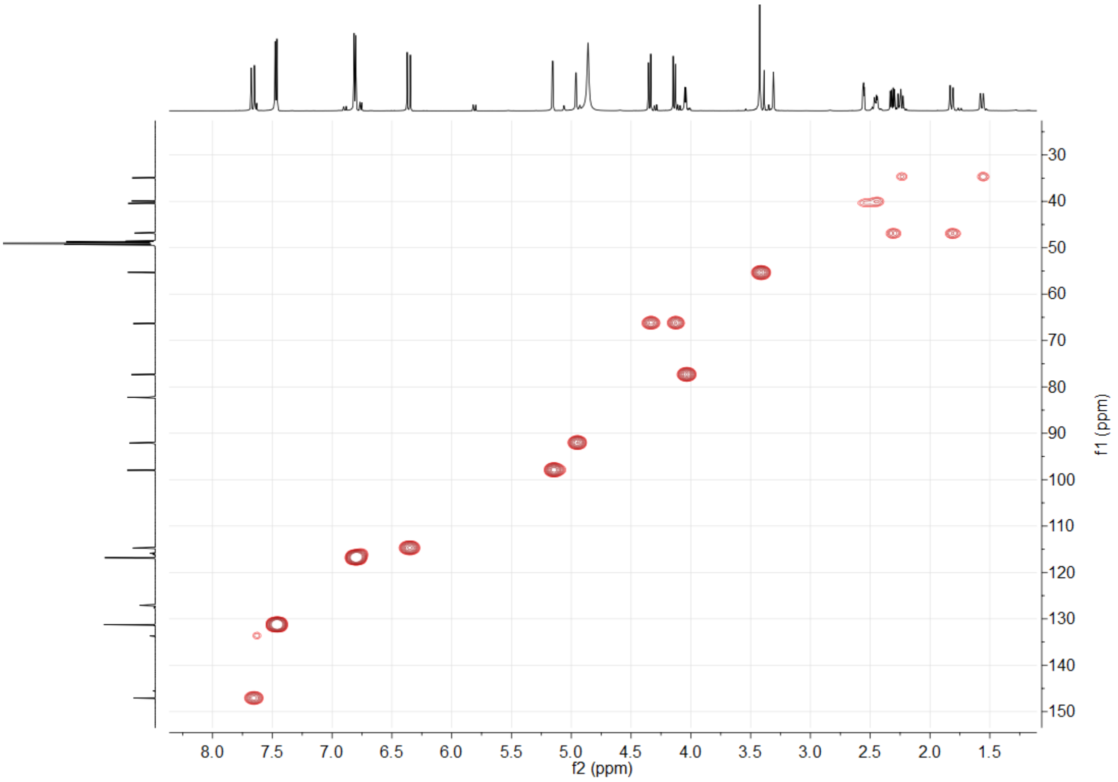

Figure S7. The HMBC spectrum of **1** (CD_3OD).

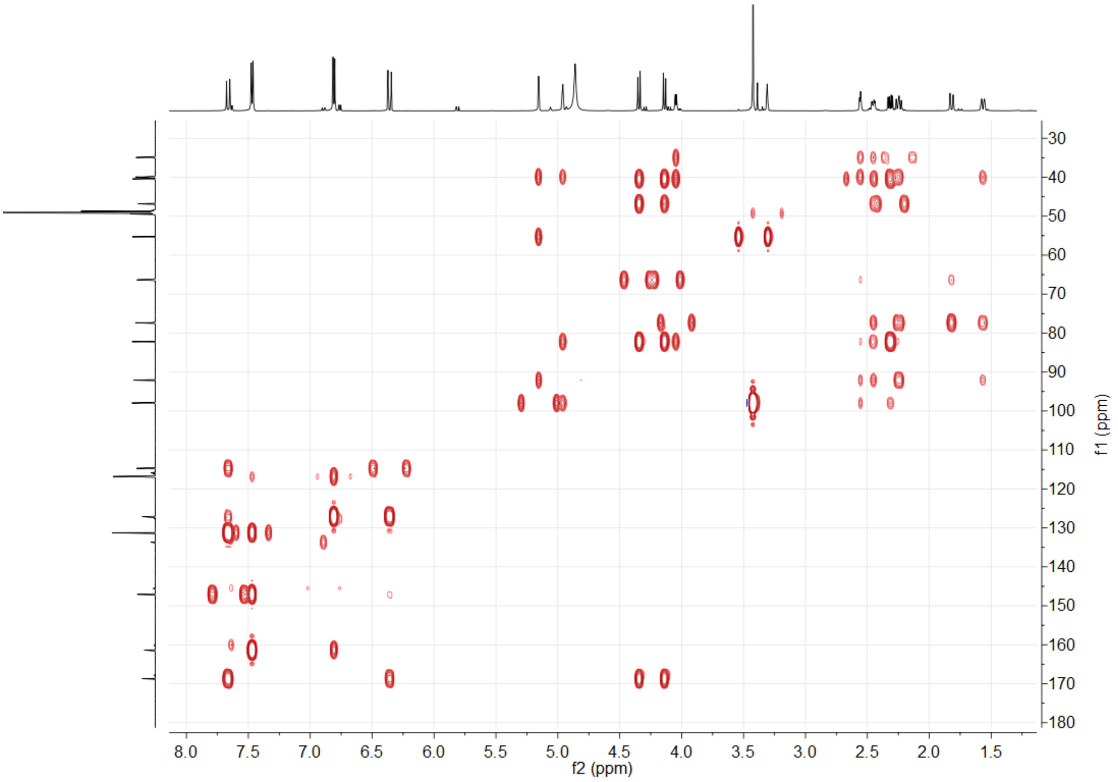

Figure S8. The NOESY spectrum of **1** (CD_3OD).

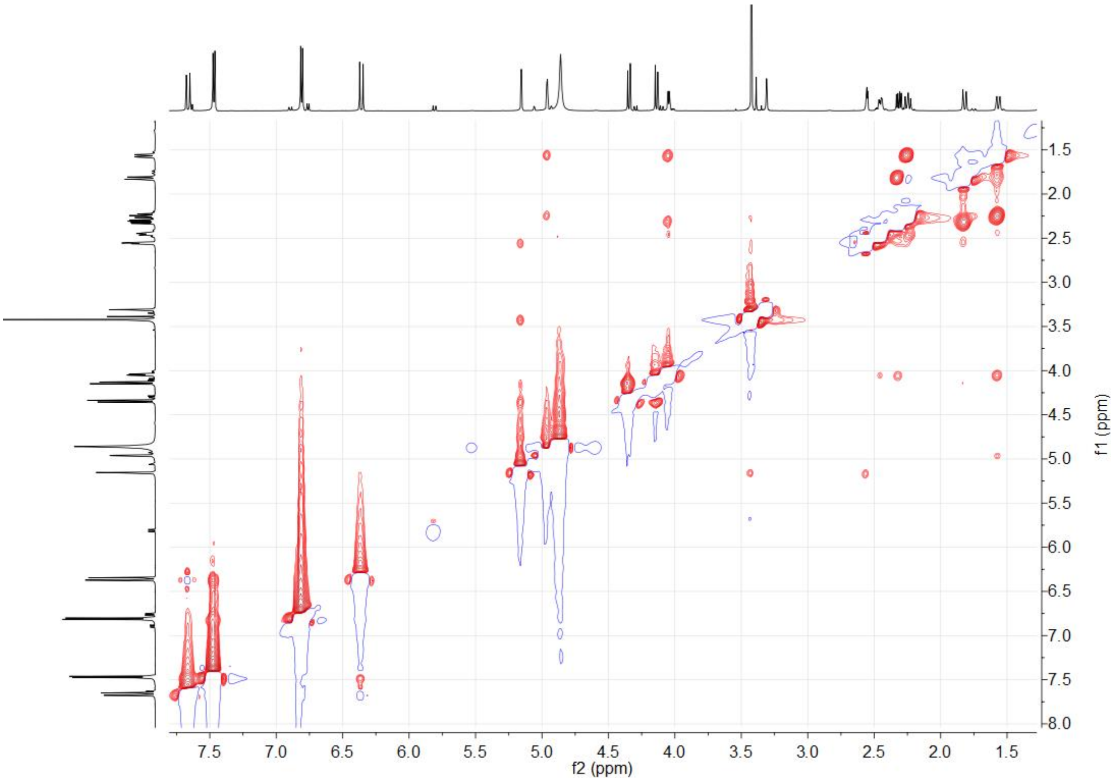

Figure S9. The (+)-HR-ESIMS spectrum of **1**.

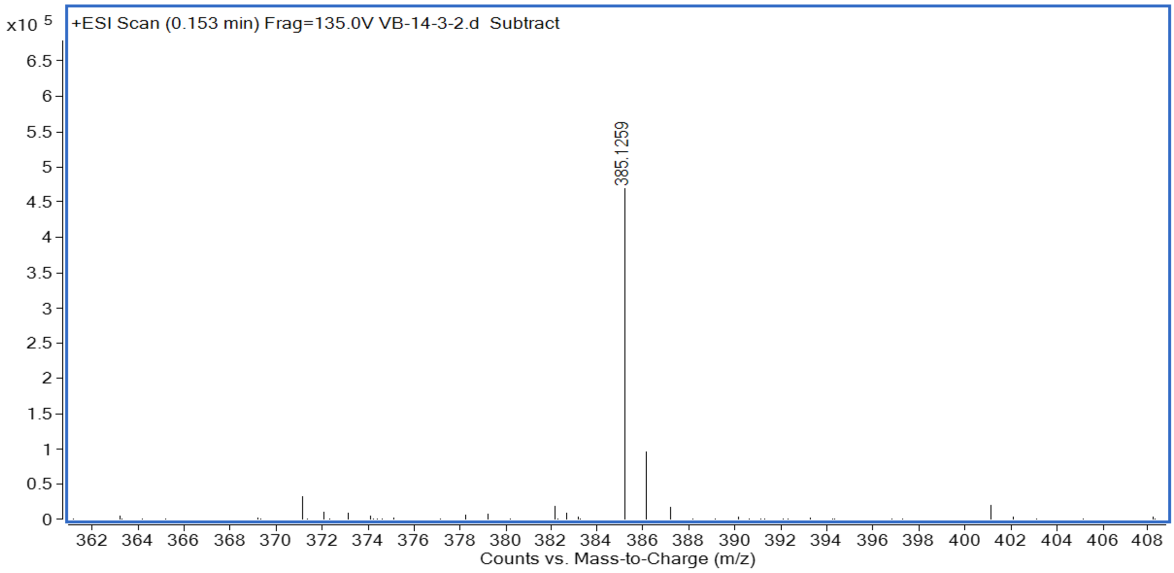

Figure S10. The ^{1}H NMR spectrum of **2** (CD_3OD).

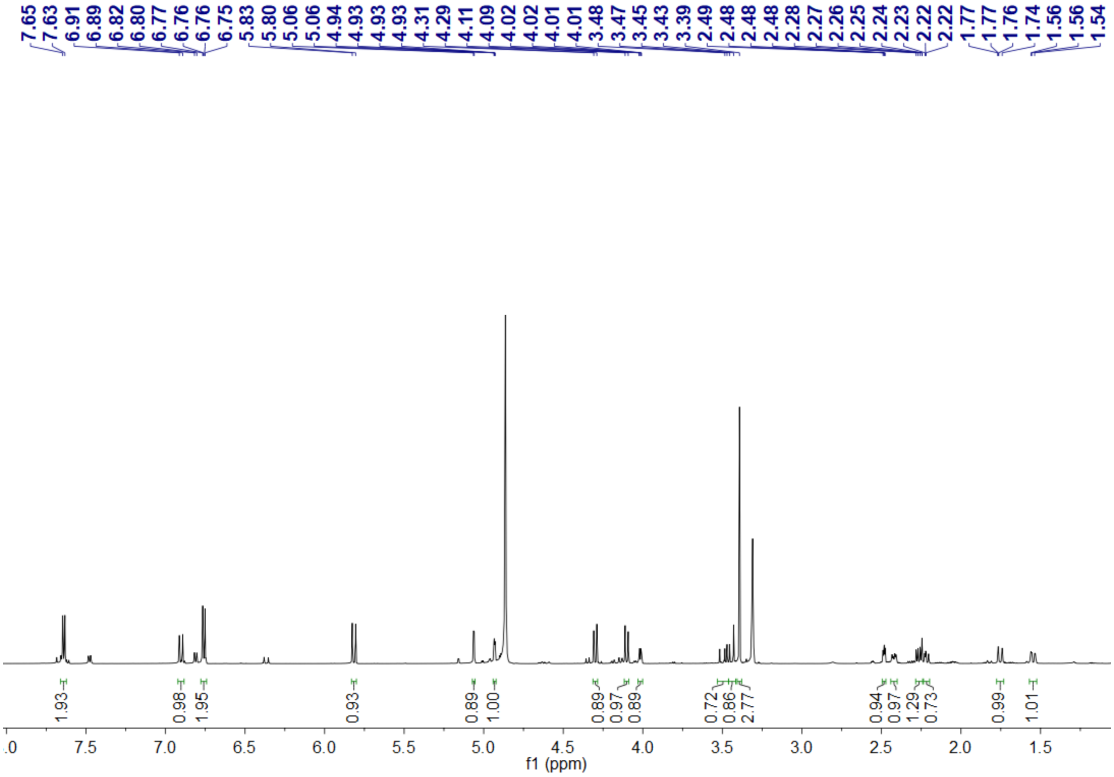

Figure S11. The ^{13}C and DEPT NMR spectra of **2** (CD_3OD).

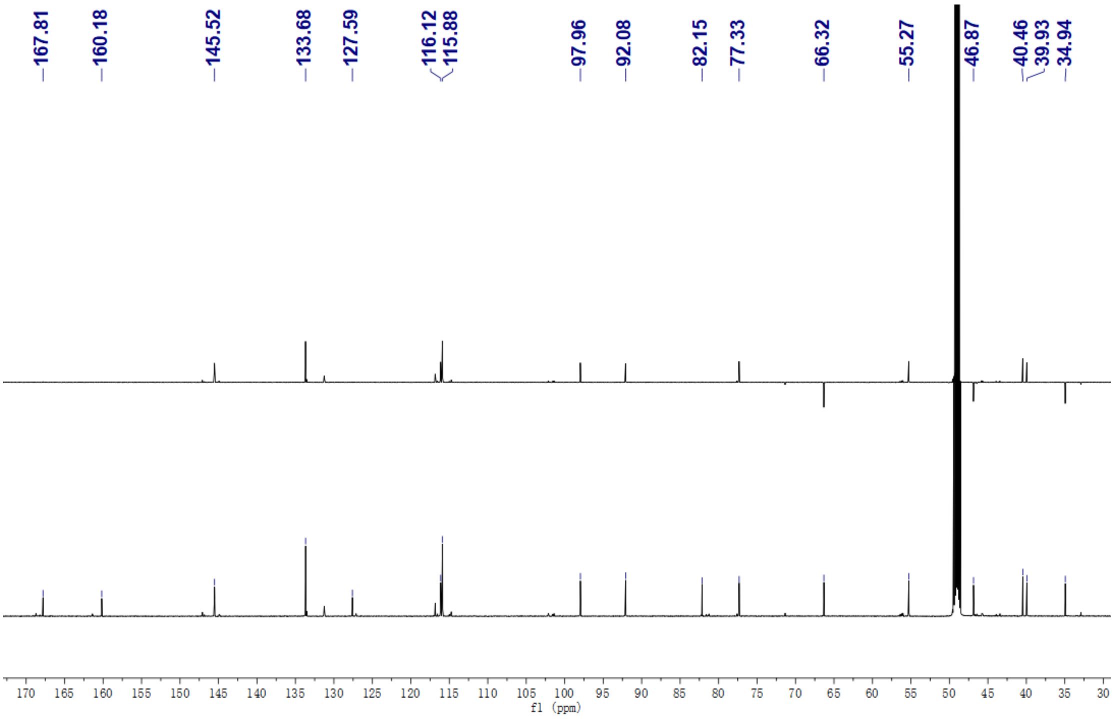

Figure S12. The ^{1}H-^{1}H COSY spectrum of **2** (CD_3OD).

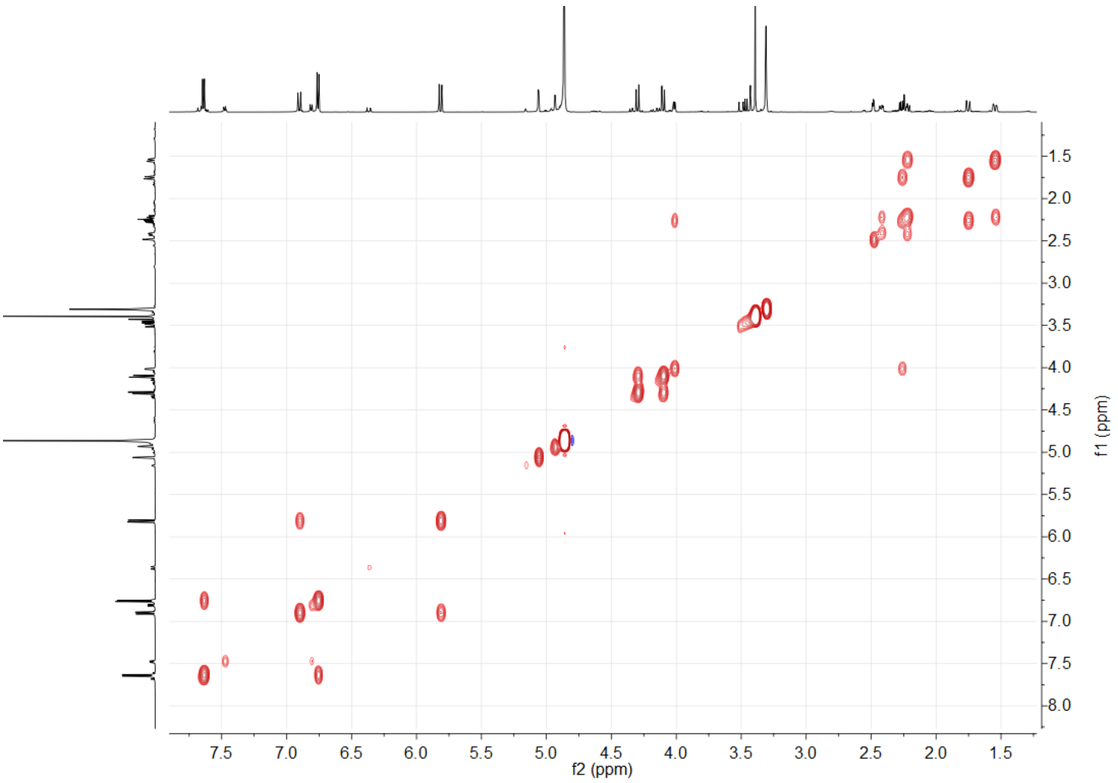

Figure S13. The HSQC spectrum of **2** (CD_3OD).

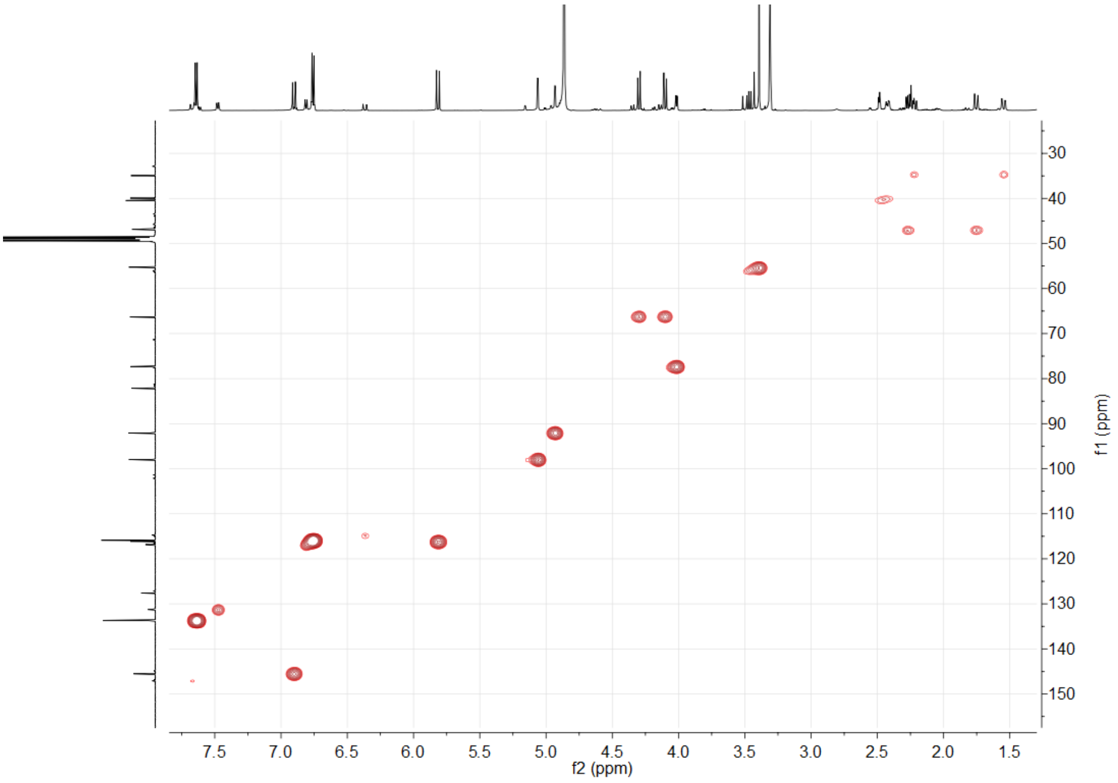

Figure S14. The HMBC spectrum of **2** (CD_3OD).

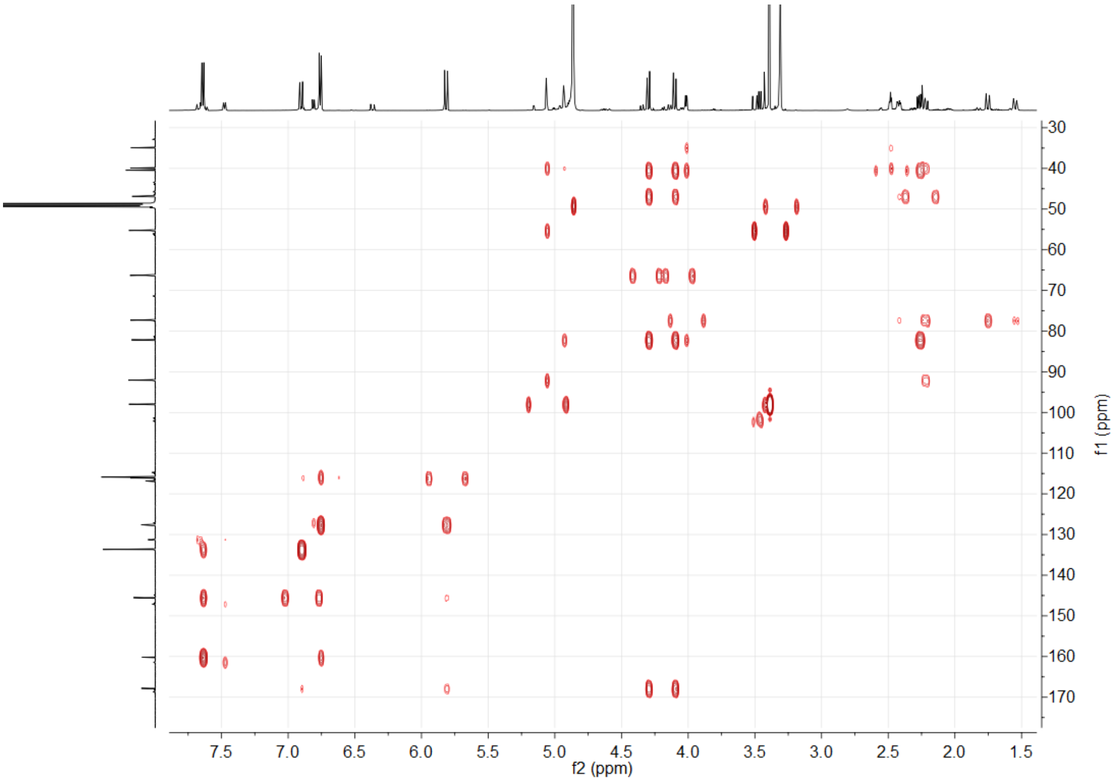

Figure S15. The NOESY spectrum of **2** (CD_3OD).

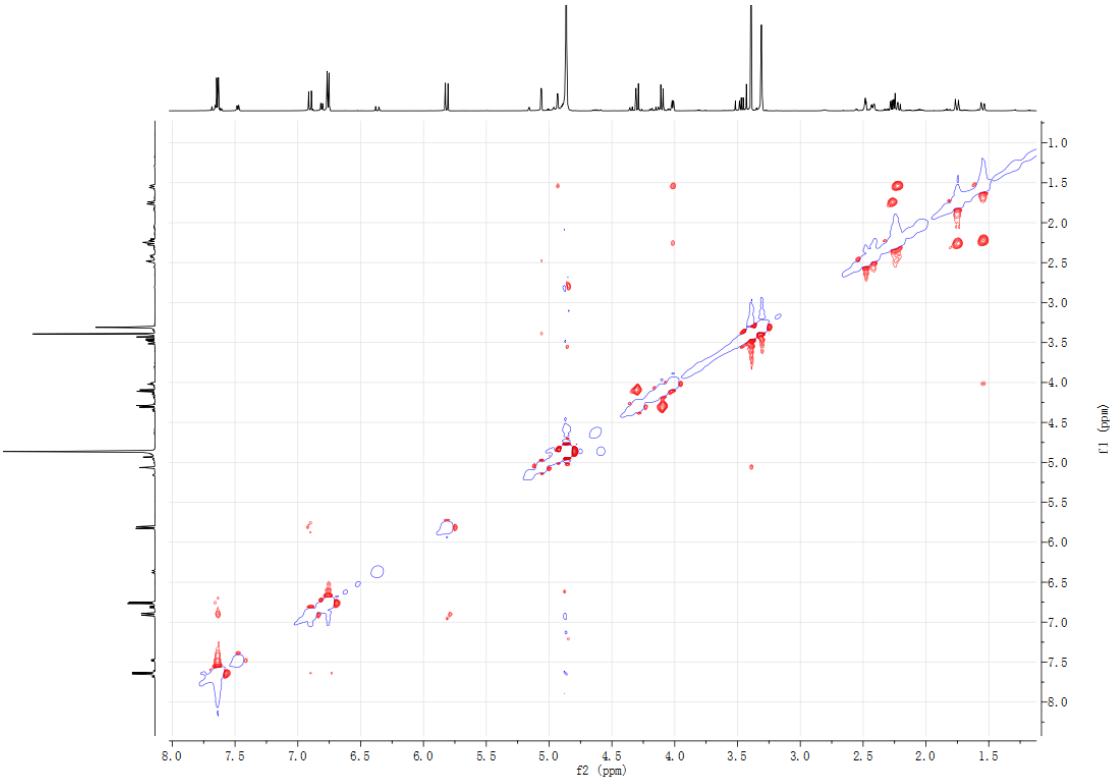

Figure S16. The (+)-HR-ESIMS spectrum of **2**.

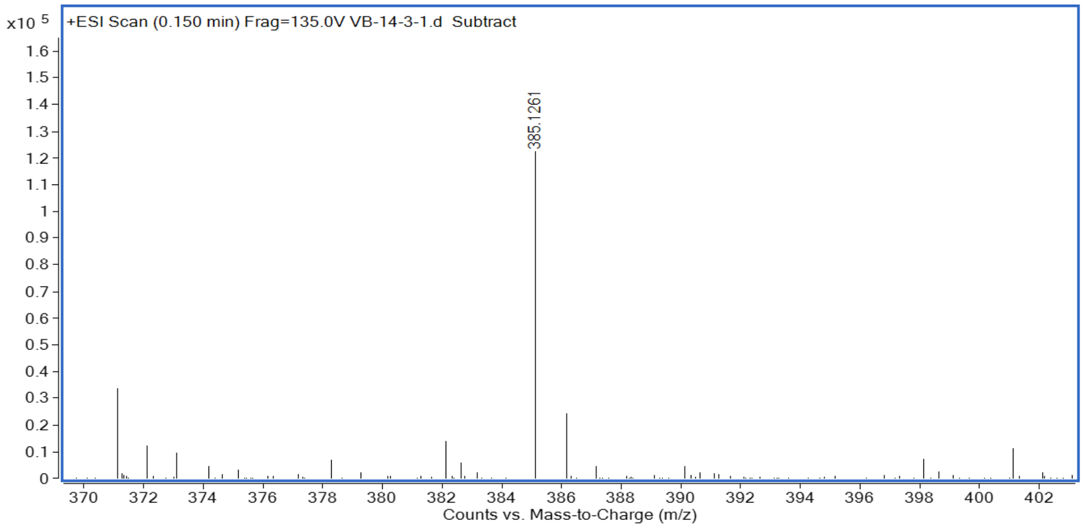

Figure S17. The ^{1}H NMR spectrum of **3** (CD_3OD).

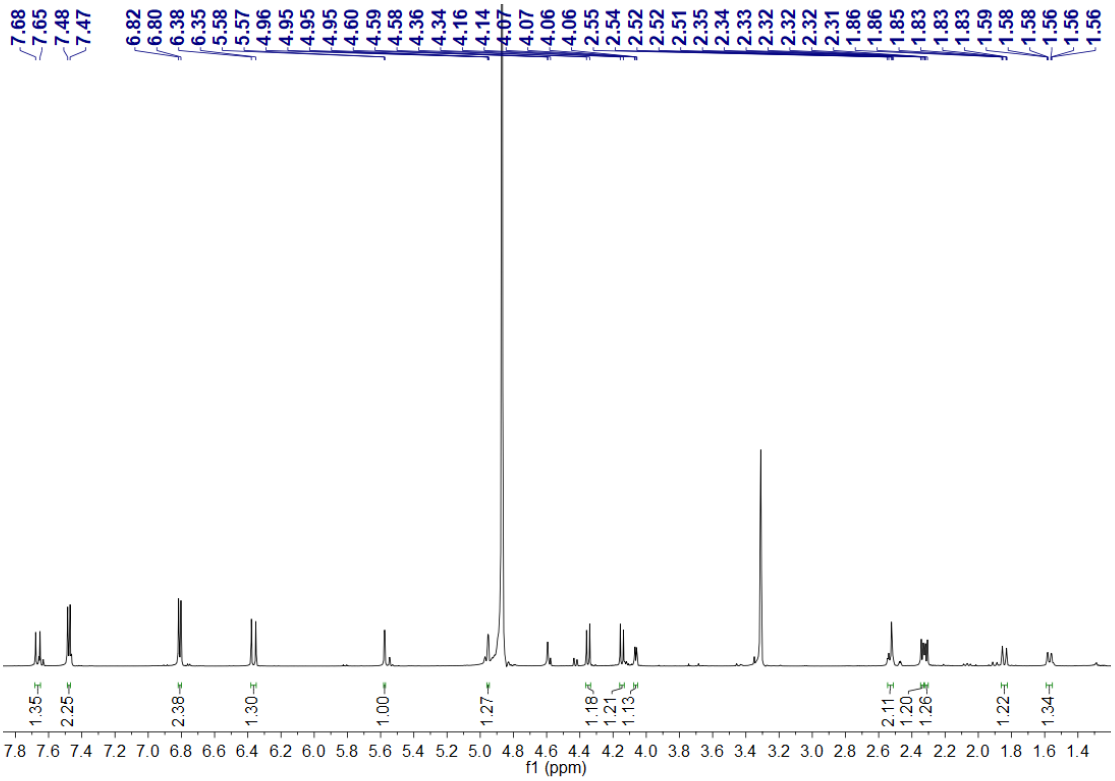

Figure S18. The ^{13}C and DEPT NMR spectra of **3** (CD_3OD).

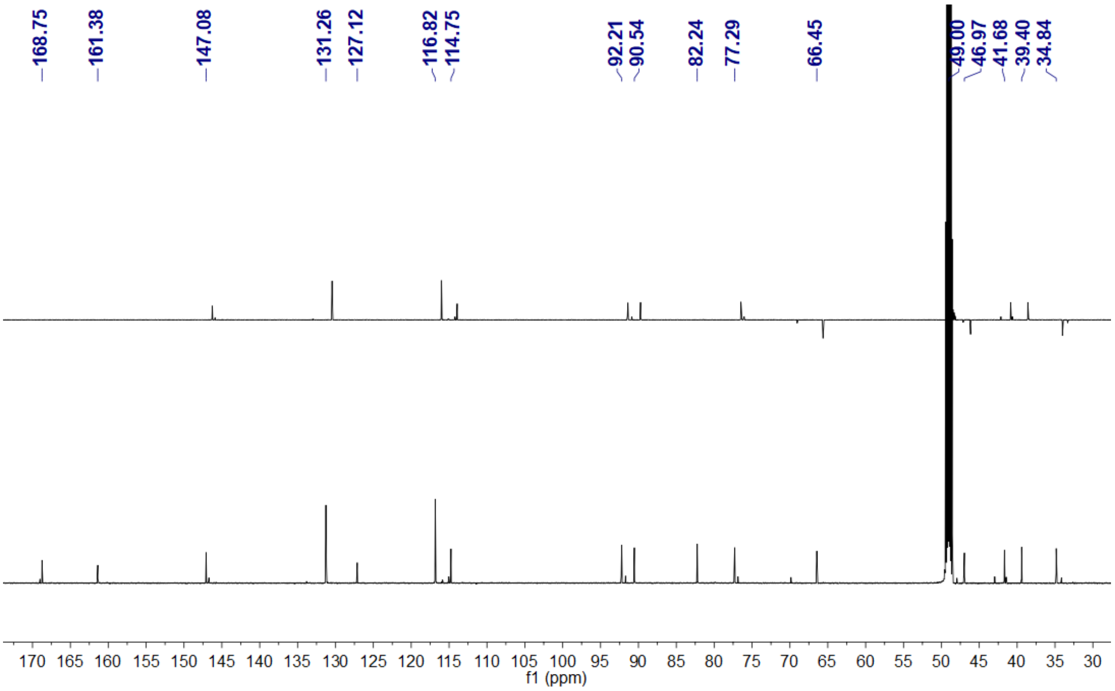

Figure S19. The ^{1}H-^{1}H COSY spectrum of **3** (CD_3OD).

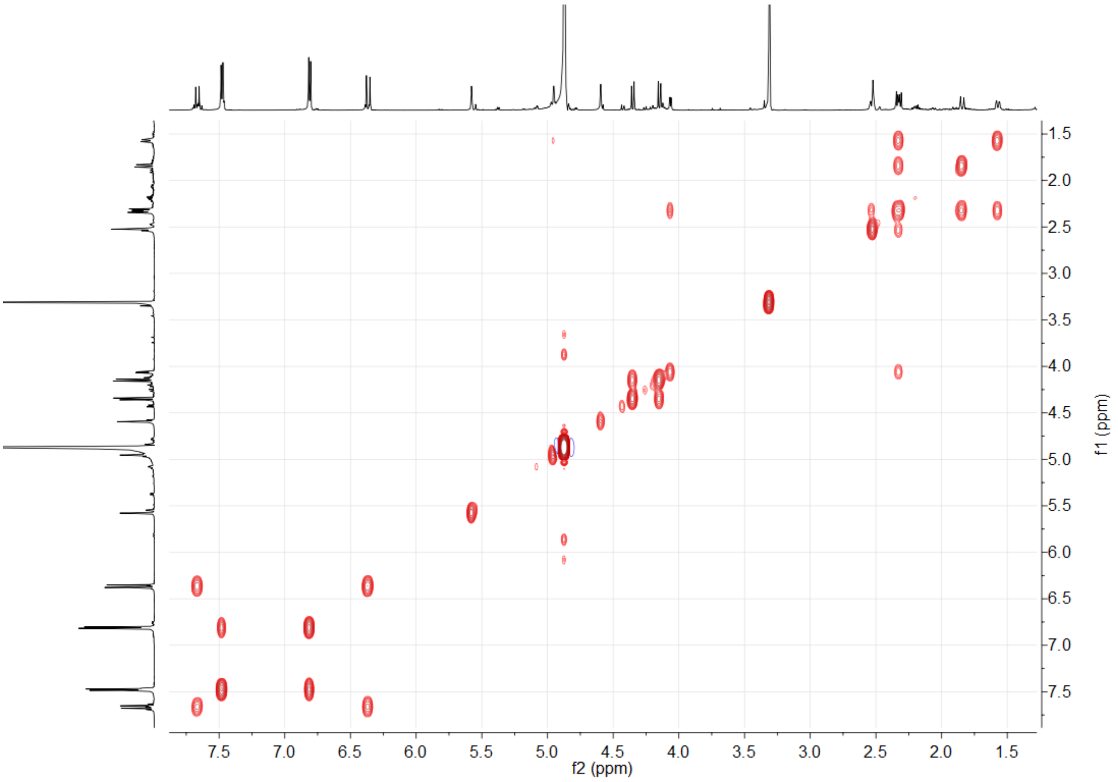

Figure S20. The HSQC spectrum of **3** (CD_3OD).

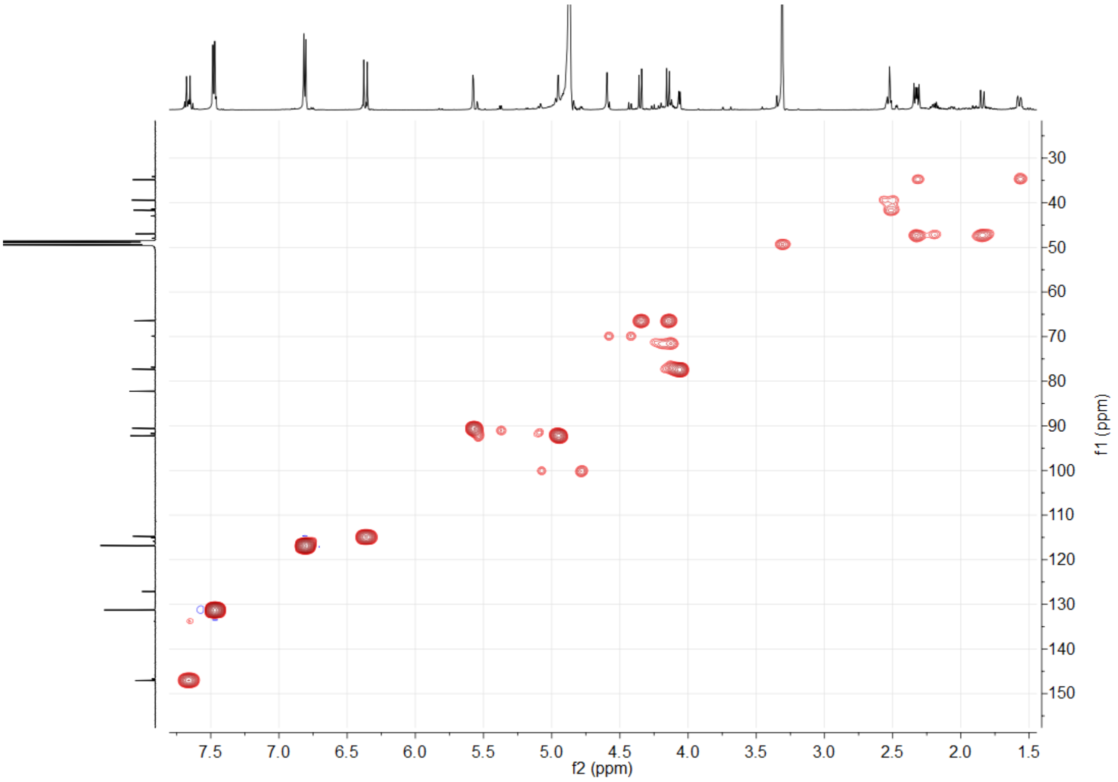

Figure S21. The HMBC spectrum of **3** (CD_3OD).

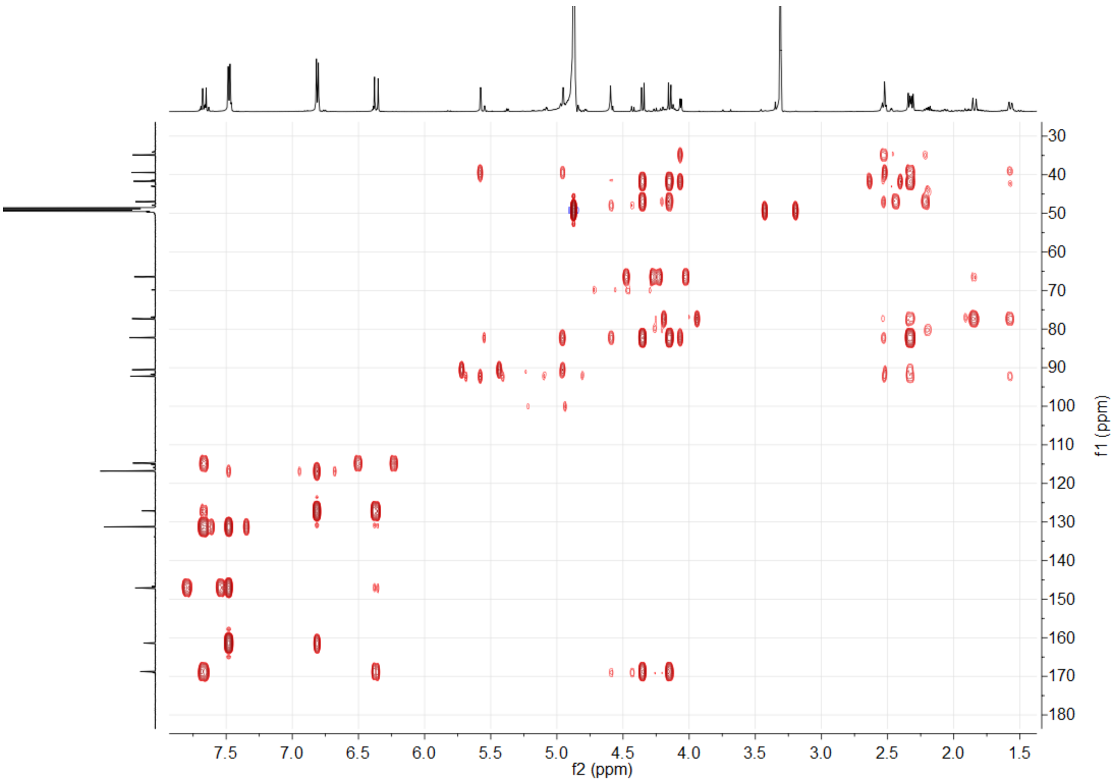

Figure S22. The NOESY spectrum of **3** (CD_3OD).

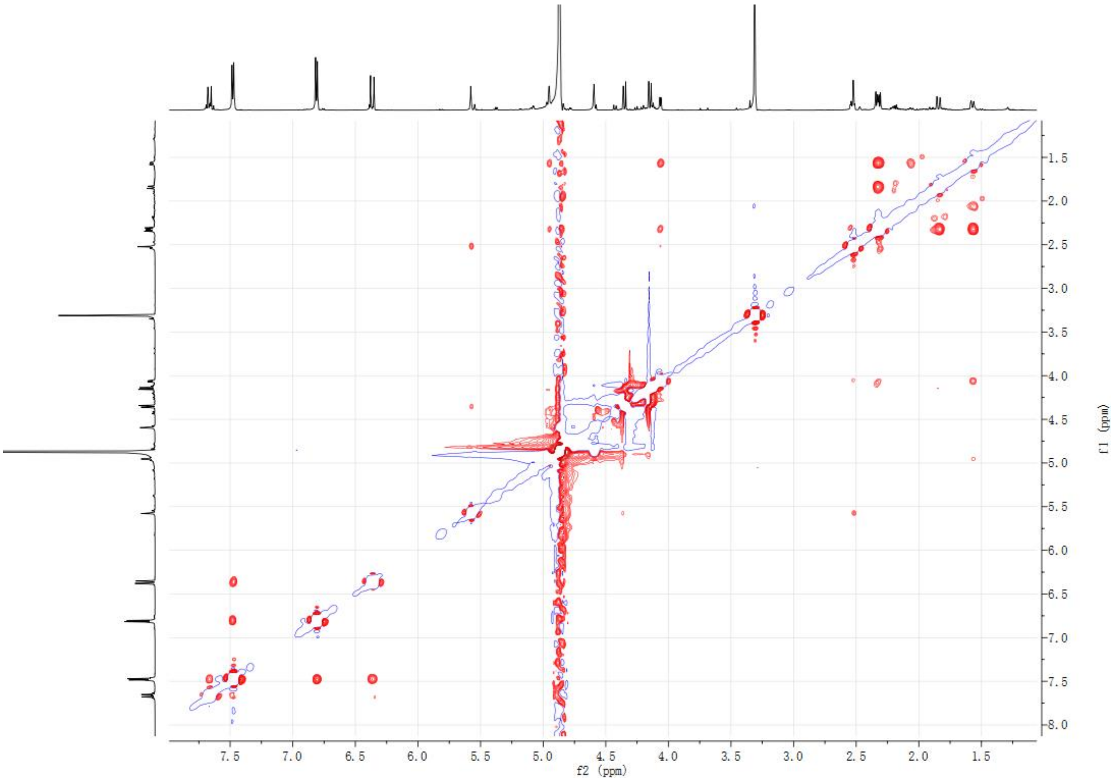

Figure S23. The (+)-HR-ESIMS spectrum of **3**.

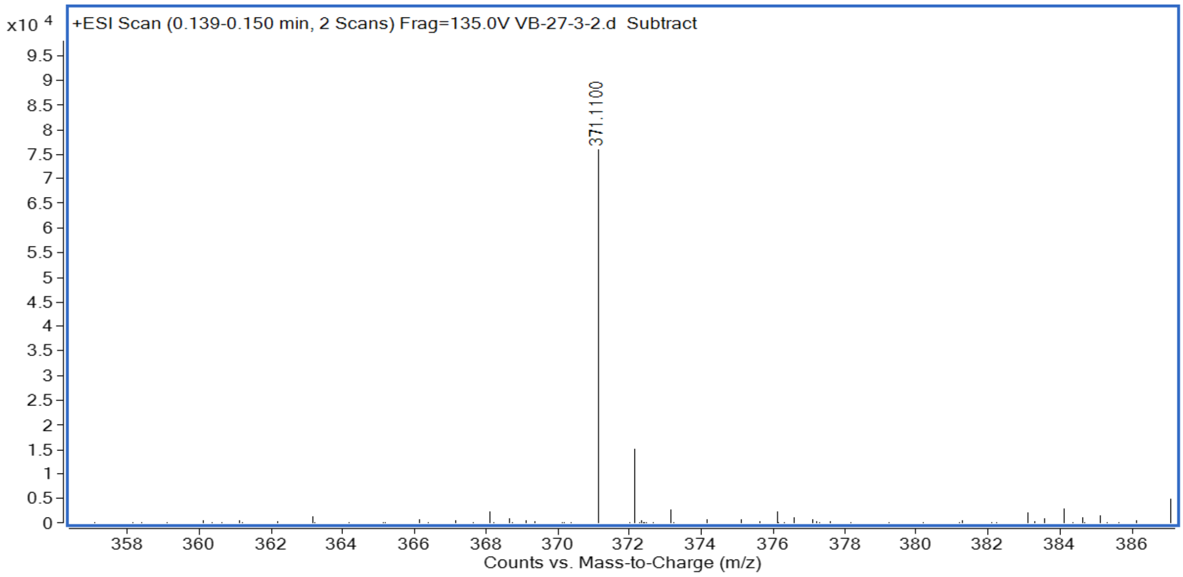

Figure S24. The ^{1}H NMR spectrum of **4** (CD_3OD).

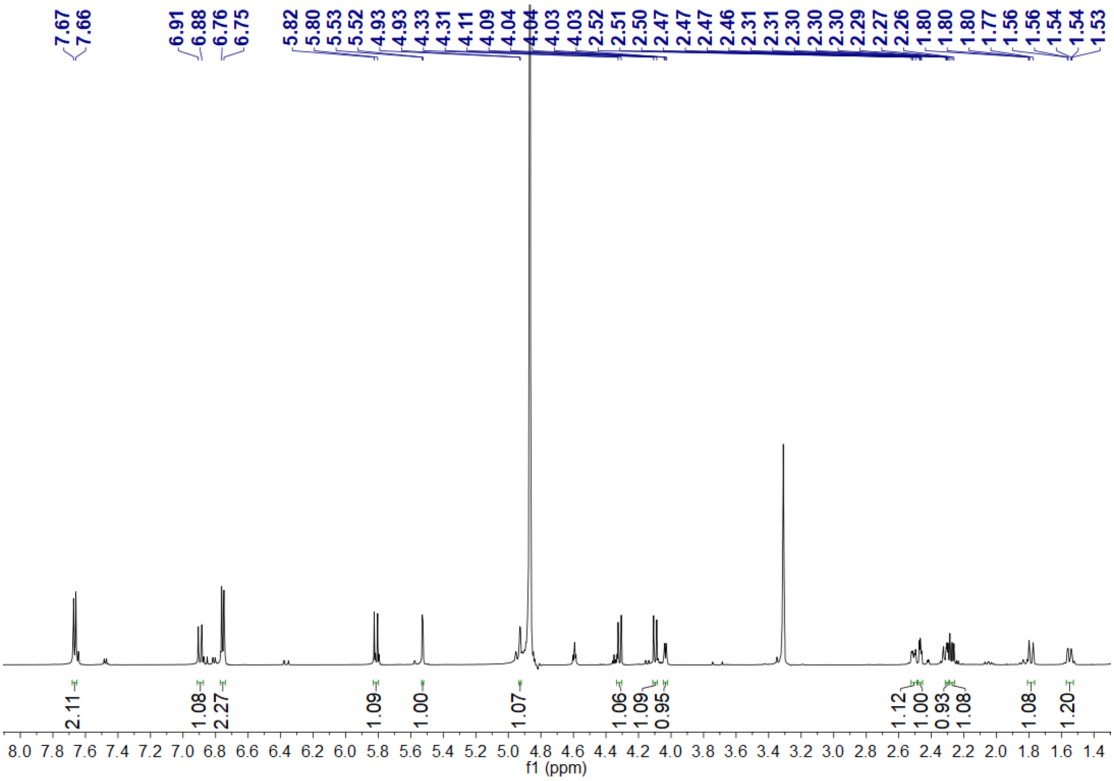

Figure S25. The ^{13}C and DEPT NMR spectra of **4** (CD_3OD).

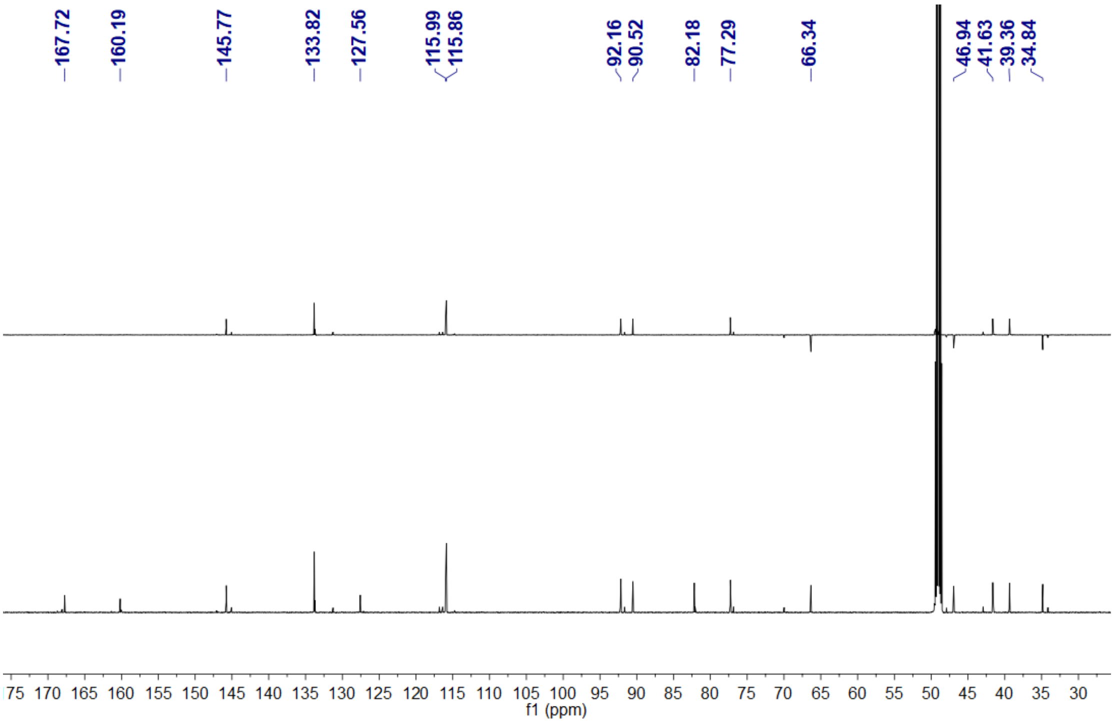

Figure S26. The ¹H-¹H COSY spectrum of **4** (CD_3OD).

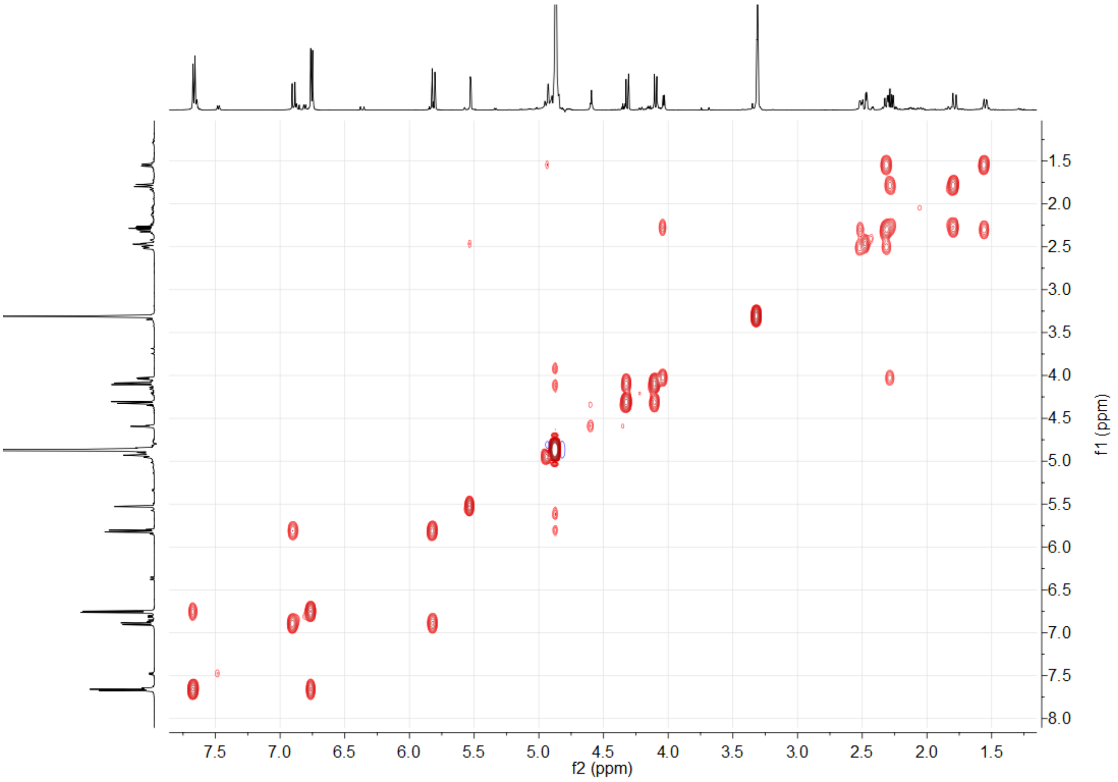

Figure S27. The HSQC spectrum of **4** (CD_3OD).

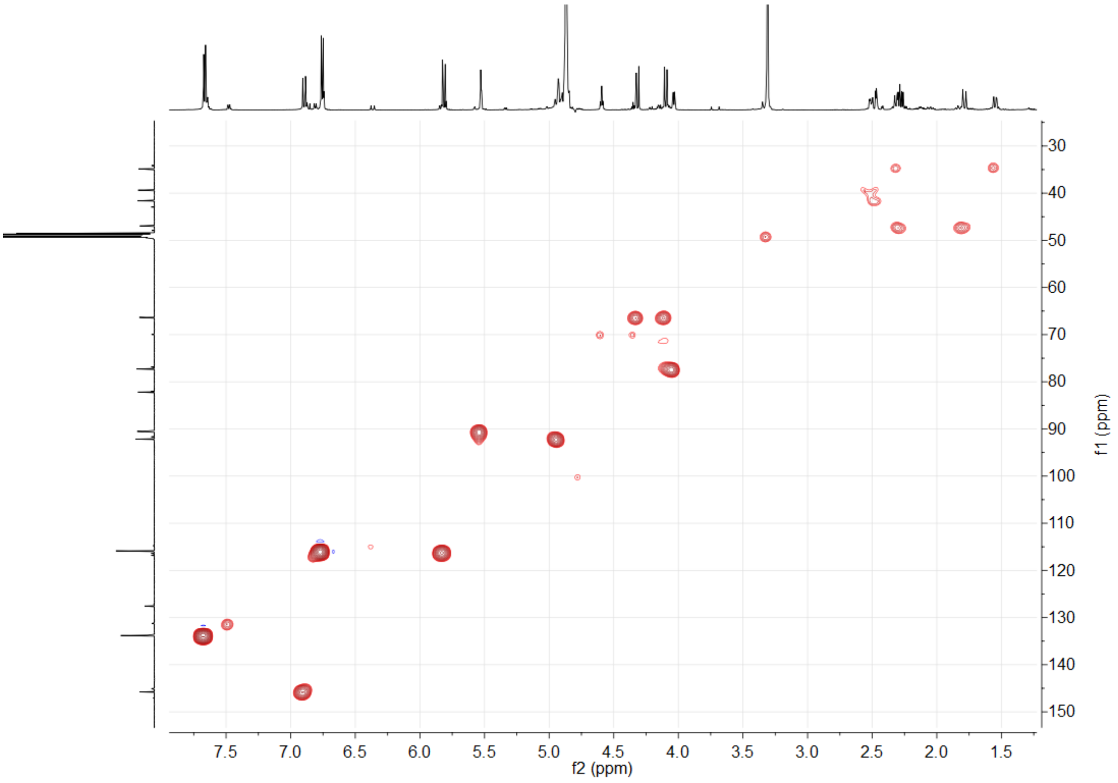

Figure S28. The HMBC spectrum of **4** (CD_3OD).

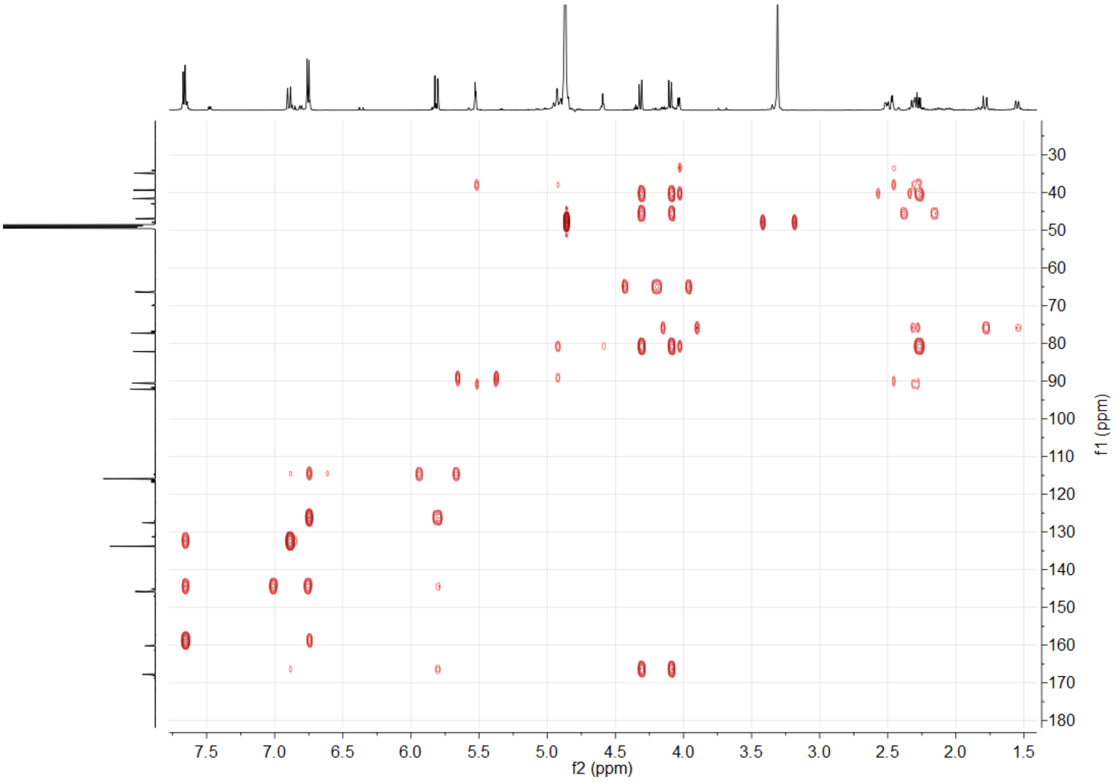

Figure S29. The NOESY spectrum of **4** (CD_3OD).

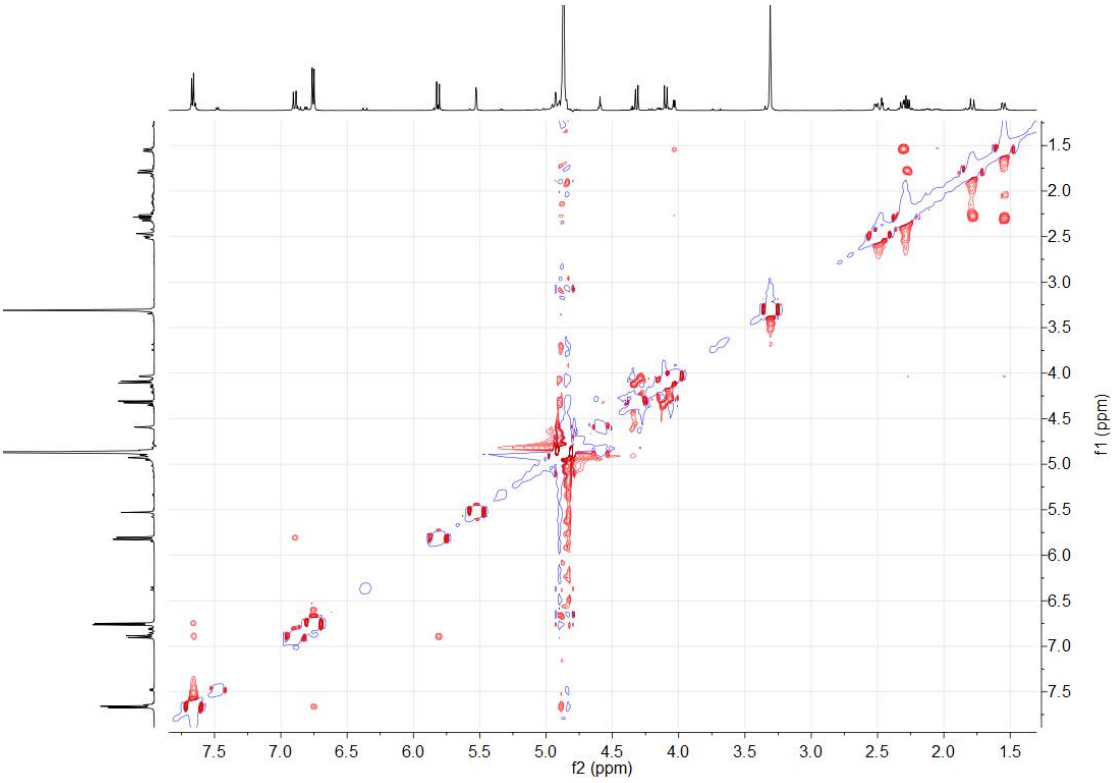

Figure S30. The (+)-HR-ESIMS spectrum of **4**.

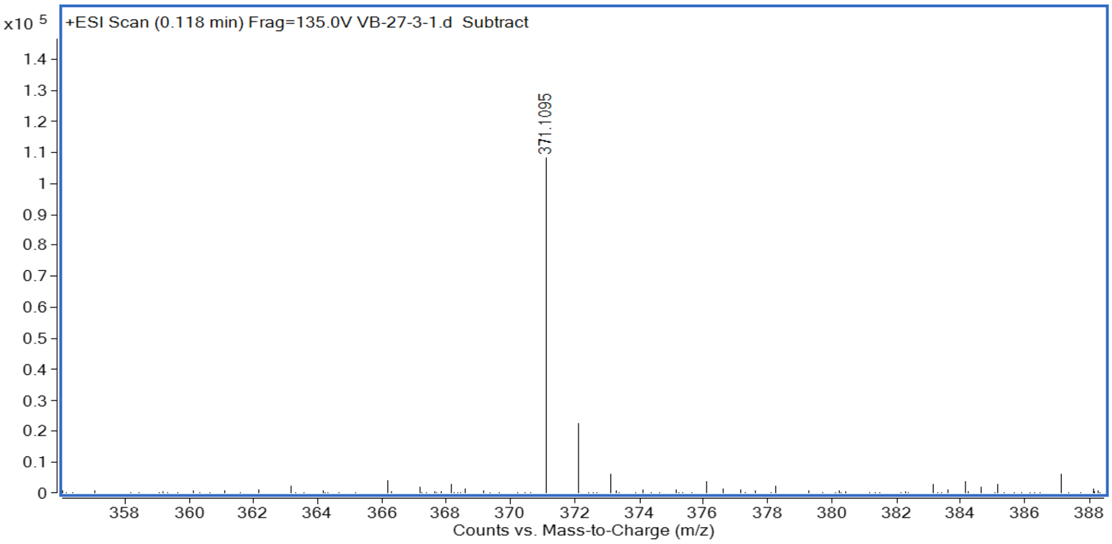

Figure S31. The ^{1}H NMR spectrum of **5** (CD_3OD).

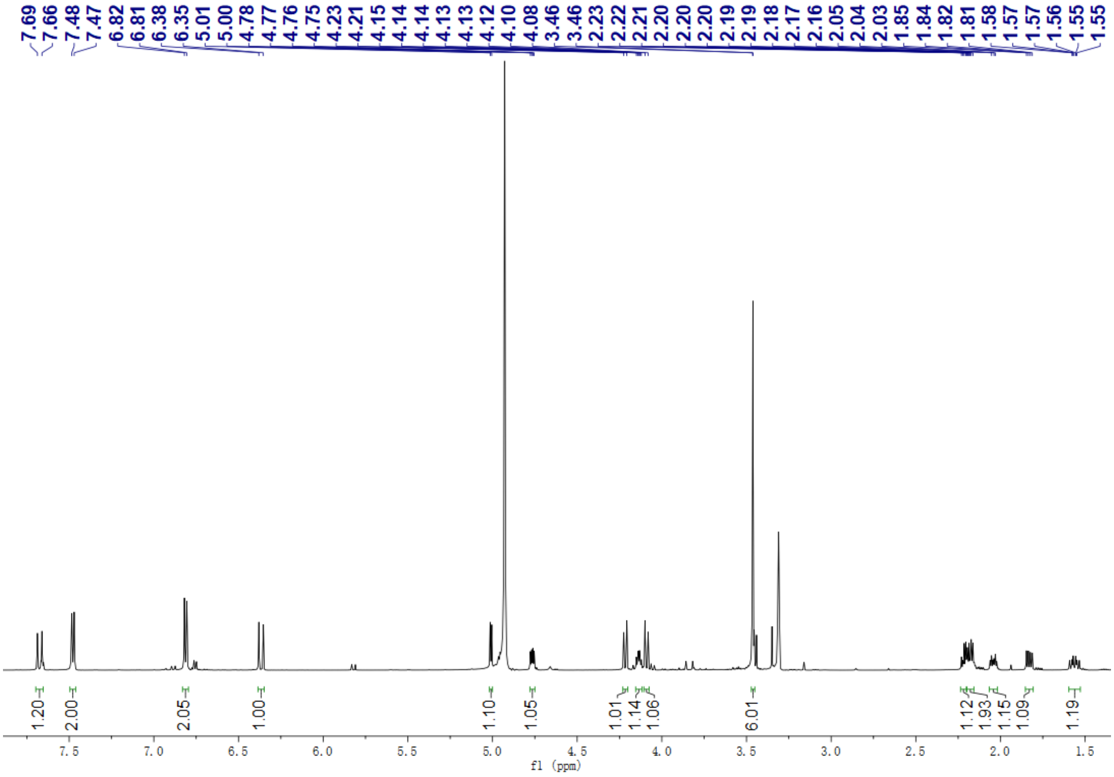

Figure S32. The ^{13}C and DEPT NMR spectra of **5** (CD_3OD, 150 MHz).

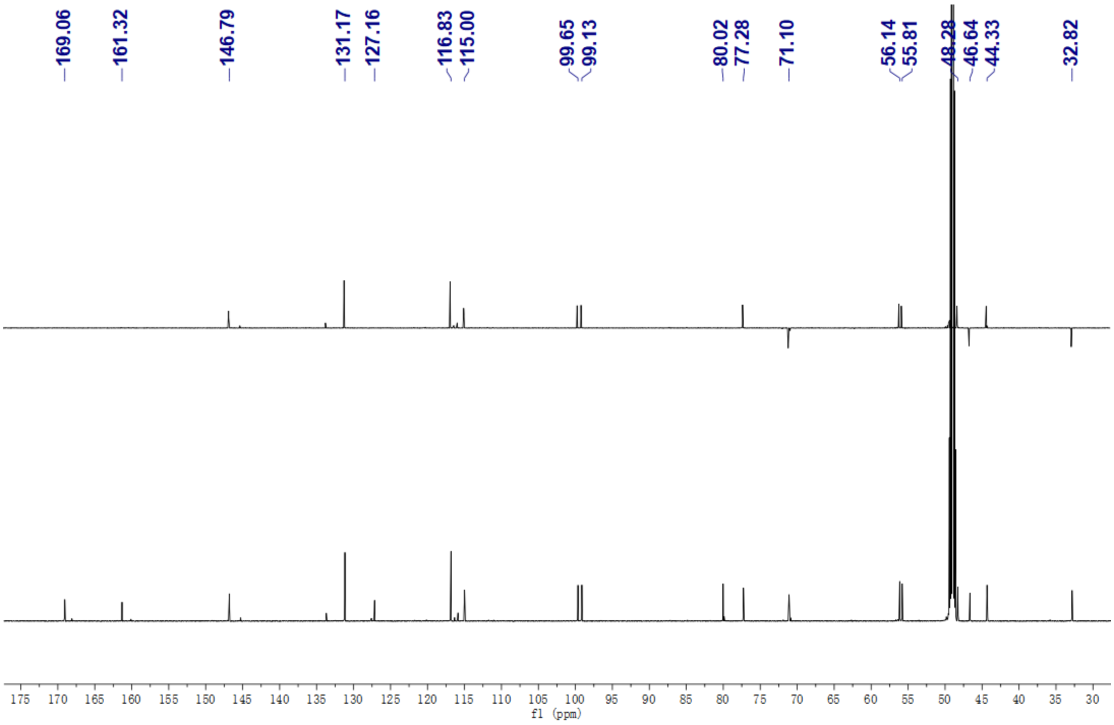

Figure S33. The ^{1}H-^{1}H COSY spectrum of **5** (CD_3OD).

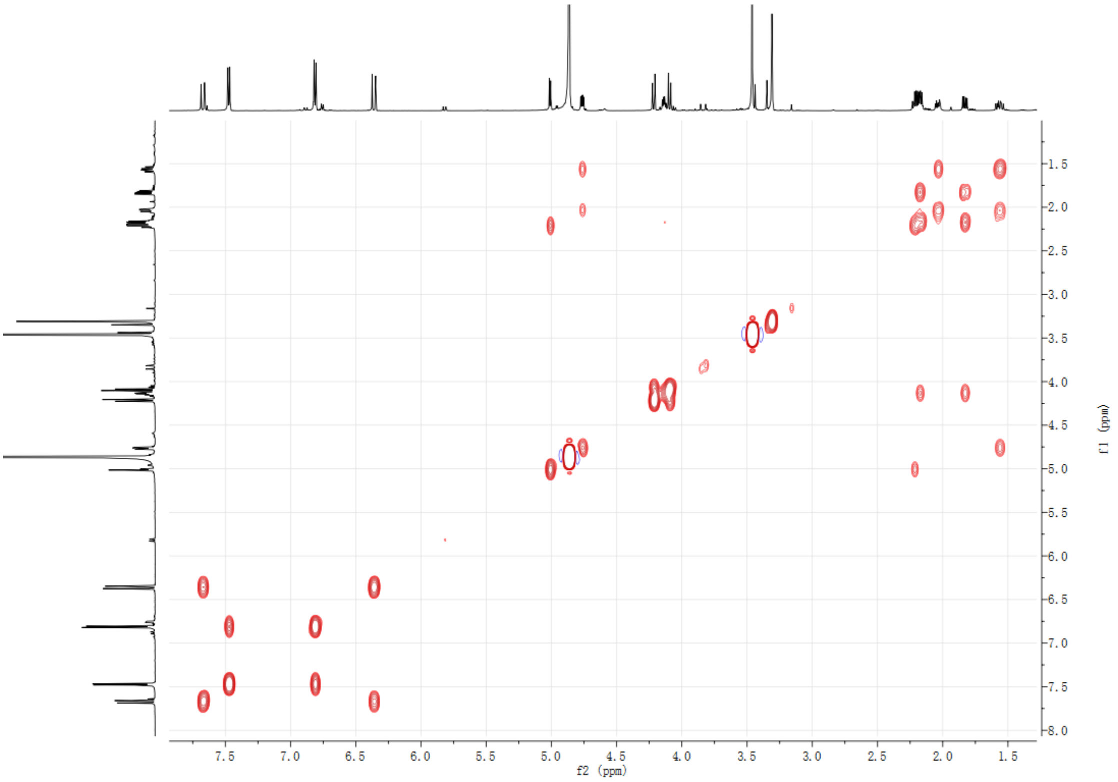

Figure S34. The HSQC spectrum of **5** (CD_3OD).

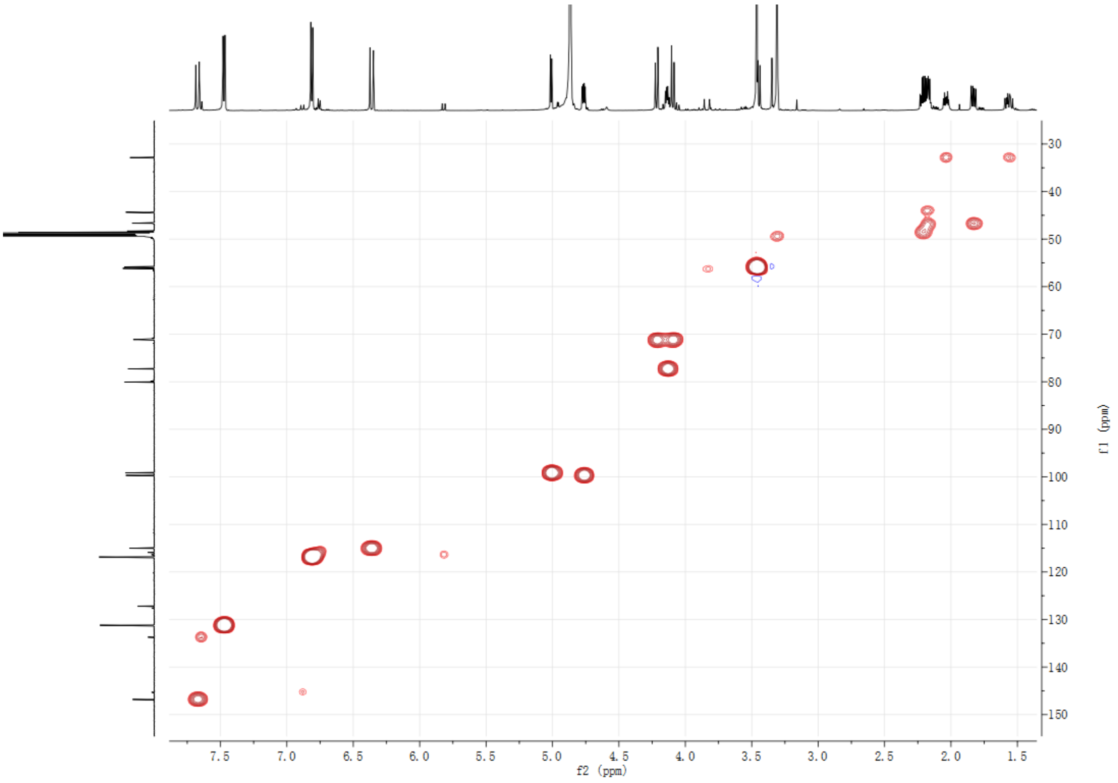

Figure S35. The HMBC spectrum of **5** (CD_3OD).

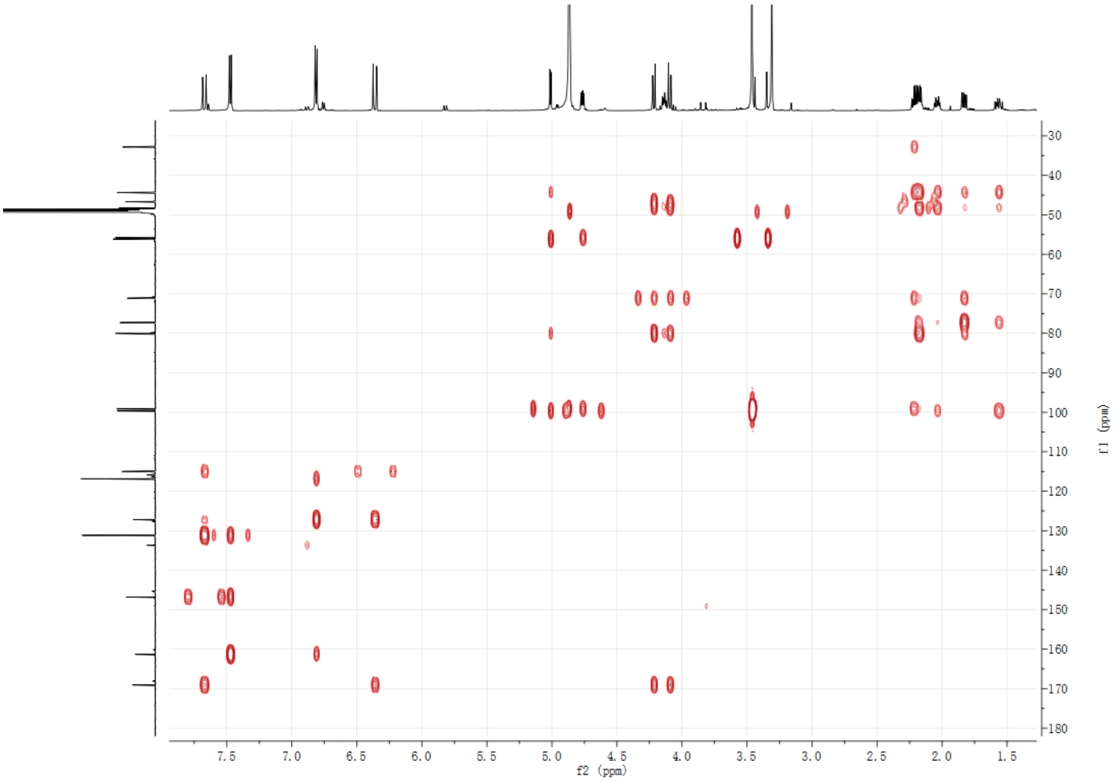

Figure S36. The NOESY spectrum of **5** (CD_3OD).

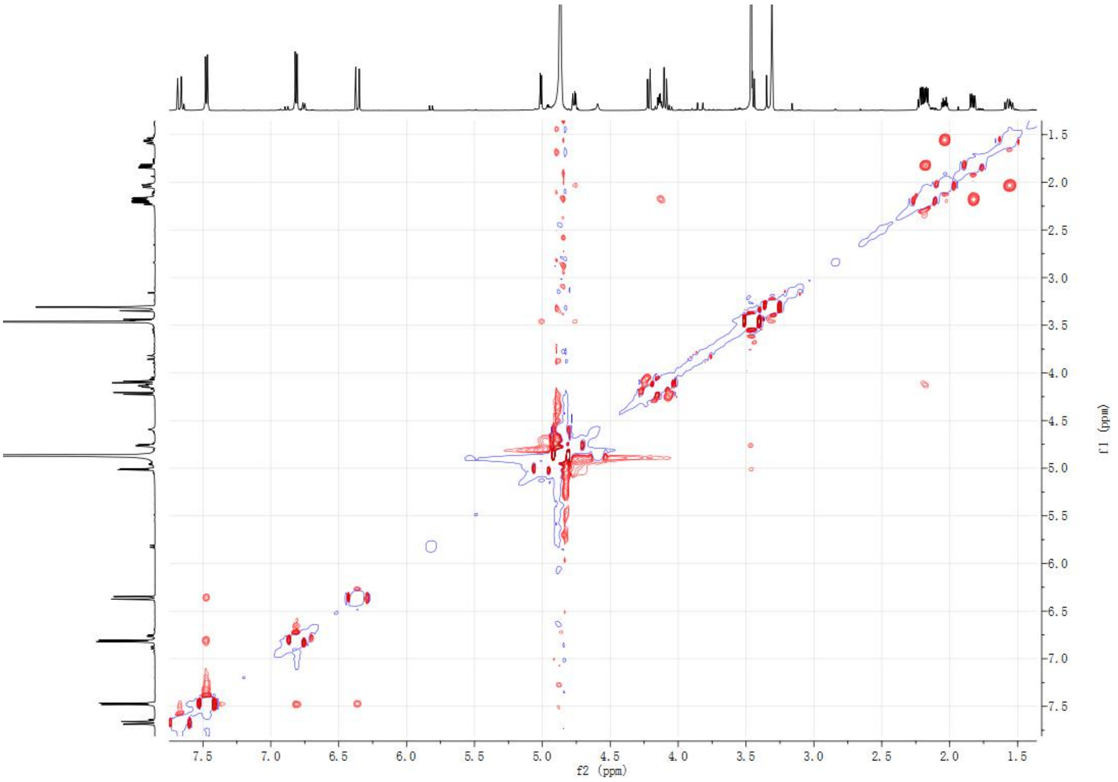

Figure S37. ^{1}H NMR spectra of (*S*) and (*R*)-MTPA esters of **5** (CD_3OD).

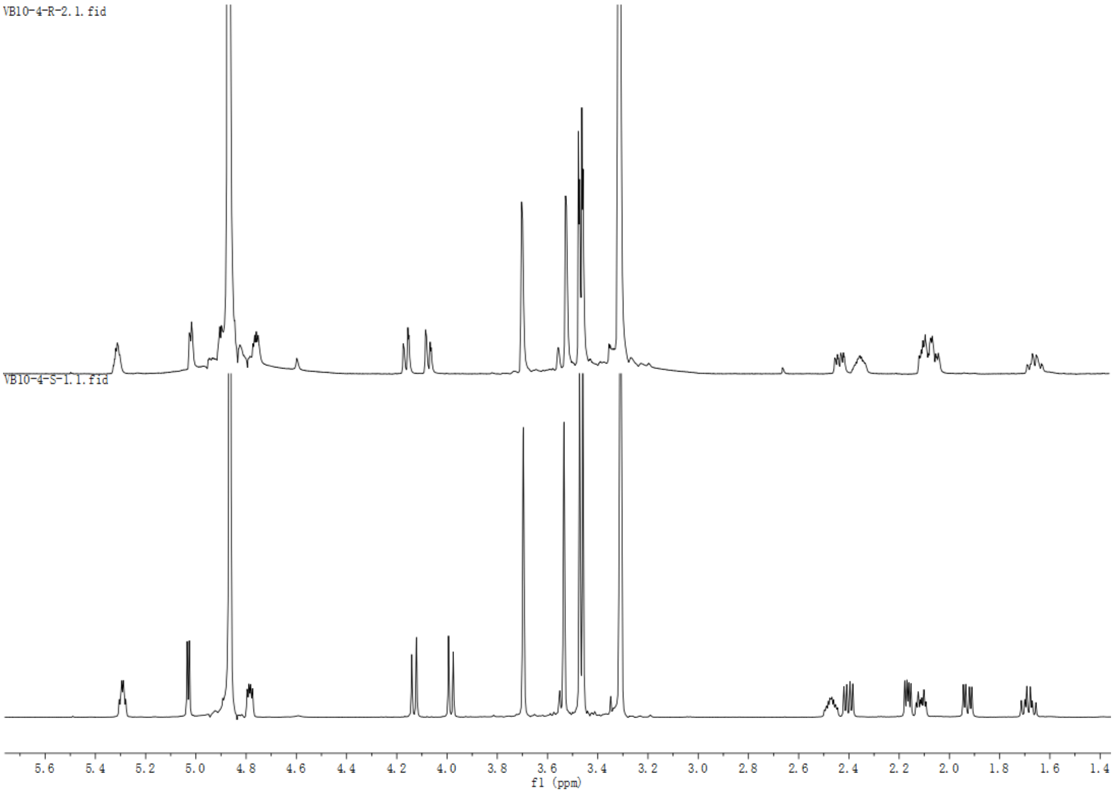

Figure S38. The (+)-HR-ESIMS spectrum of **5**.

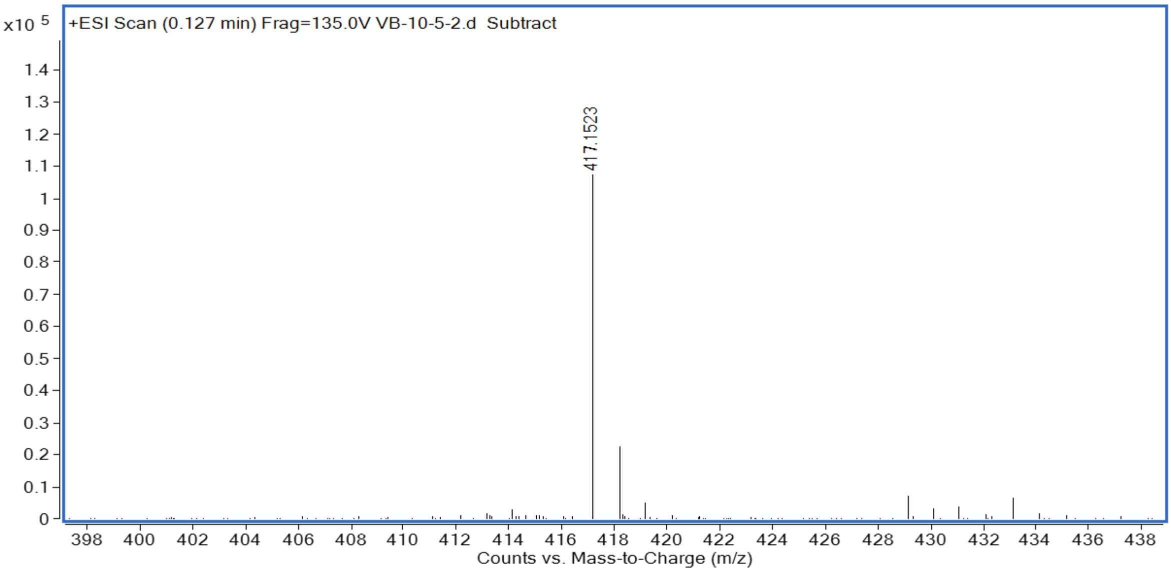

Figure S39. The ^{1}H NMR spectrum of **5r** ($CDCl_3$).

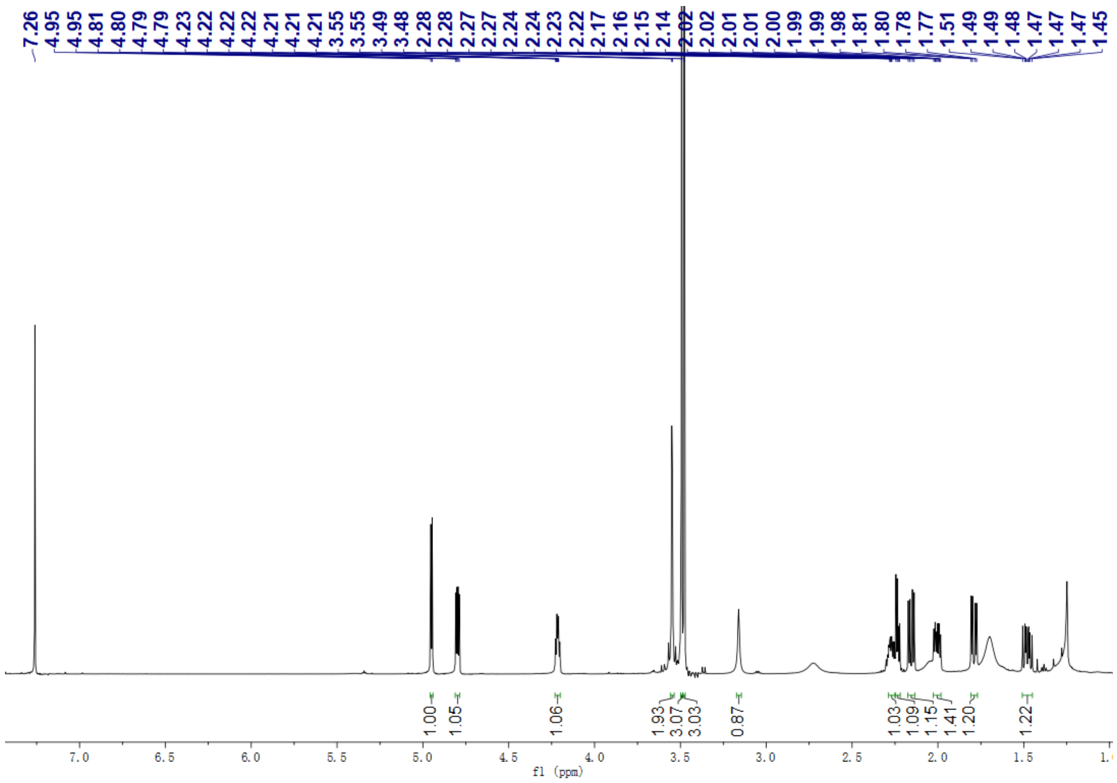

Figure S40. The ^{13}C and DEPT NMR spectra of **5r** ($CDCl_3$).

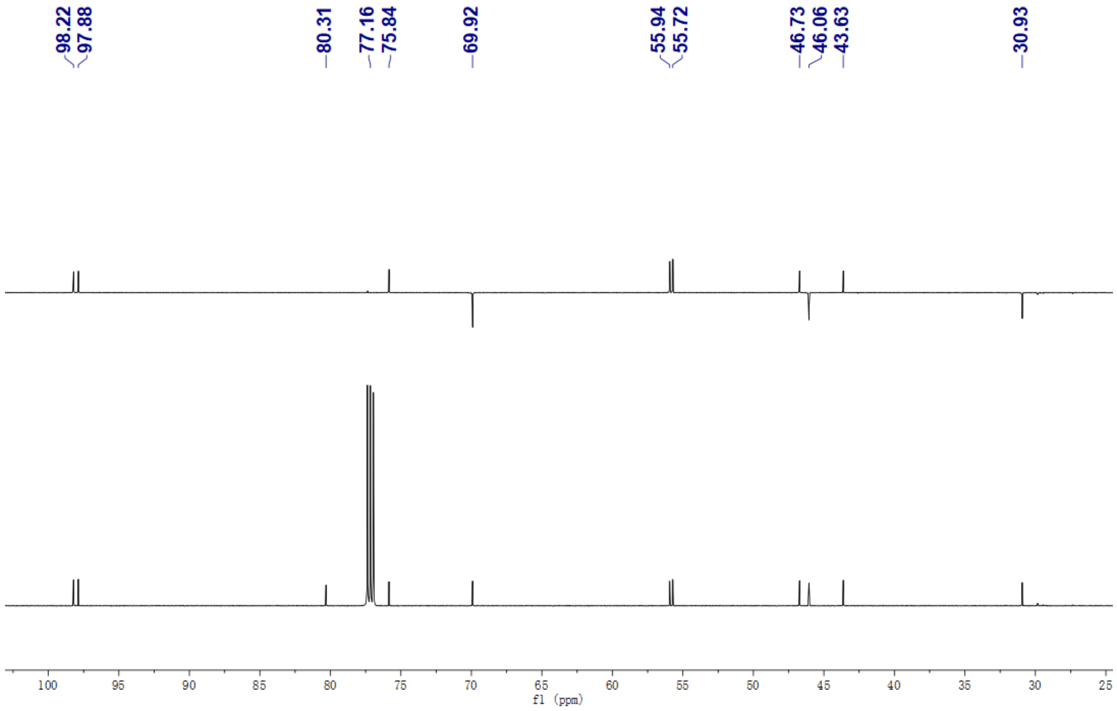

Figure S41. The ^{1}H-^{1}H COSY spectrum of **5r** ($CDCl_3$).

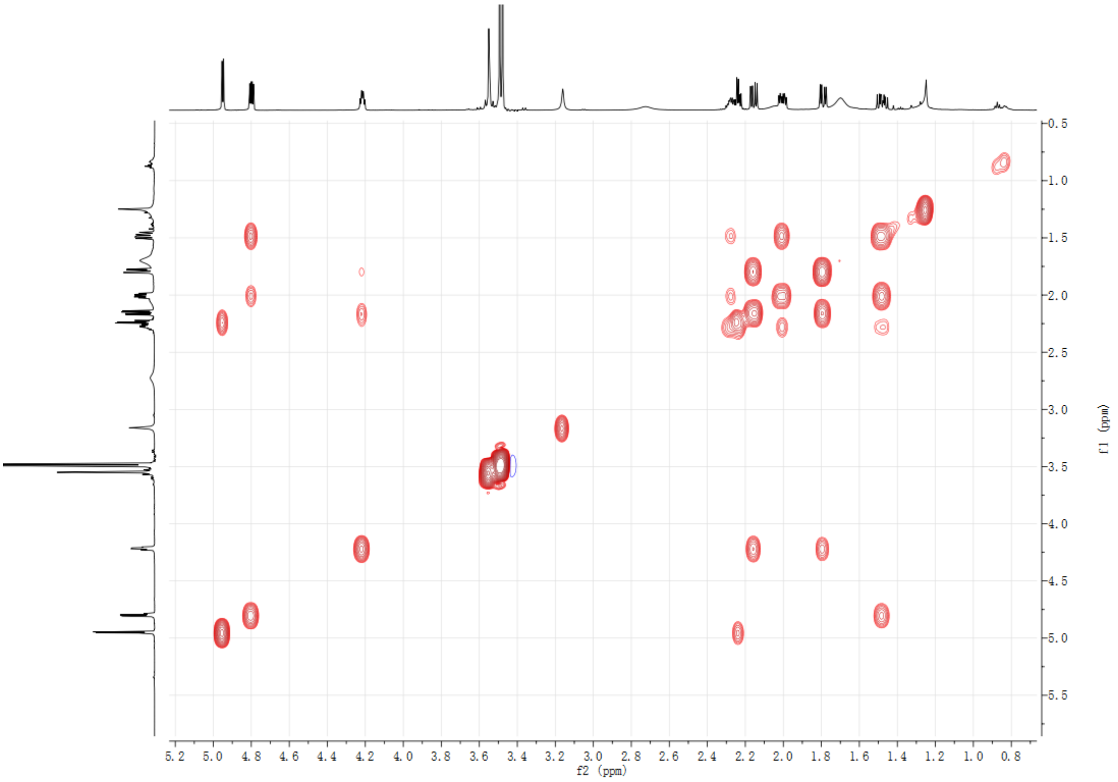

Figure S42. The HSQC spectrum of **5r** ($CDCl_3$).

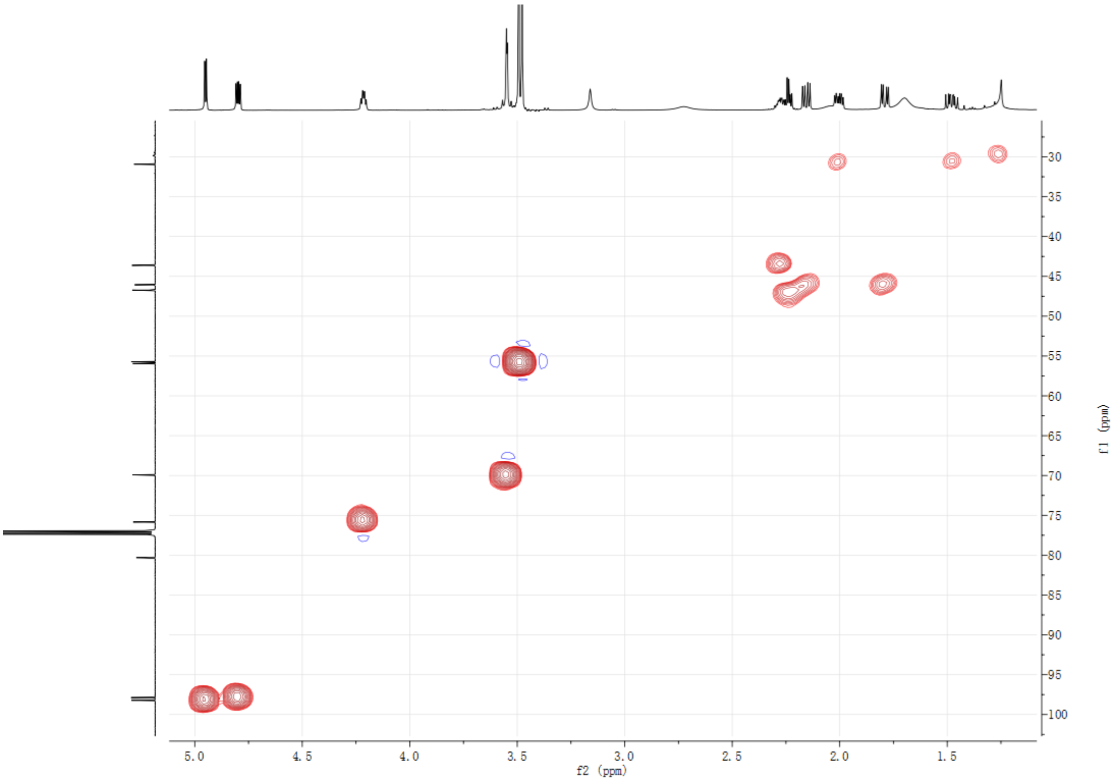

Figure S43. The HMBC spectrum of **5r** ($CDCl_3$).

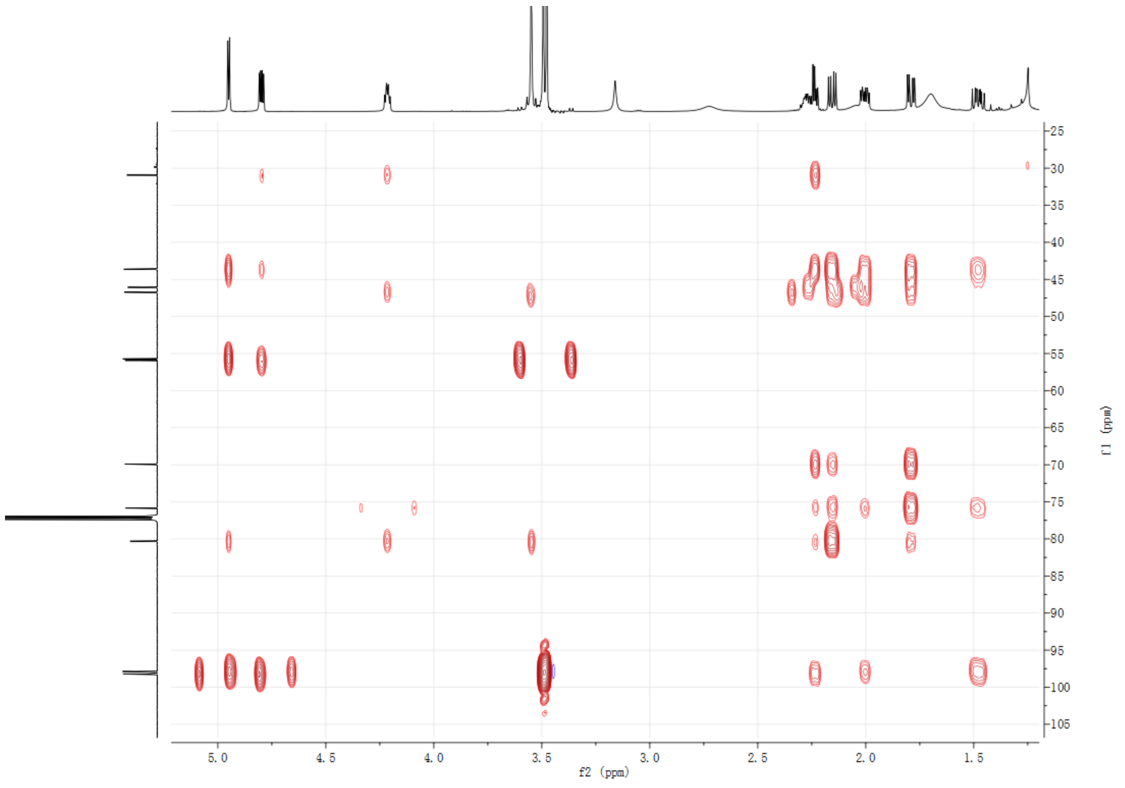

Figure S44. The NOESY spectrum of **5r** ($CDCl_3$).

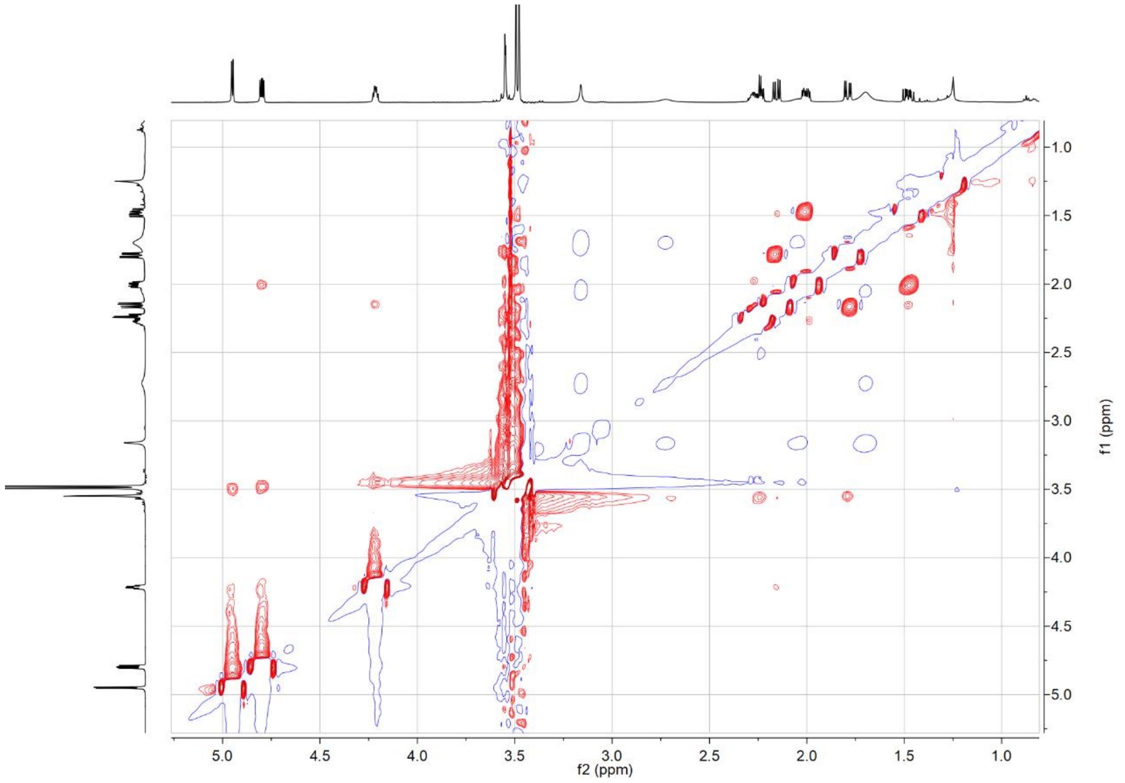

Figure S45. The 1D-NOE spectrum of **5r** ($CDCl_3$, H-4b).

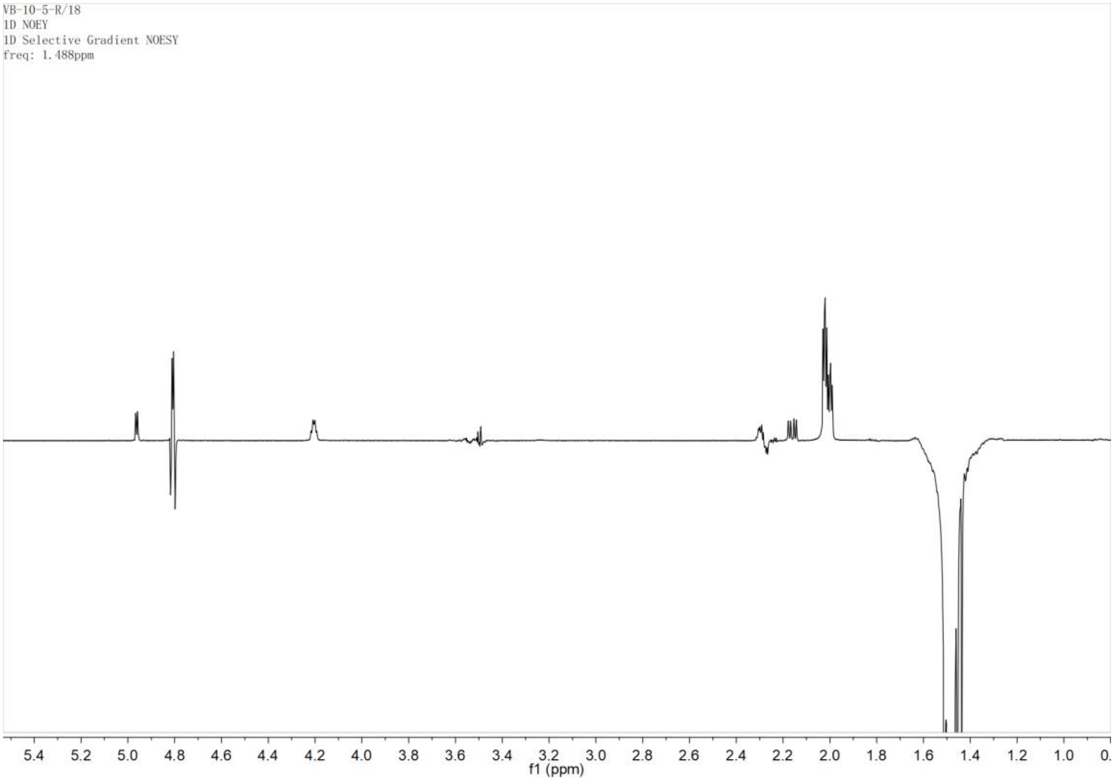

Figure S46. The ^{1}H NMR spectrum of **5r** (CD_3OD).

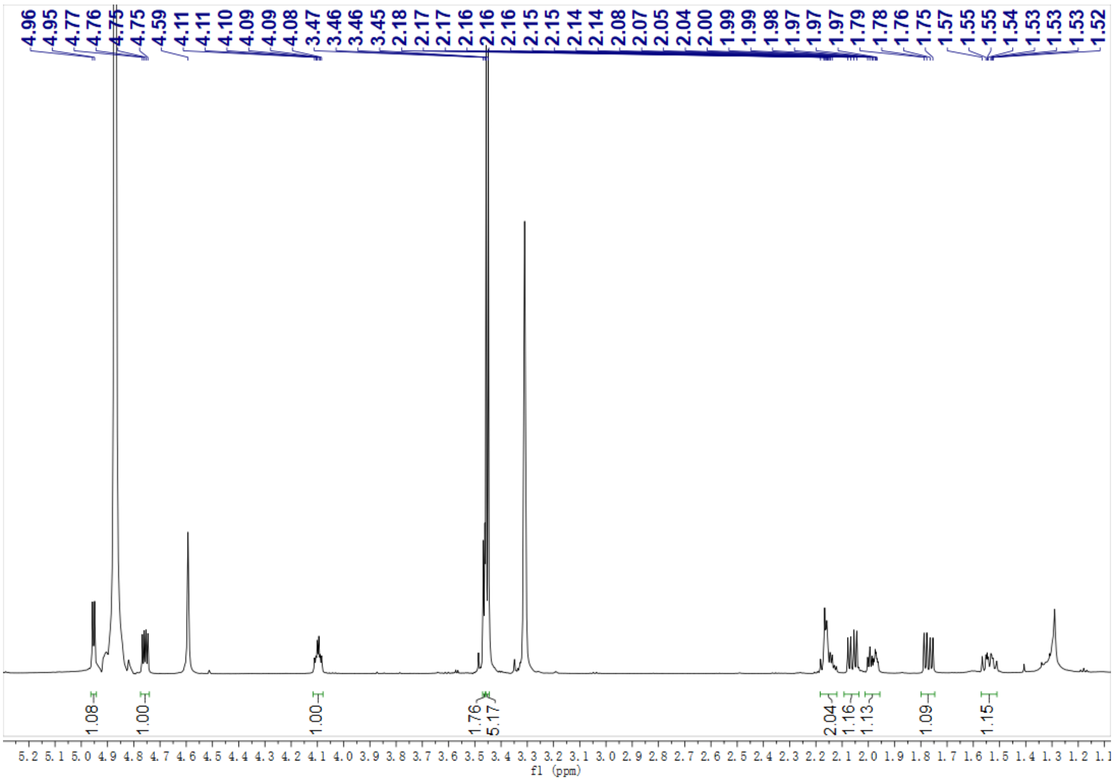

Figure S47. The ^{13}C and DEPT NMR spectra of **5r** (CD_3OD).

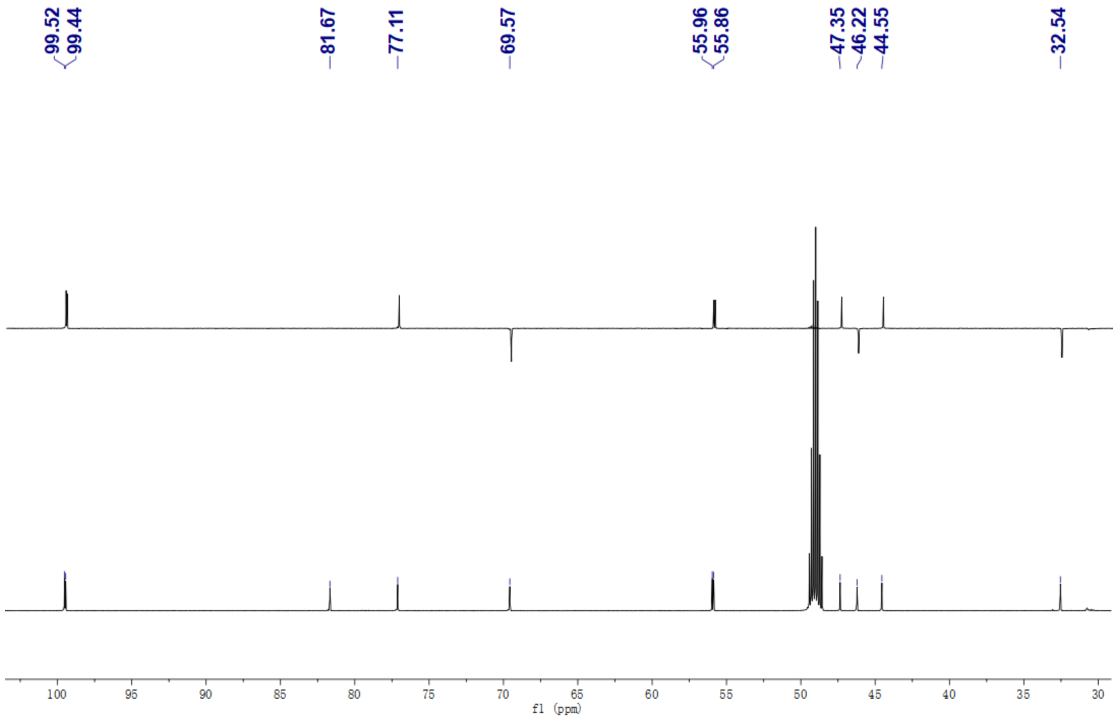

Figure S48. The ^{1}H NMR spectrum of **6** (CD_3OD).

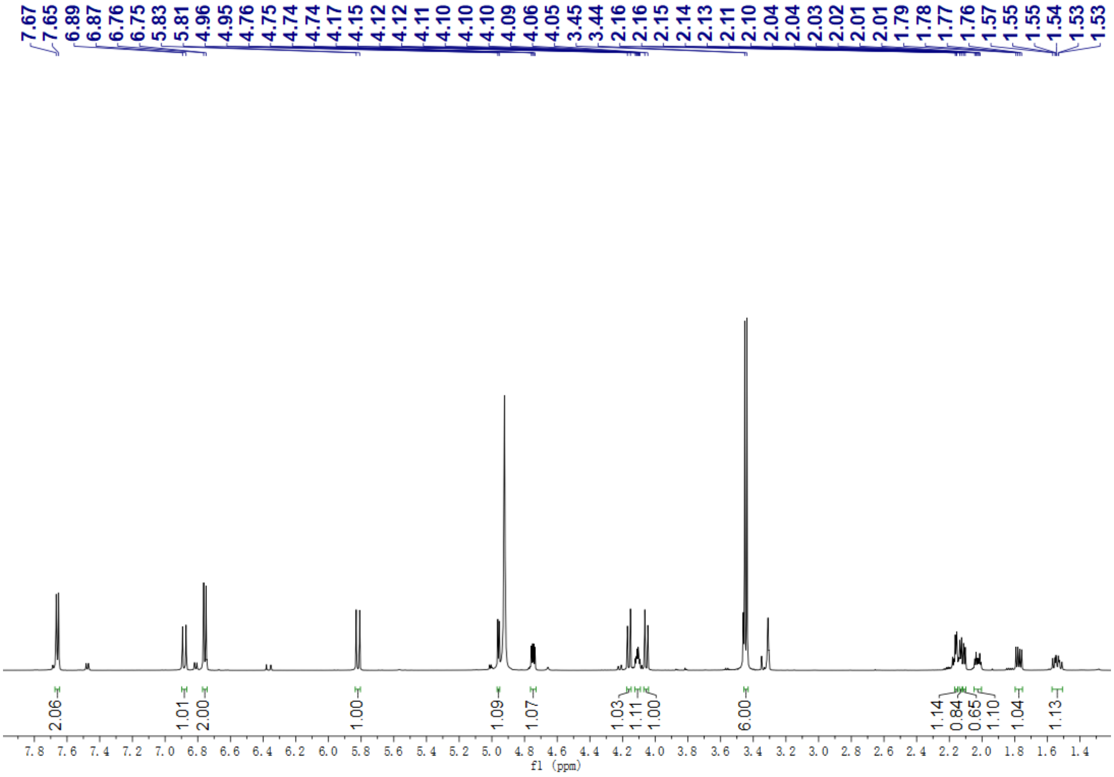

Figure S49. The ^{13}C and DEPT NMR spectra of **6** (CD_3OD).

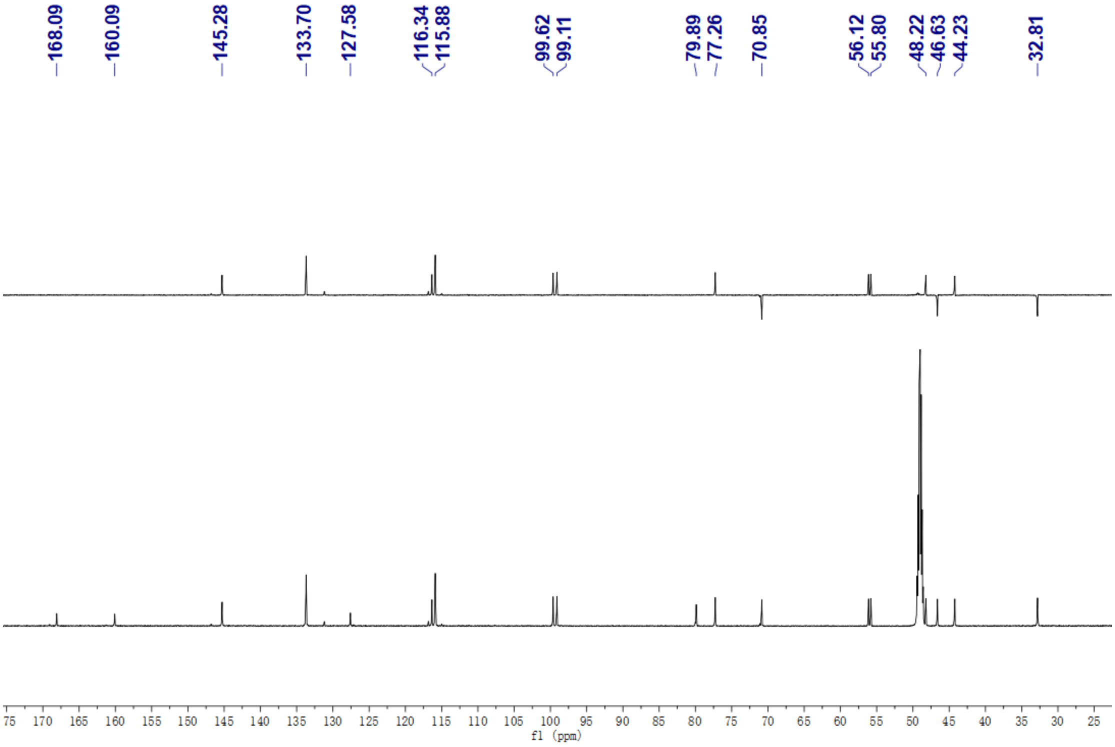

Figure S50. The ^{1}H-^{1}H COSY spectrum of **6** (CD_3OD).

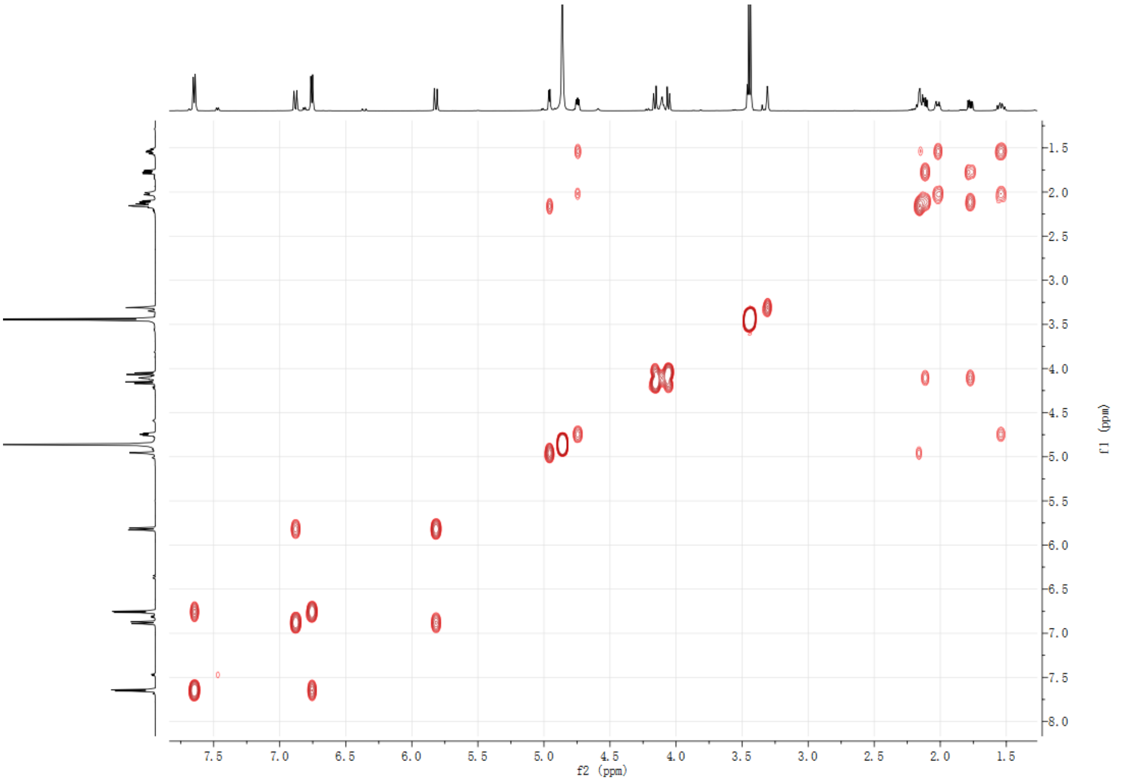

Figure S51. The HSQC spectrum of **6** (CD_3OD).

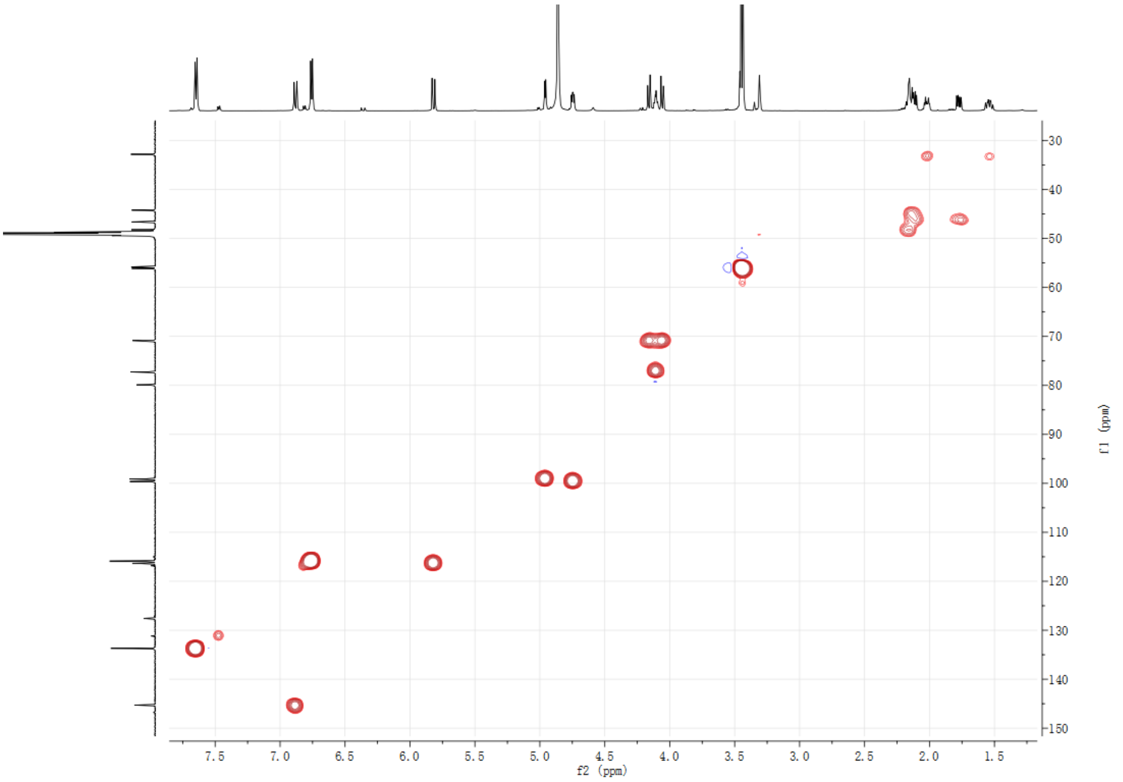

Figure S52. The HMBC spectrum of **6** (CD_3OD).

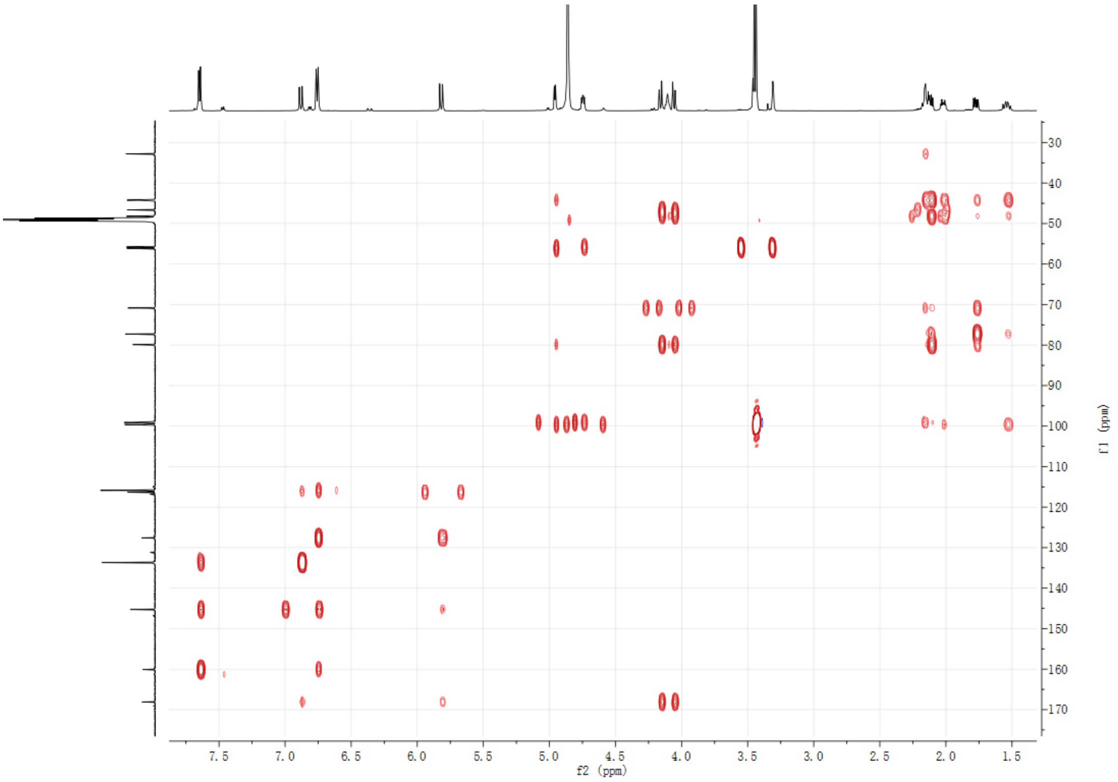

Figure S53. The NOESY spectrum of **6** (CD_3OD).

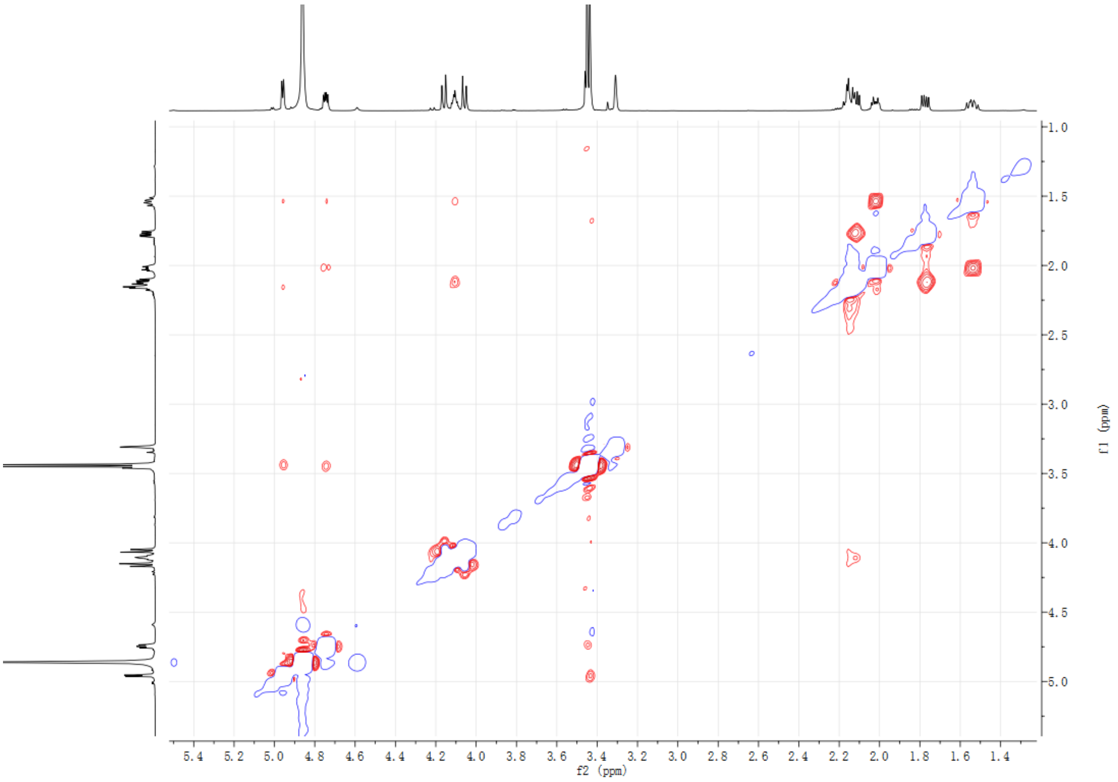

Figure S54. The (+)-HR-ESIMS spectrum of **6**.

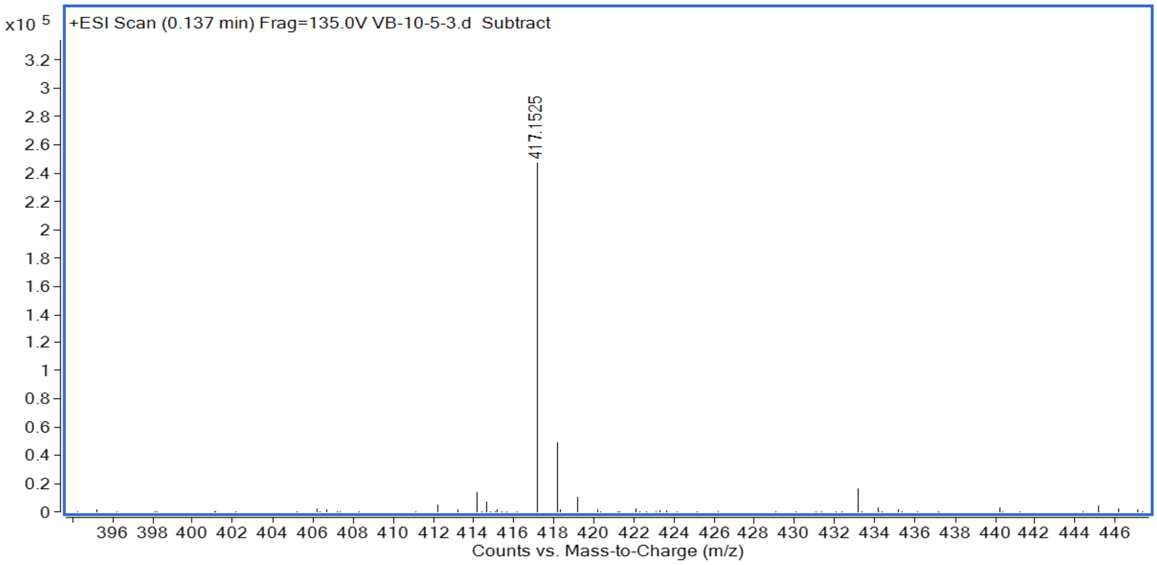

Figure S55. The ^{1}H NMR spectrum of **7** (CD_3OD).

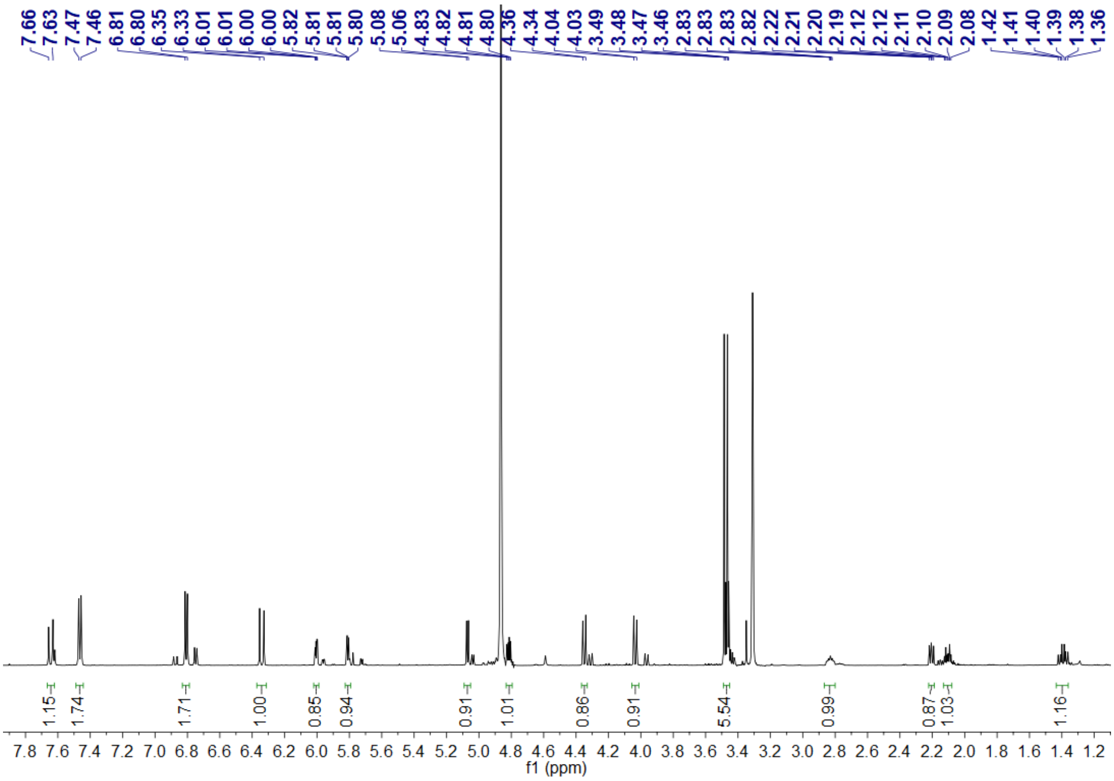

Figure S56. The ^{13}C and DEPT NMR spectra of **7** (CD_3OD).

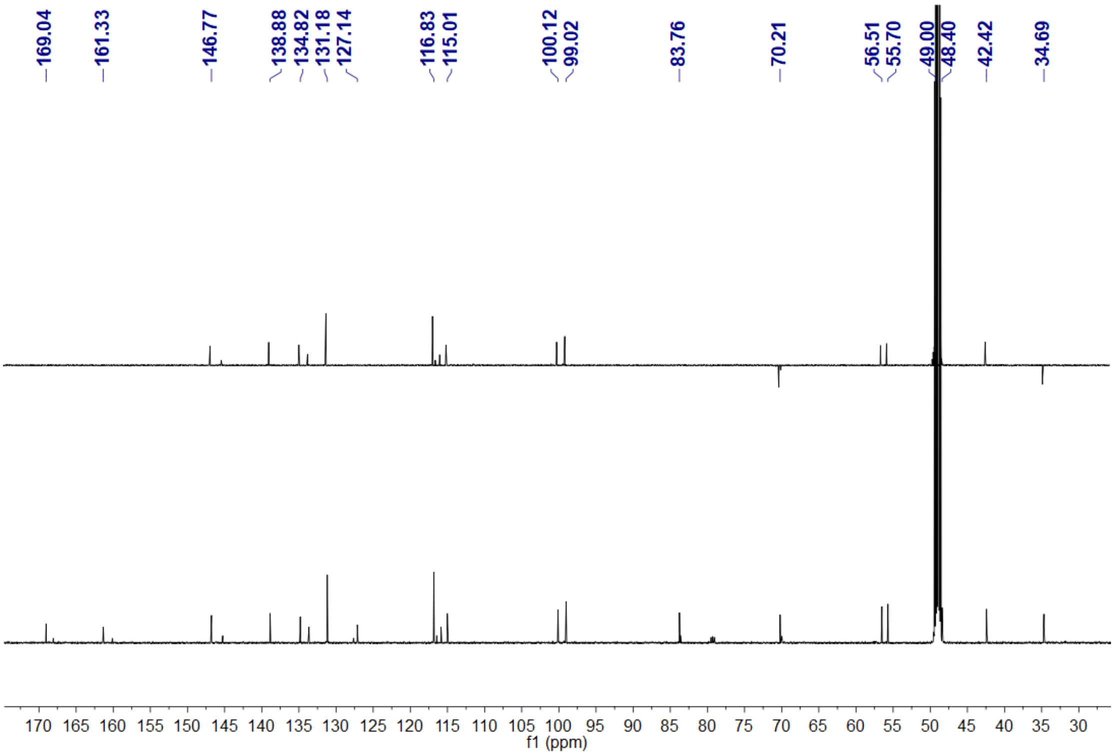

Figure S57. The ^{1}H-^{1}H COSY spectrum of **7** (CD_3OD).

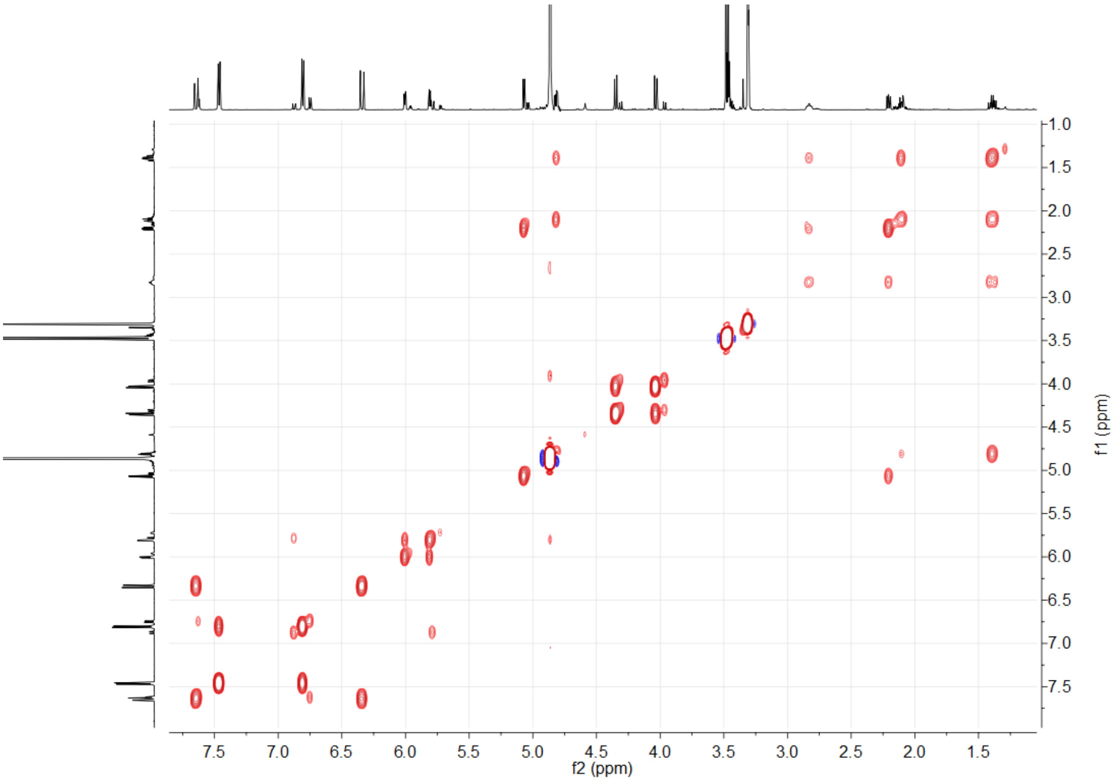

Figure S58. The HSQC spectrum of **7** (CD_3OD).

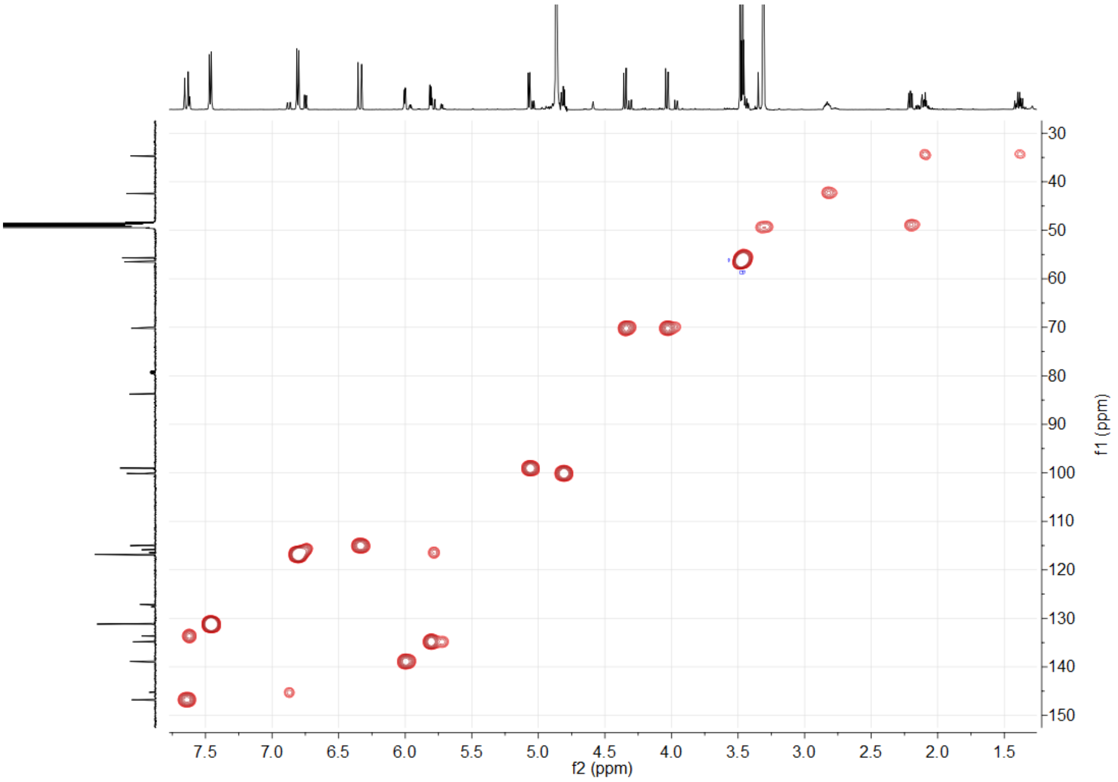

Figure S59. The HMBC spectrum of **7** (CD_3OD).

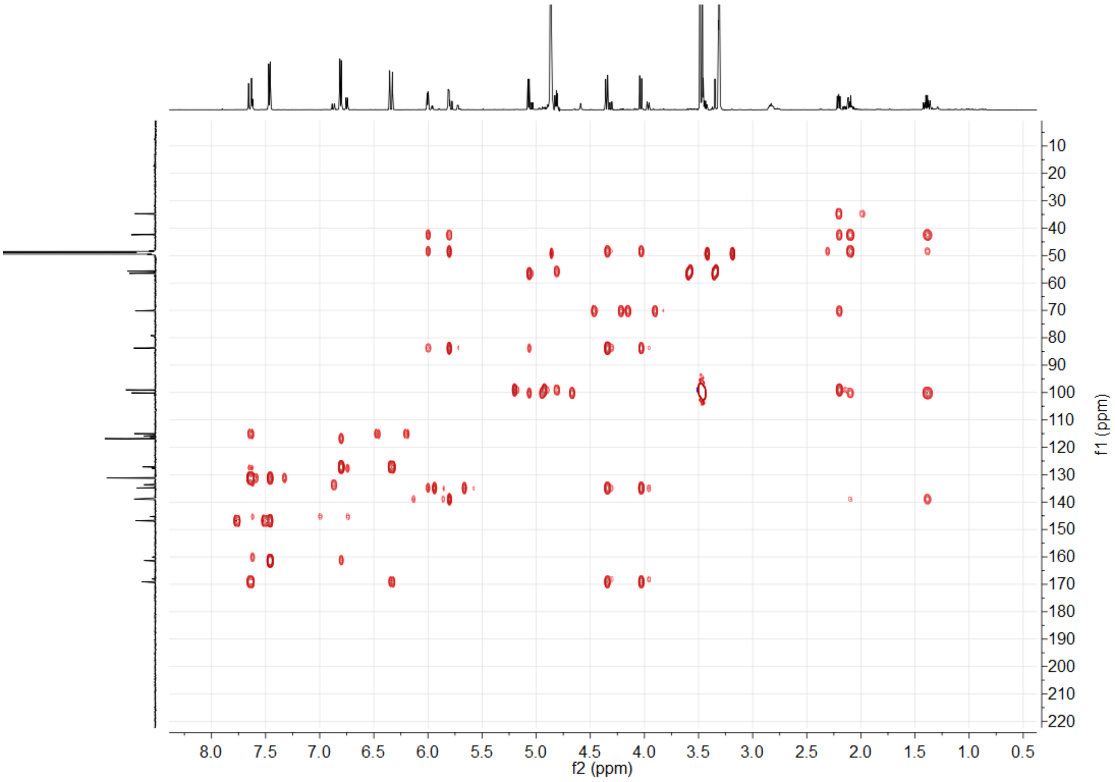

Figure S60. The NOESY spectrum of **7** (CD_3OD).

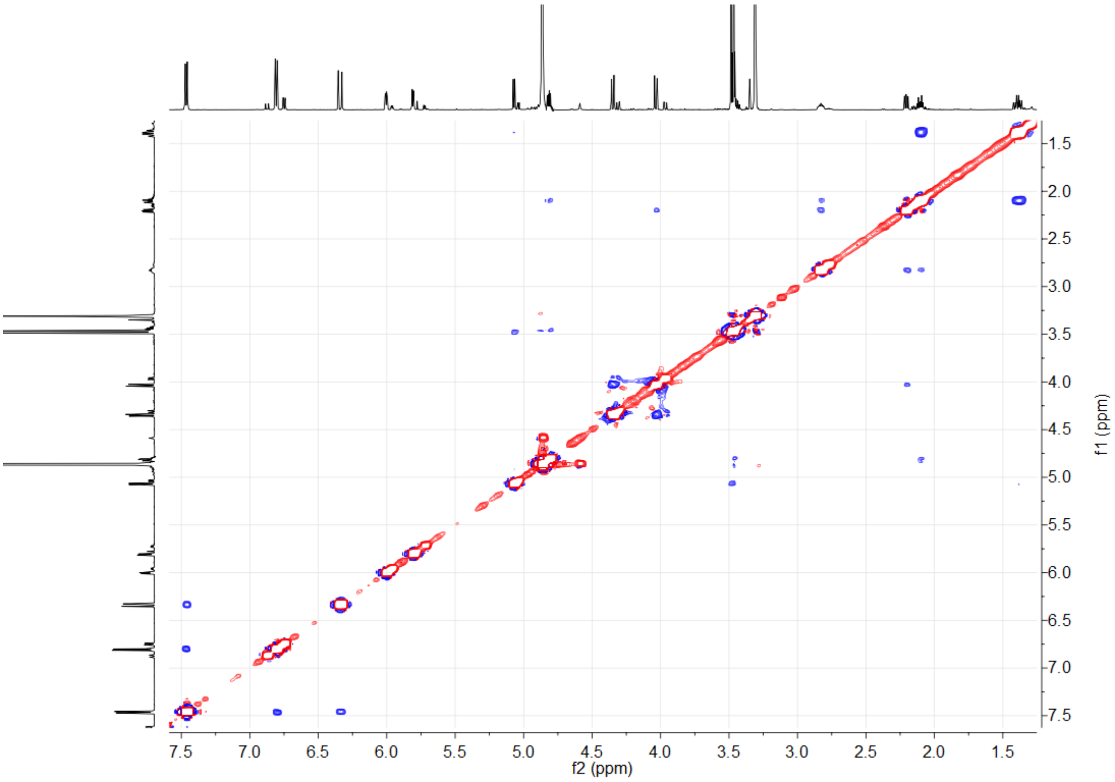

Figure S61. The ^{1}H NMR spectrum of **7** ($CDCl_3$).

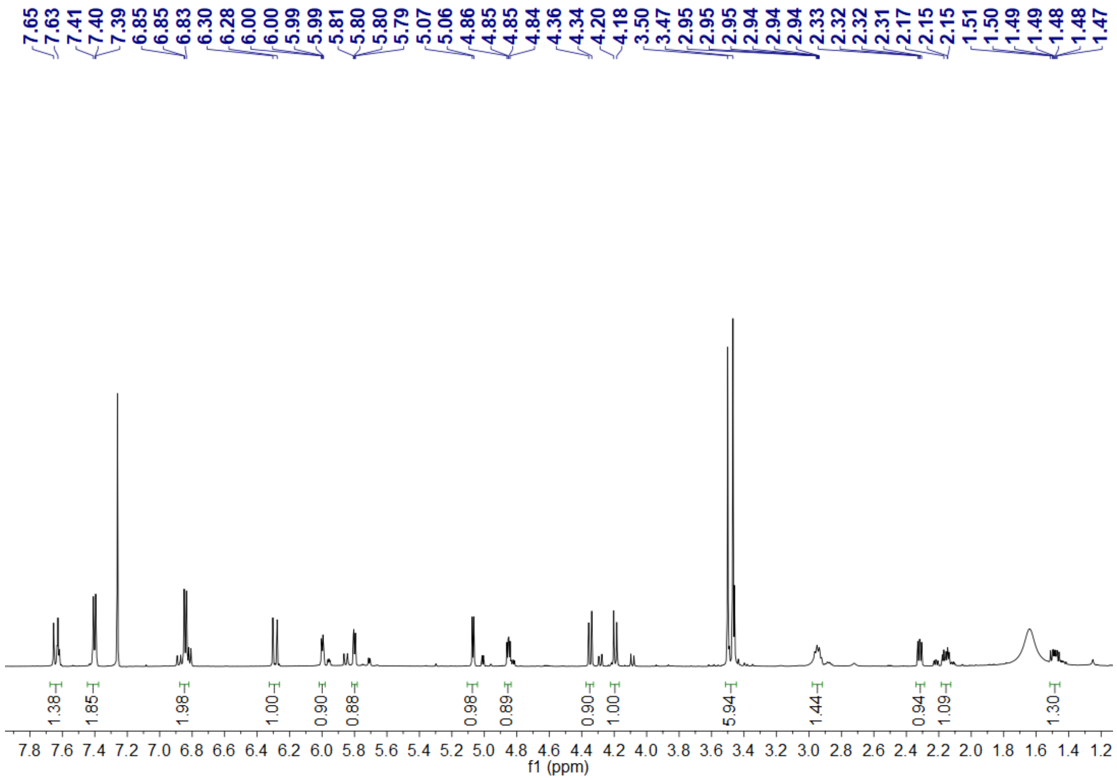

Figure S62. The ^{13}C and DEPT NMR spectra of **7** ($CDCl_3$).

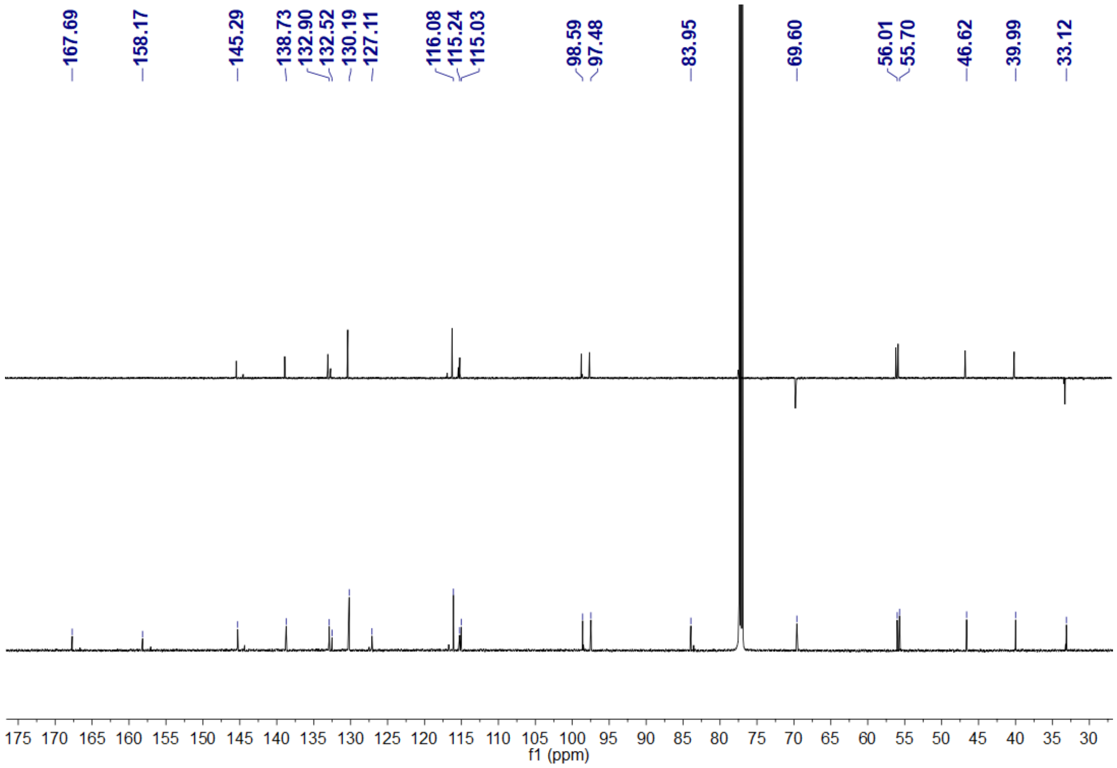

Figure S63. The (+)-HR-ESIMS spectrum of **7**.

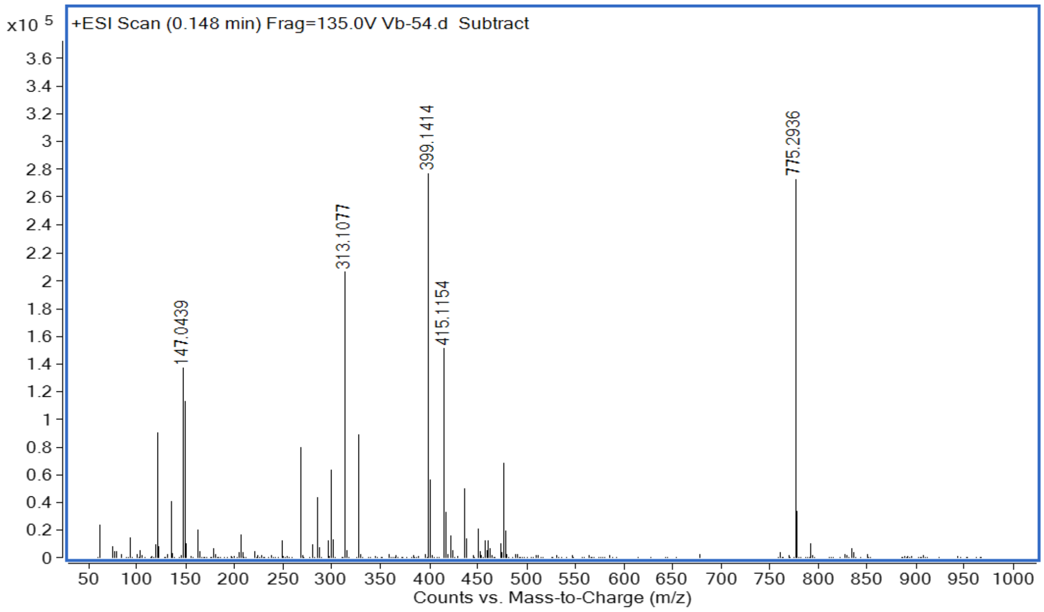

Figure S64. The ^{1}H NMR spectrum of **8** (CD_3OD).

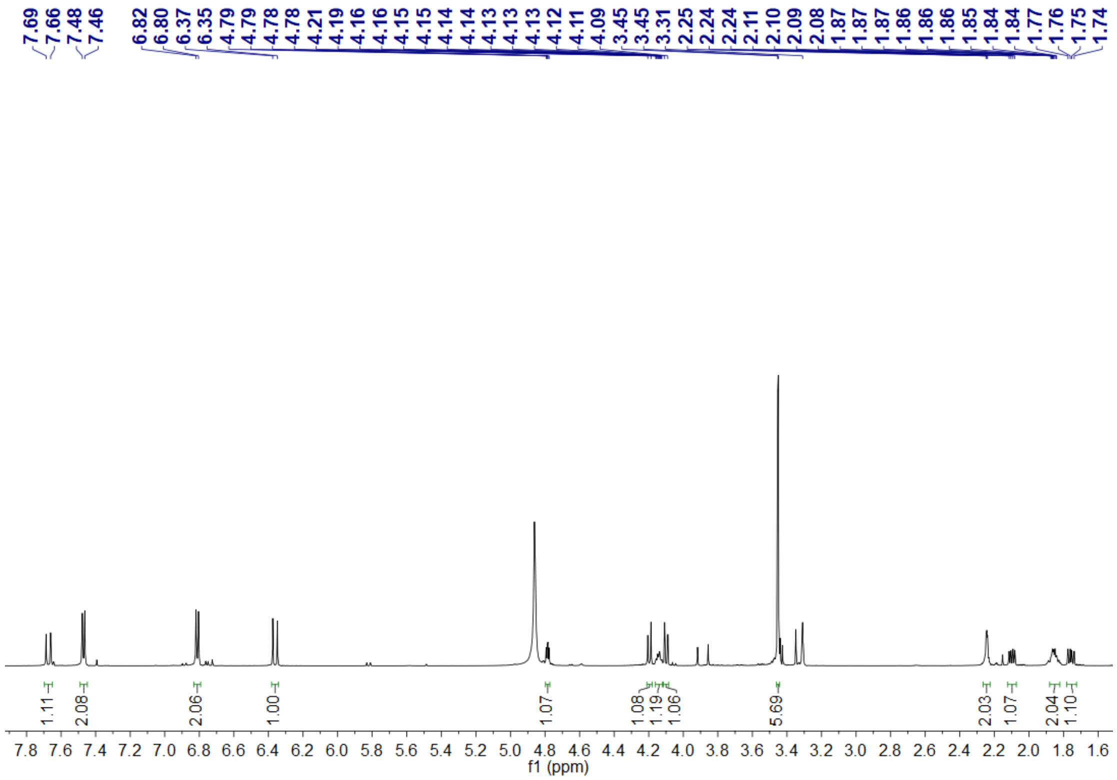

Figure S65. The ^{13}C and DEPT NMR spectra of **8** (CD_3OD).

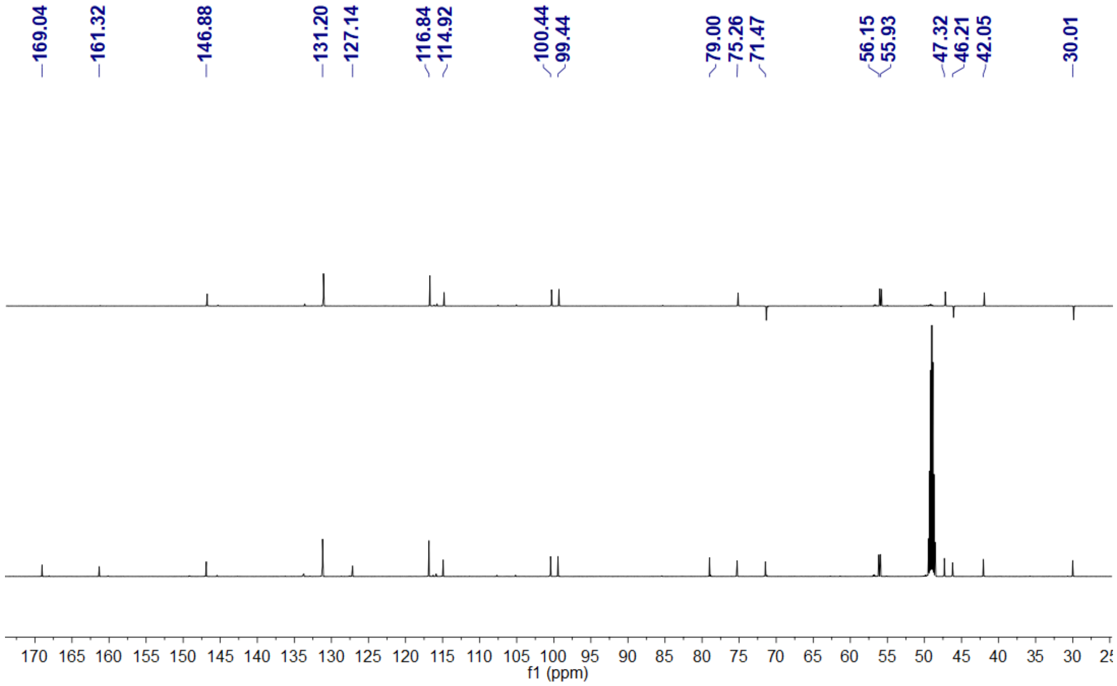

Figure S66. The ^{1}H-^{1}H COSY spectrum of **8** (CD_3OD).

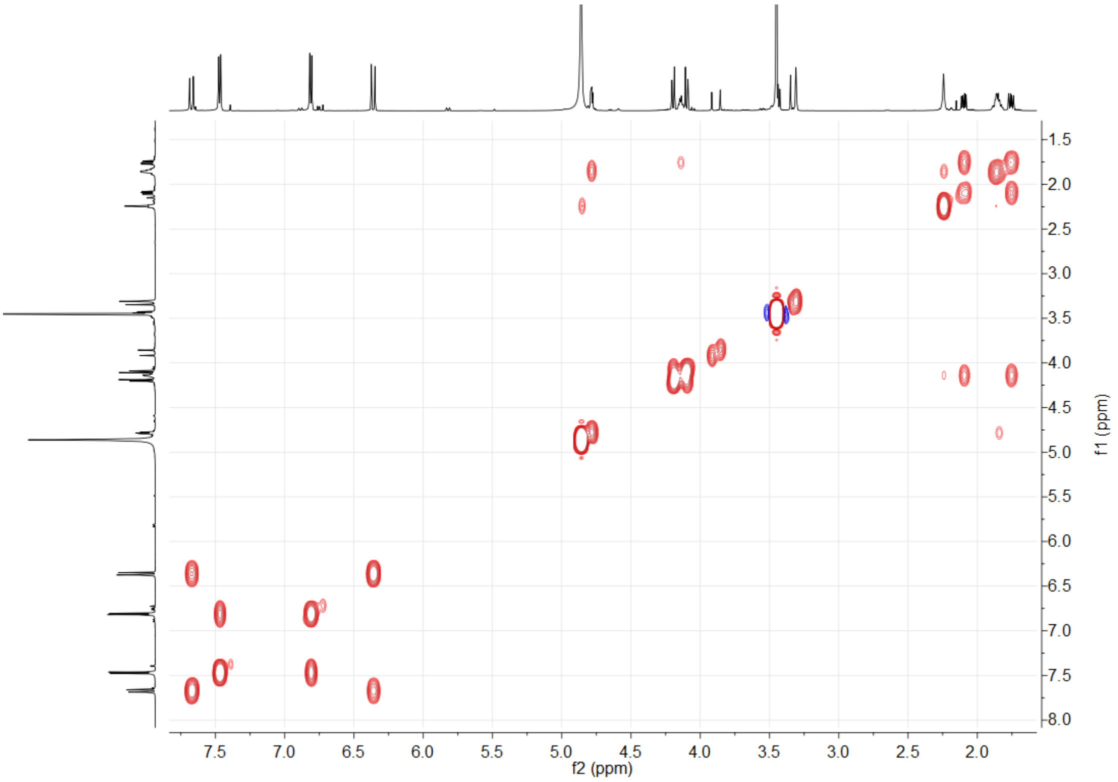

Figure S67. The HSQC spectrum of **8** (CD_3OD).

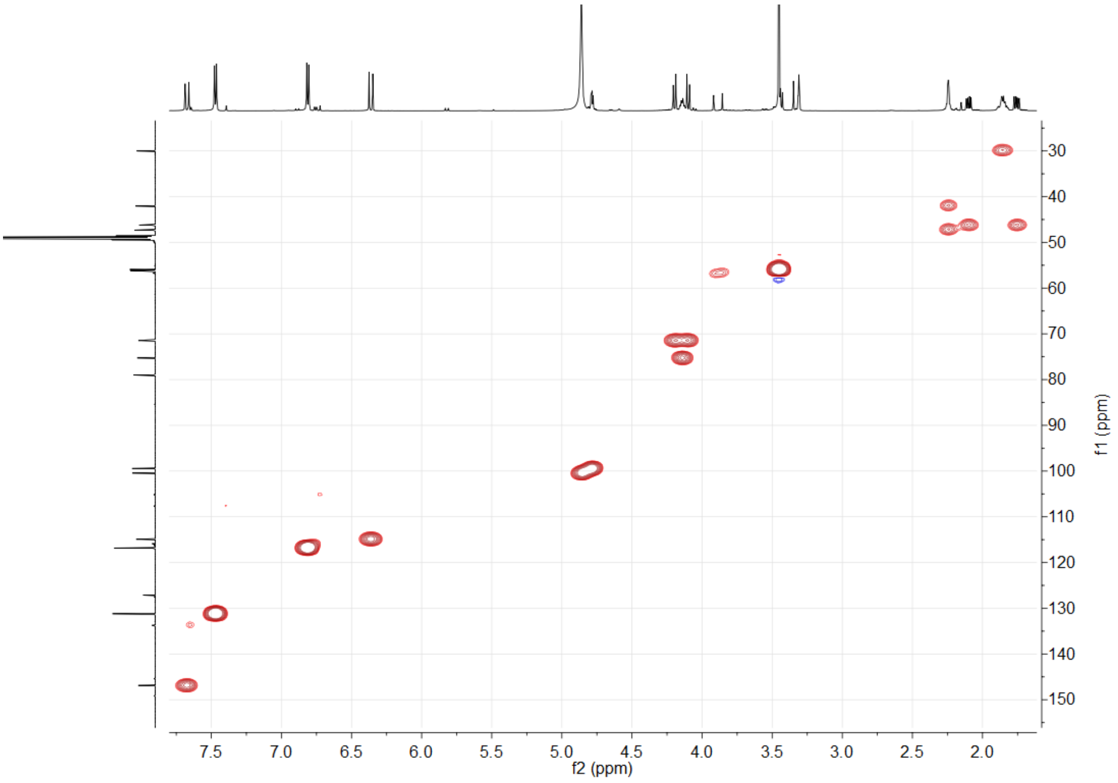

Figure S68. The HMBC spectrum of **8** (CD_3OD).

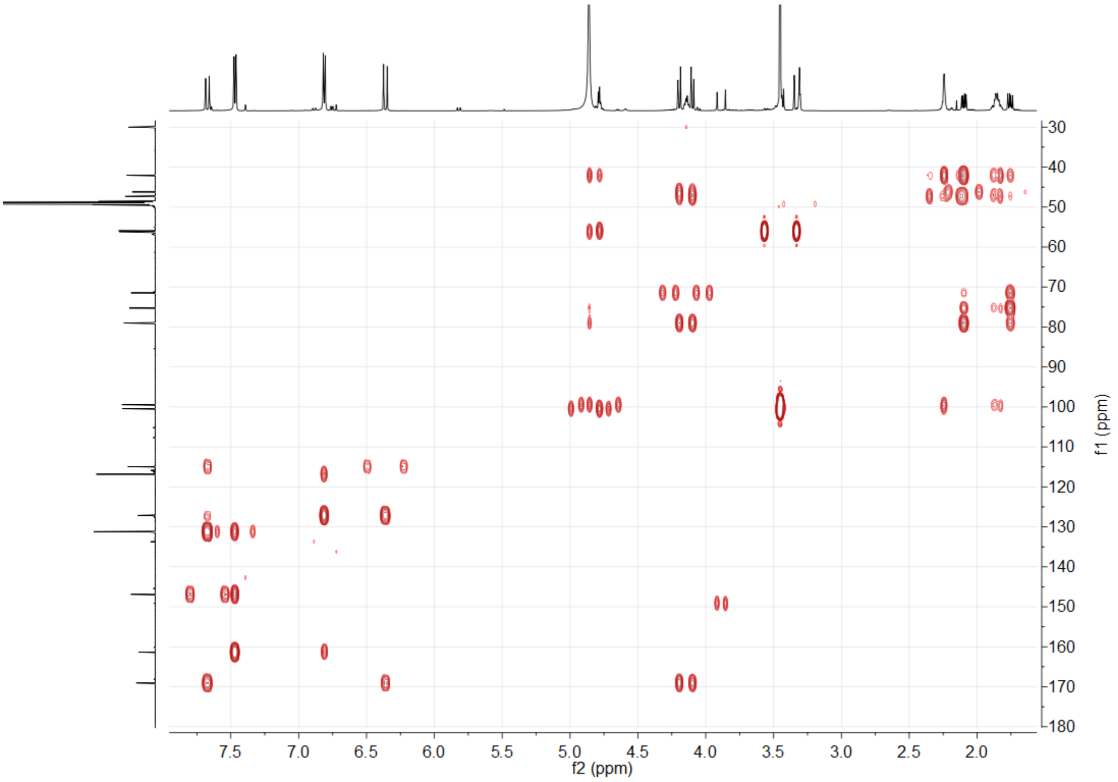

Figure S69. The NOESY spectrum of **8** (CD_3OD).

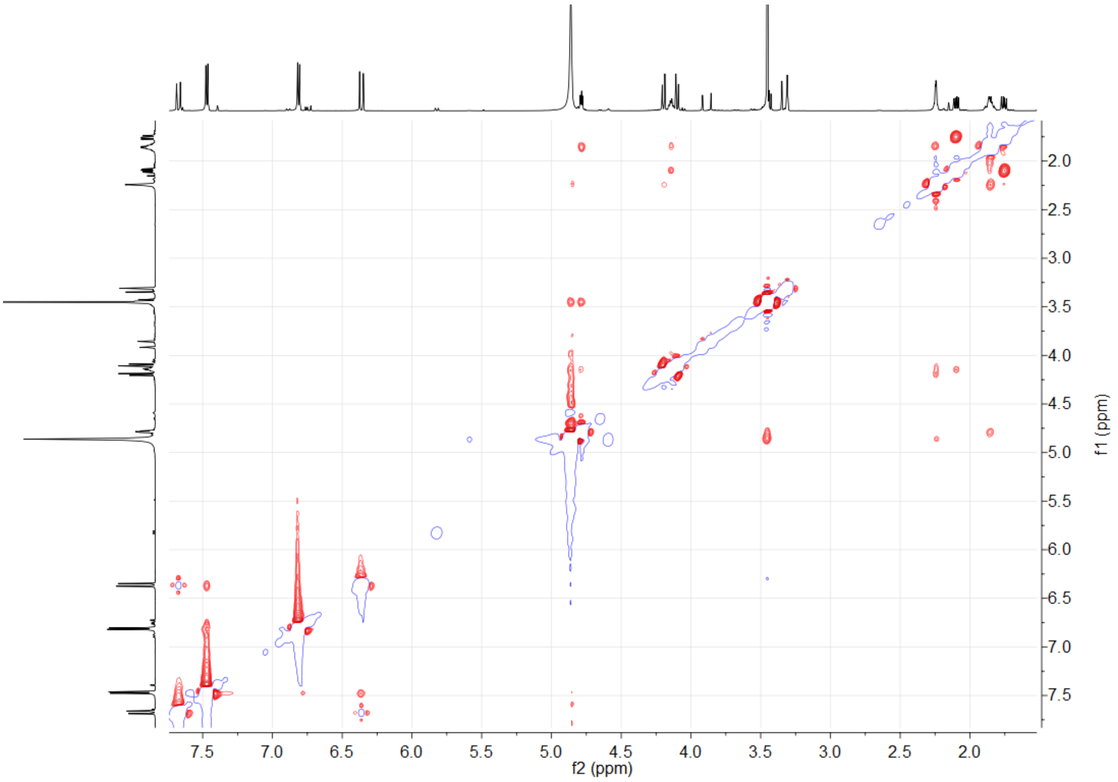

Figure S70. The ^{1}H NMR spectrum of **8** (DMSO-d_6).

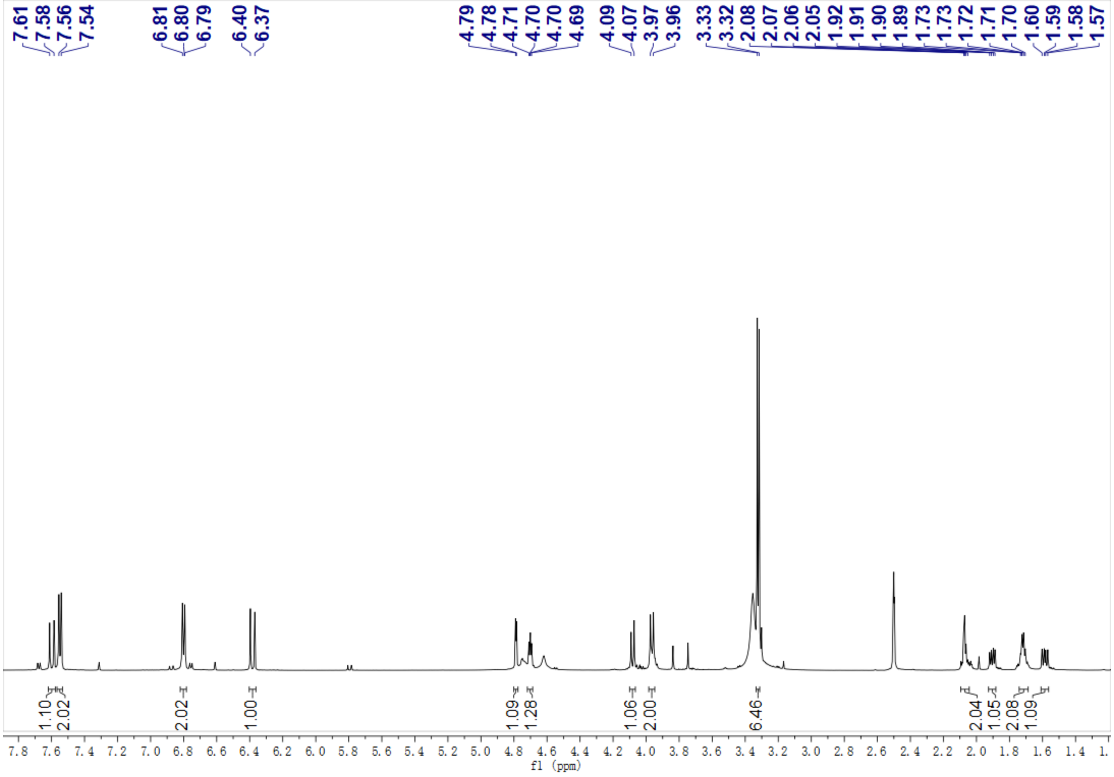

Figure S71. The ^{13}C and DEPT NMR spectra of **8** (DMSO-d_6).

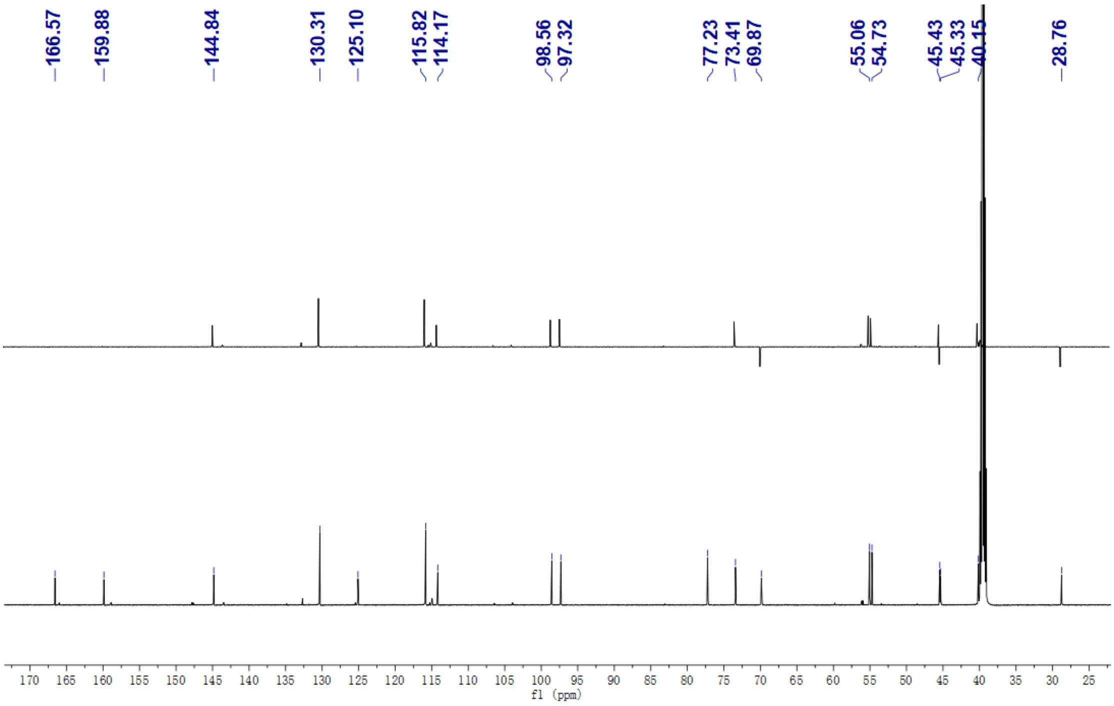

Figure S72. ^{1}H NMR spectra of (*S*) and (*R*)-MTPA esters of **8** (CD_3OD).

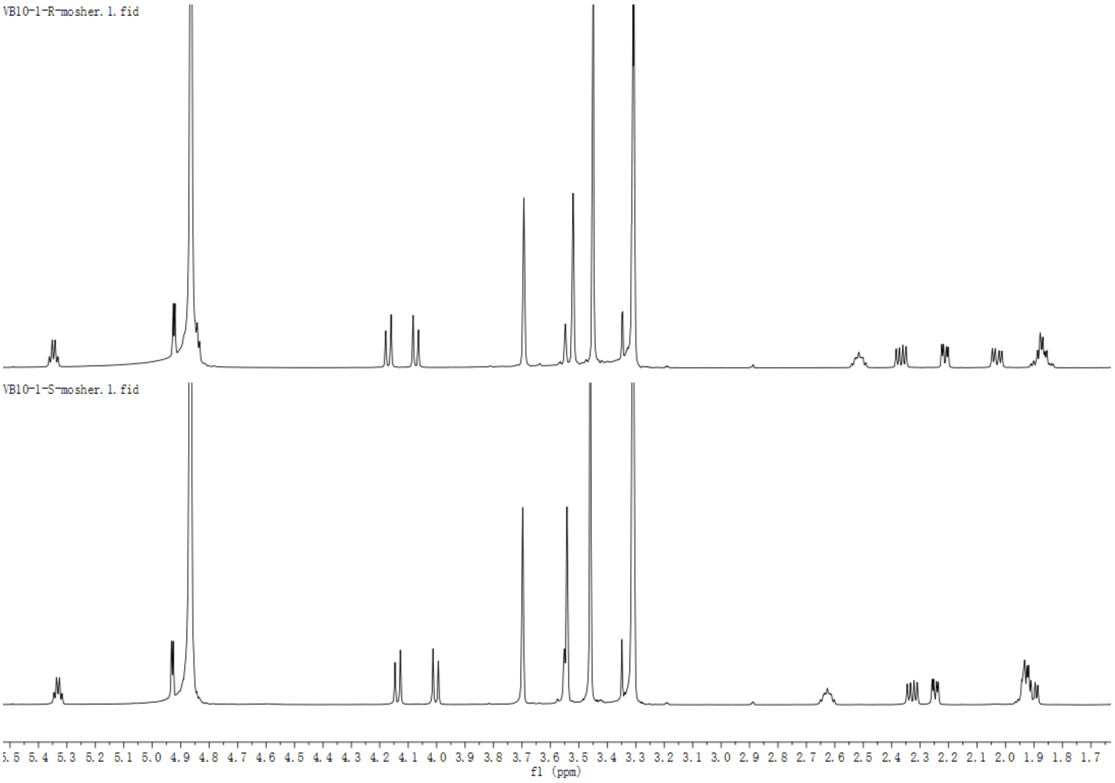

Figure S73. The (+)-HR-ESIMS spectrum of **8**.

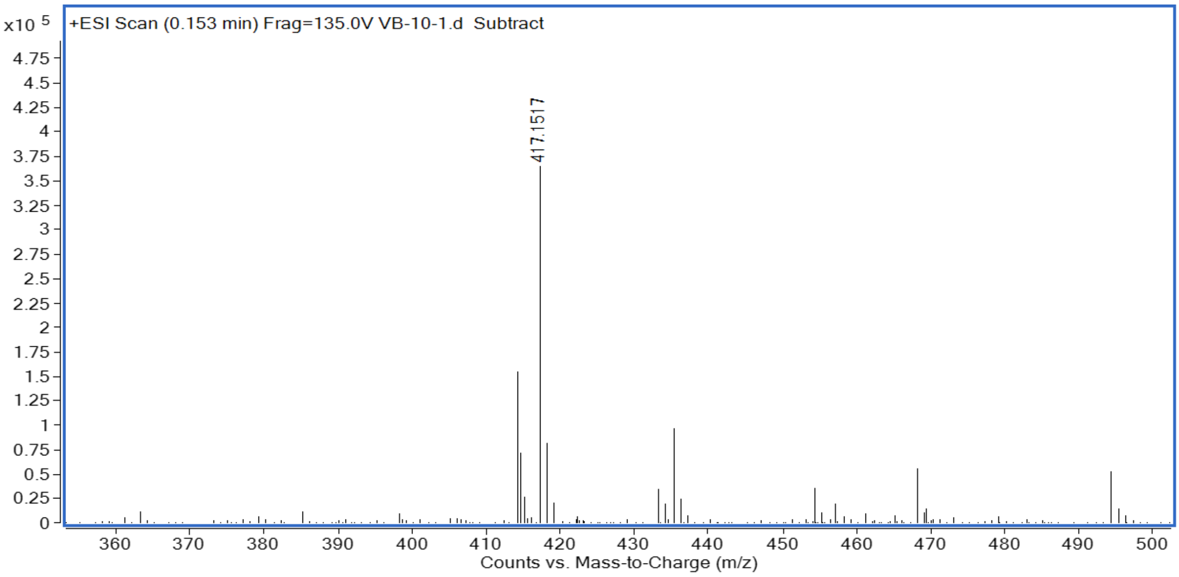

Figure S74. The ^{1}H NMR spectrum of **8r** ($CDCl_3$).

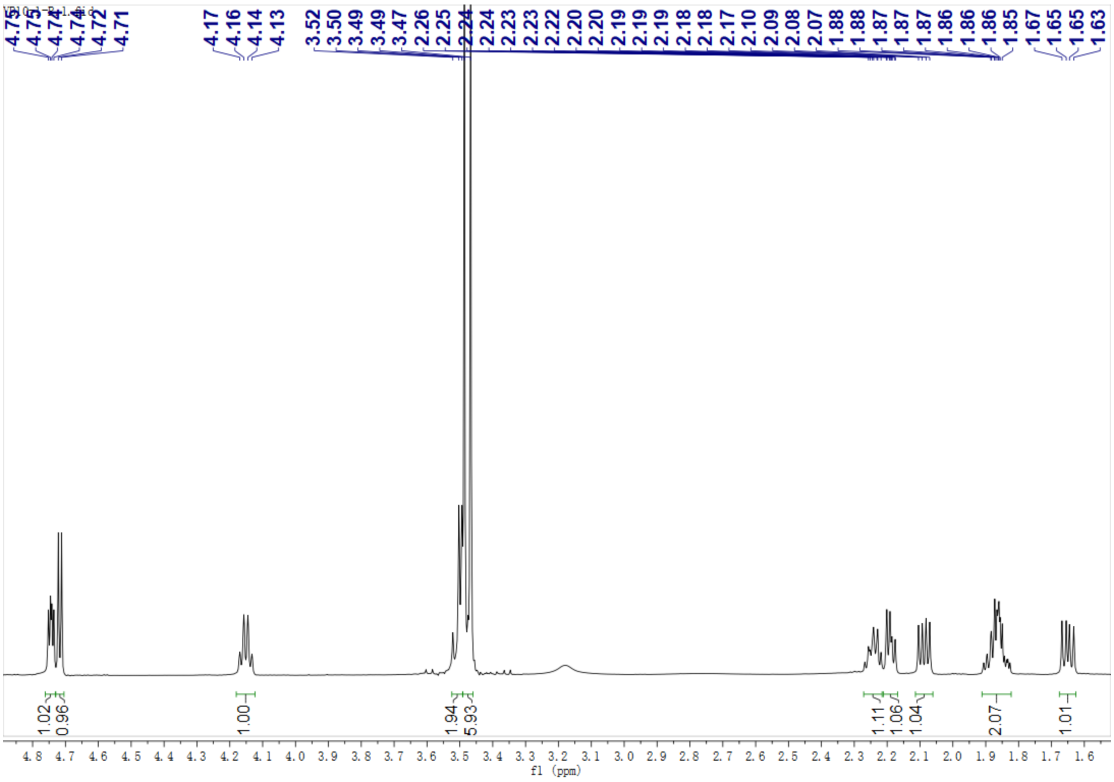

Figure S75. The ^{13}C and DEPT NMR spectra of **8r** ($CDCl_3$).

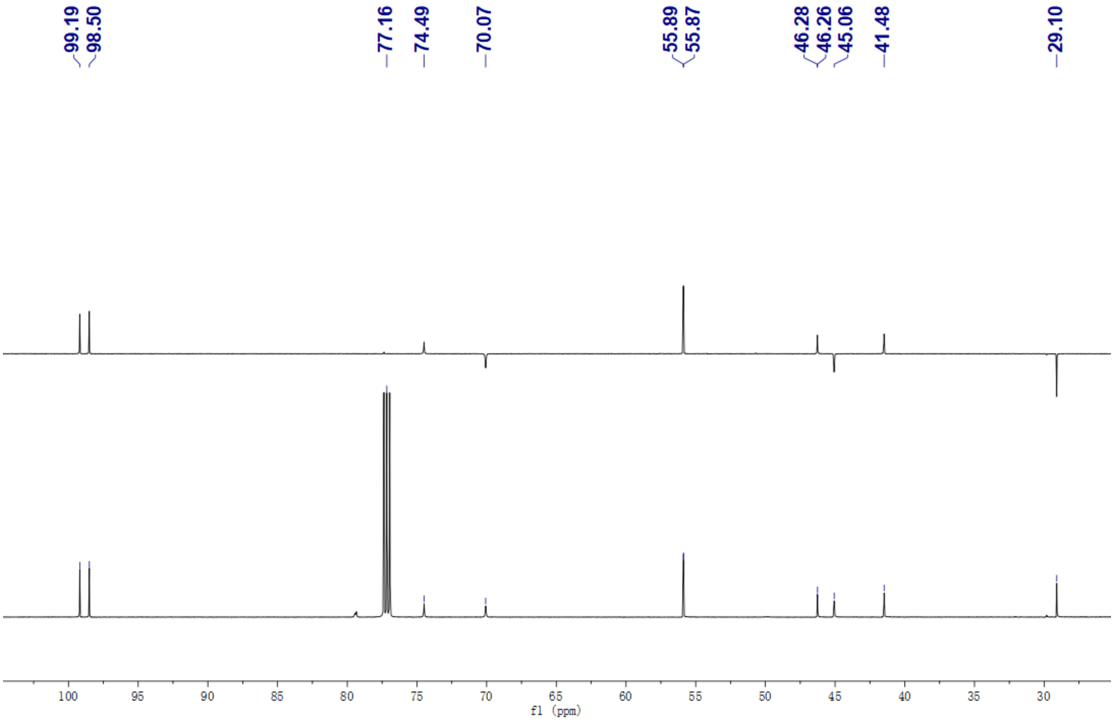

Figure S76. The ^{1}H-^{1}H COSY spectrum of **8r** ($CDCl_3$).

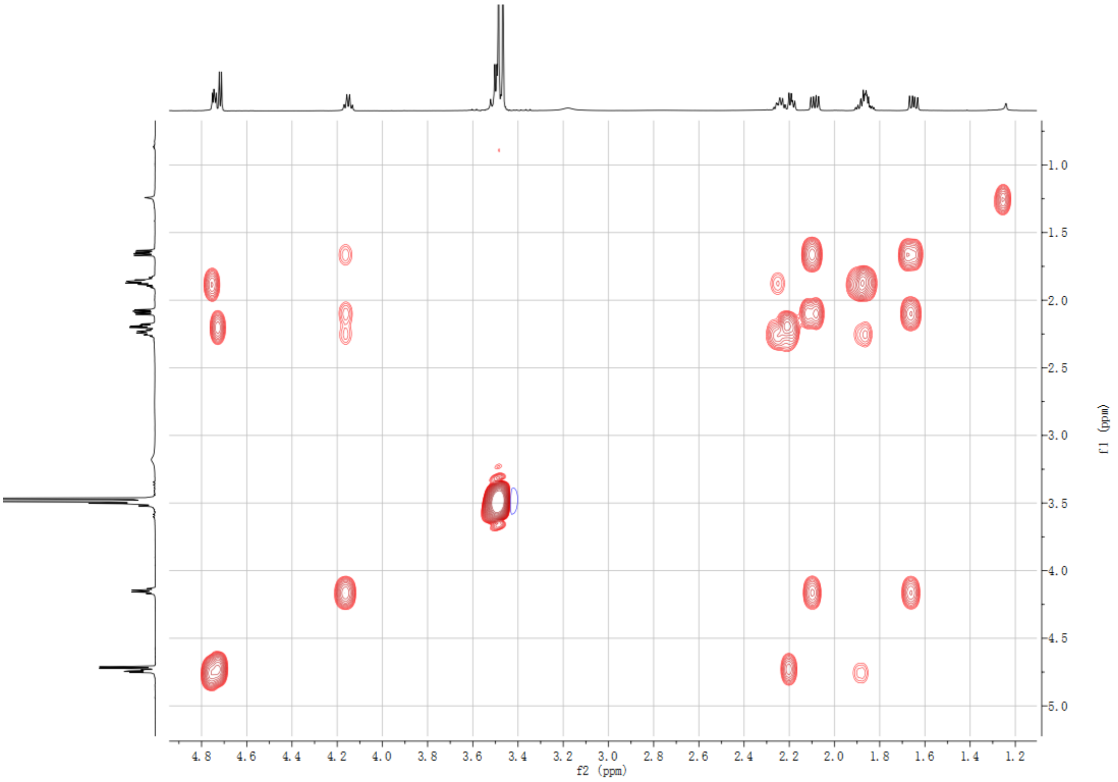

Figure S77. The NOESY spectrum of **8r** ($CDCl_3$).

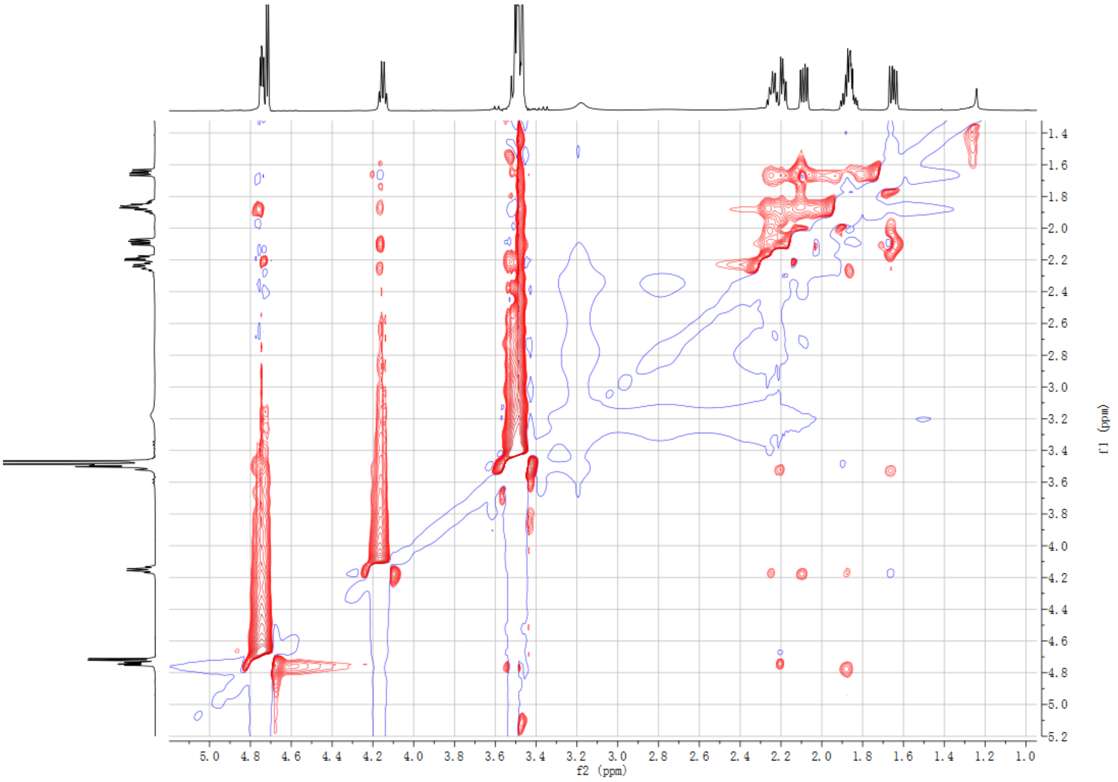

Figure S78. The ^{1}H NMR spectrum of **8r** (CD_3OD).

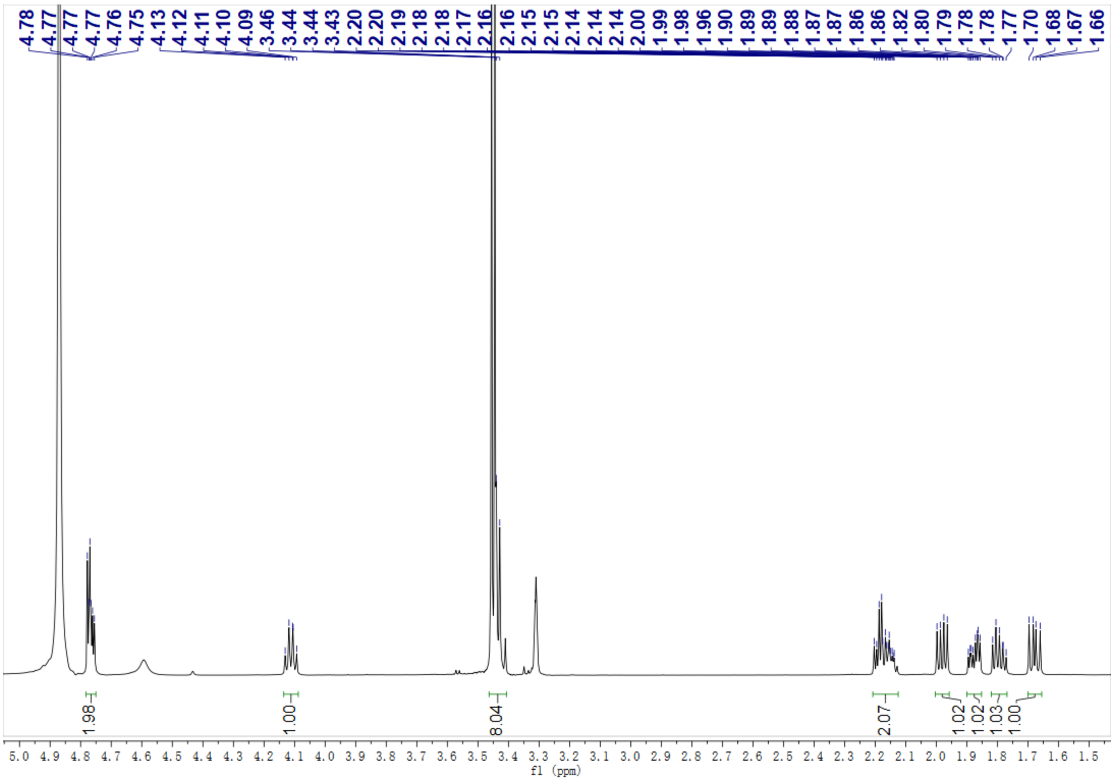

Figure S79. The ^{13}C and DEPT NMR spectra of **8r** (CD_3OD).

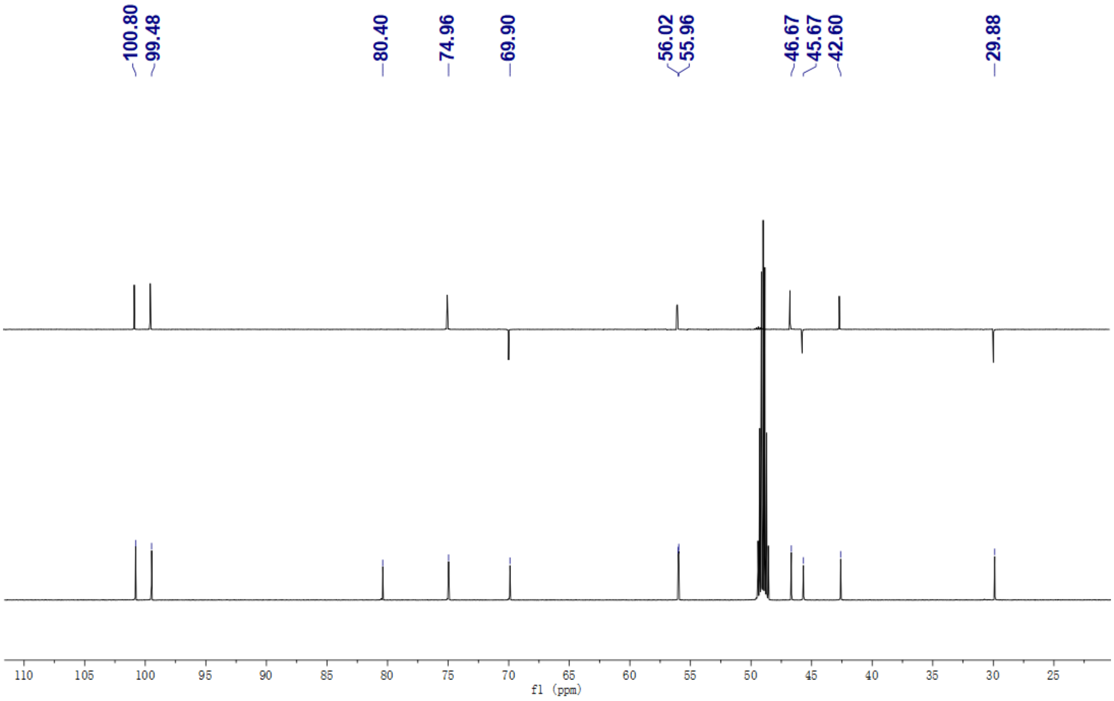

Figure S80. The ^{1}H NMR spectrum of **9** (CD_3OD).

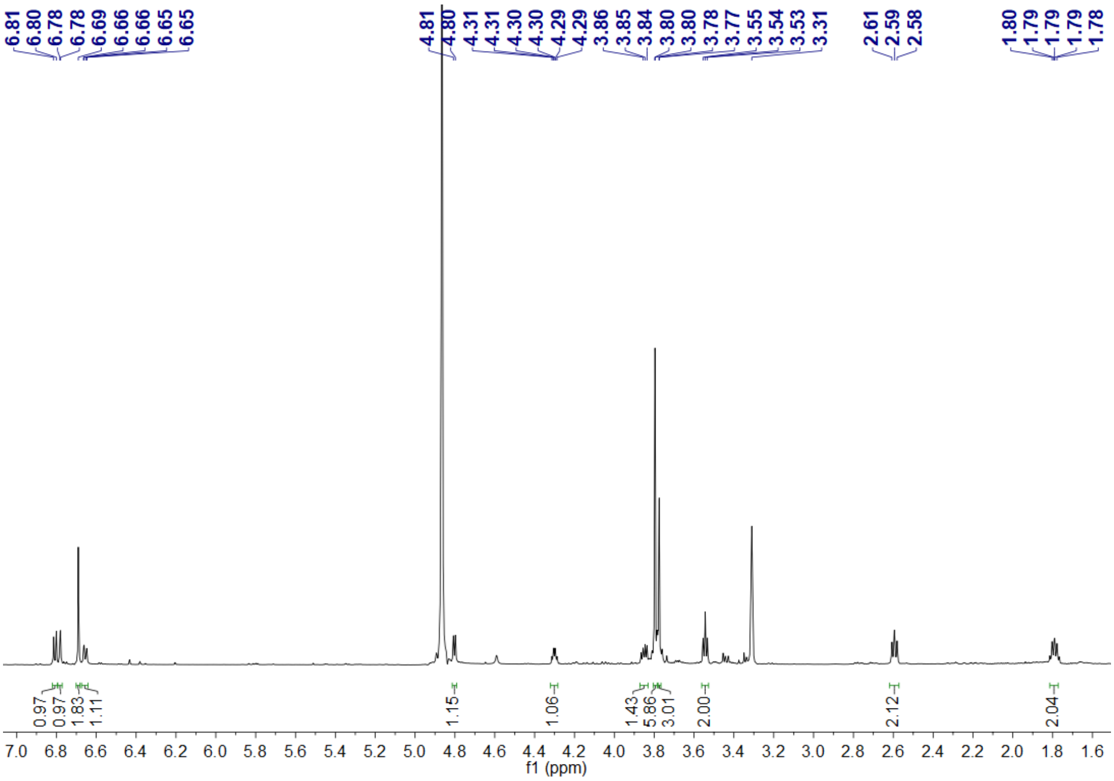

Figure S81. The ^{13}C and DEPT NMR spectra of **9** (CD_3OD).

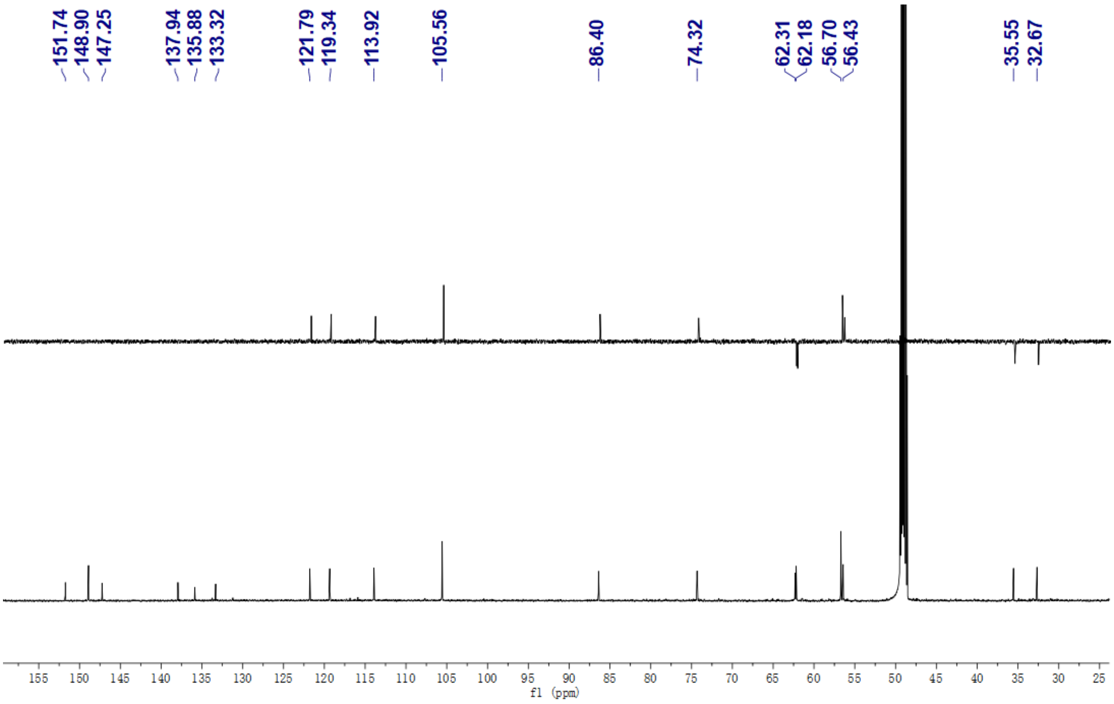

Figure S82. The ^{1}H-^{1}H COSY spectrum of **9** (CD_3OD).

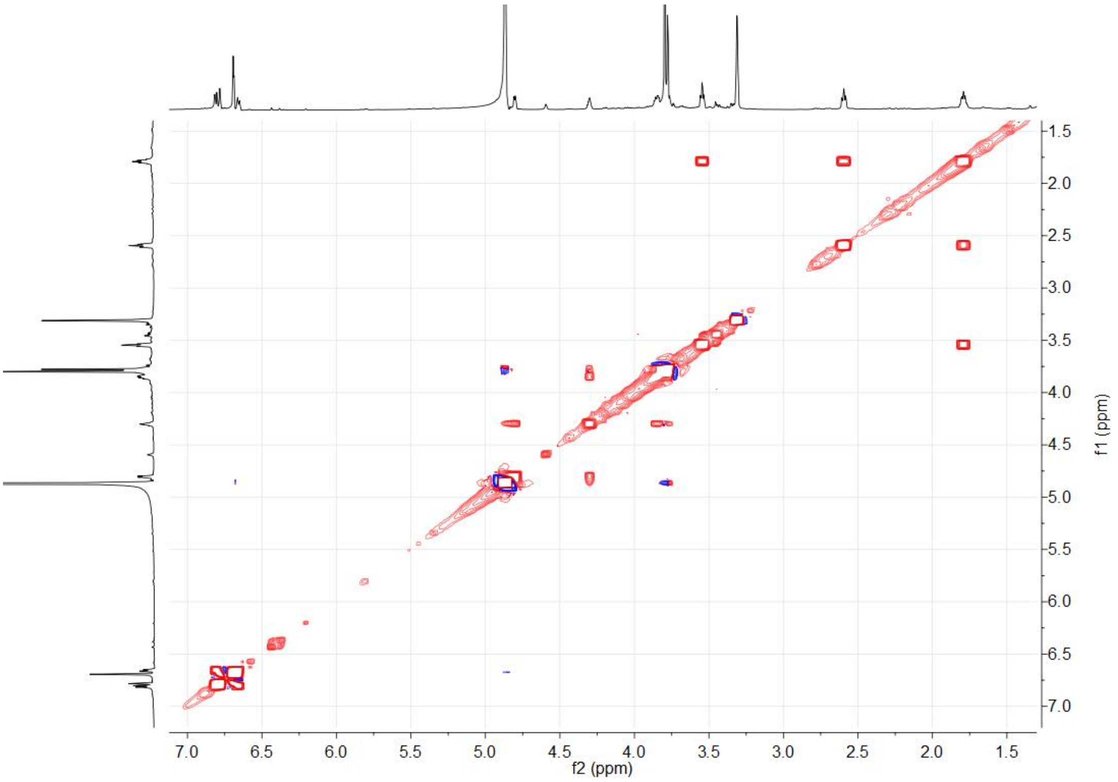

Figure S83. The HSQC spectrum of **9** (CD_3OD).

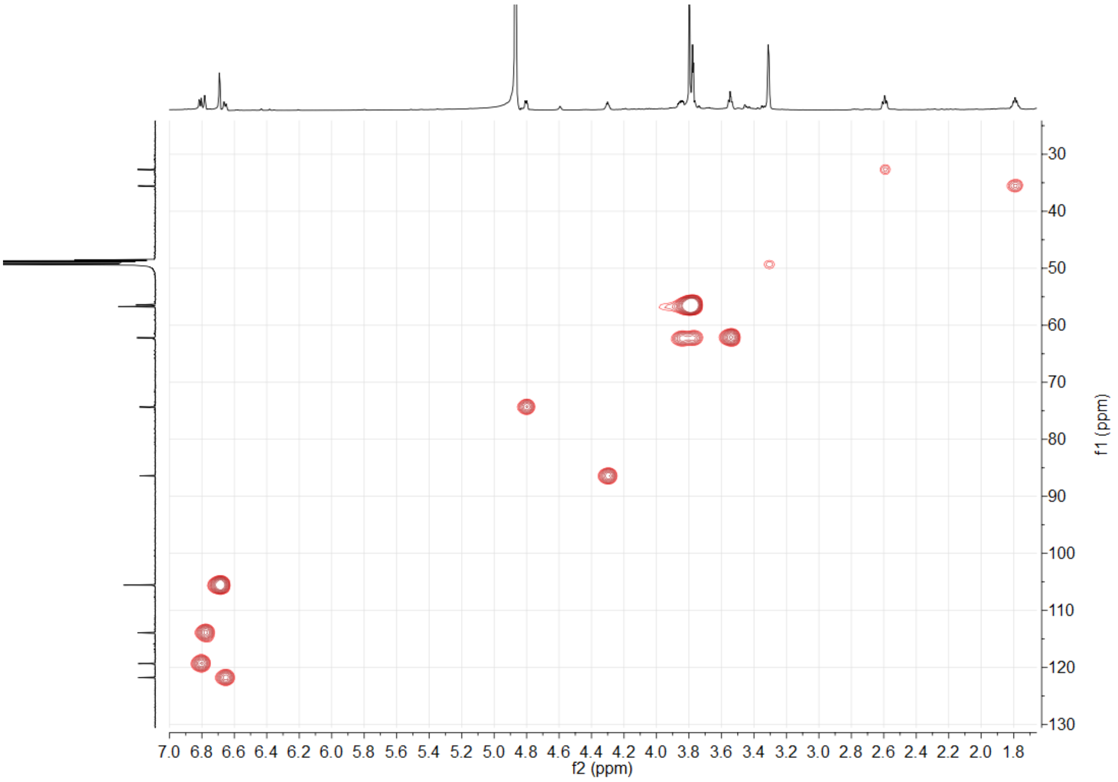

Figure S84. The HMBC spectrum of **9** (CD_3OD).

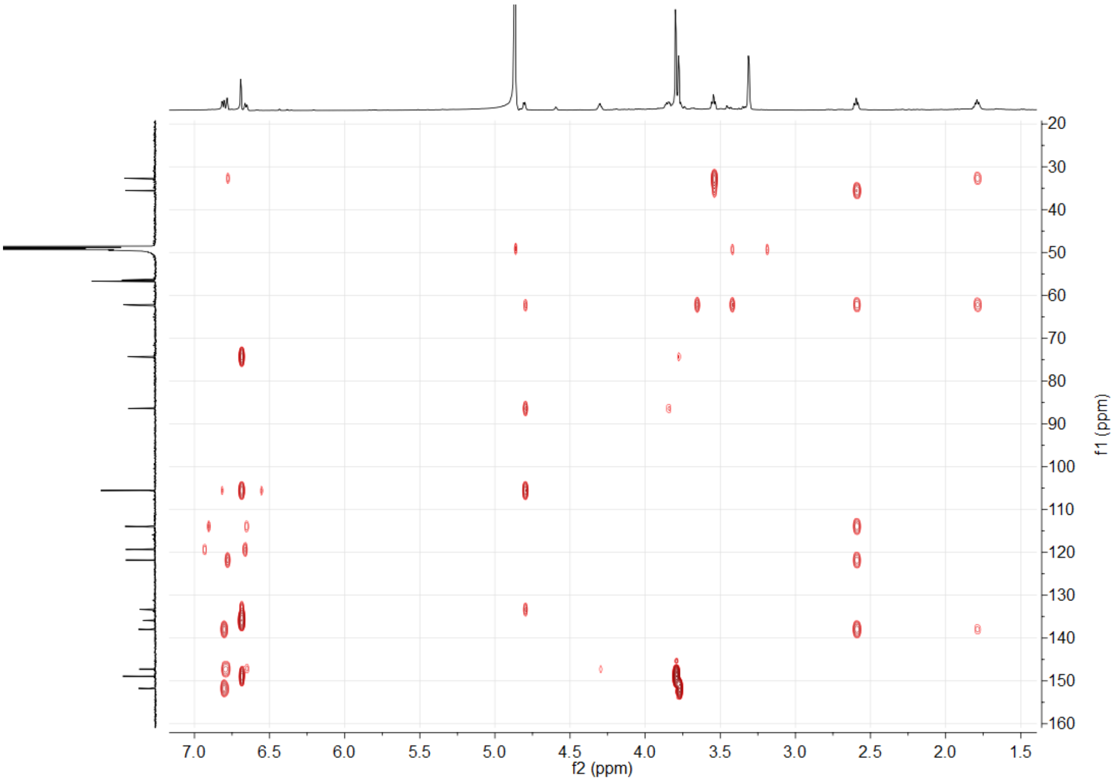

Figure S85. The NOESY spectrum of **9** (CD_3OD).

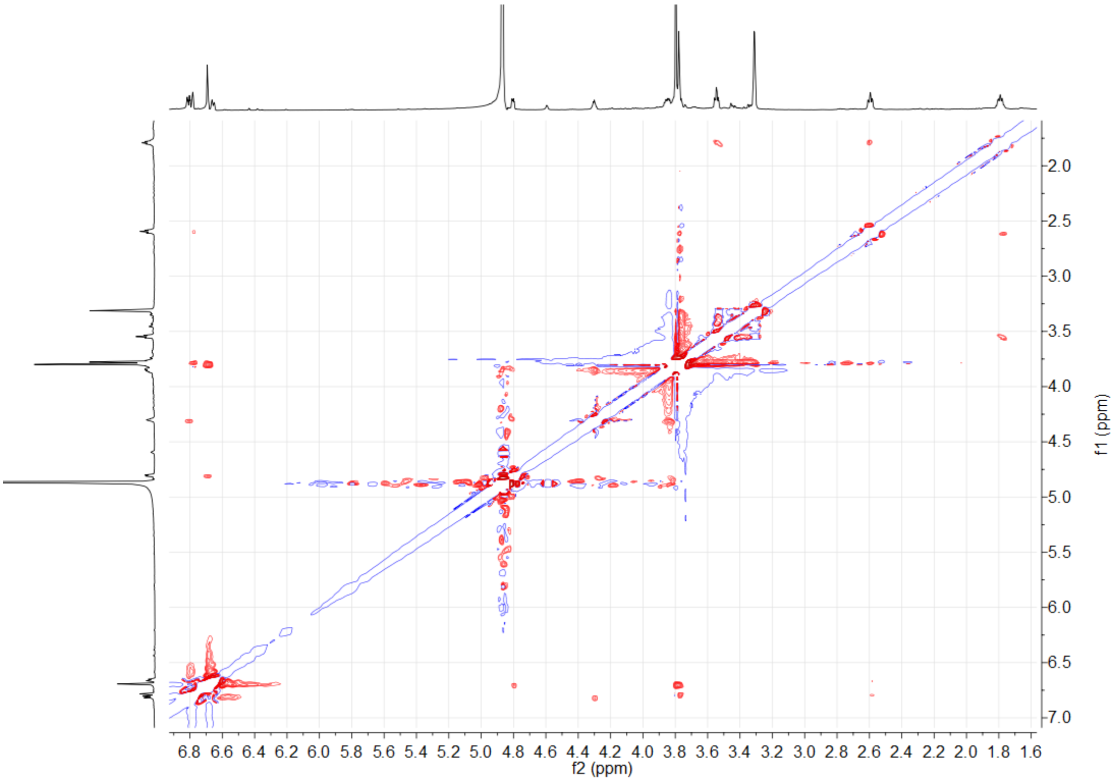

Figure S86. The (+)-HR-ESIMS spectrum of **9**.

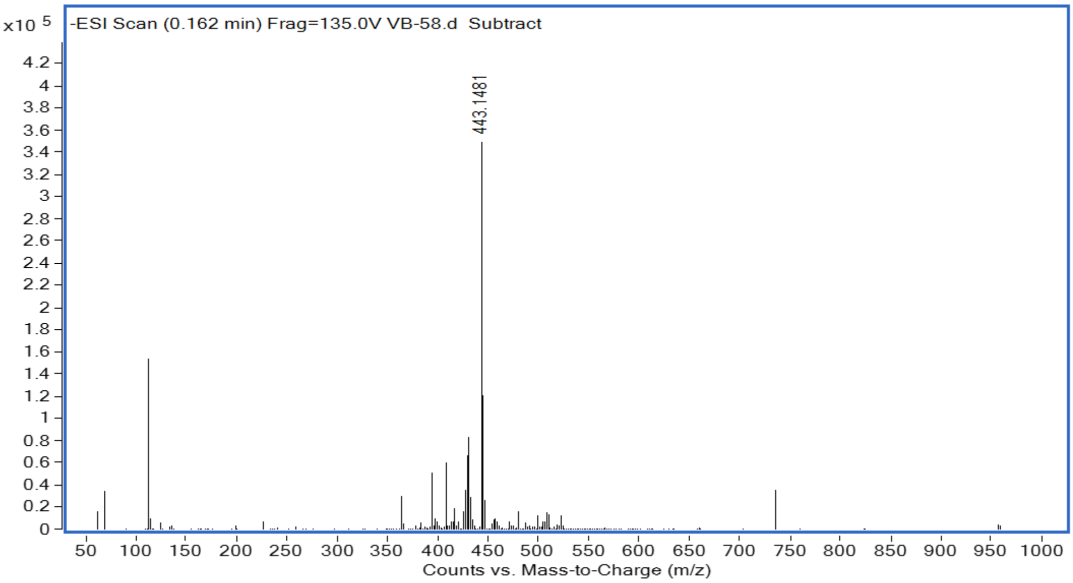

Figure S87. The ^{1}H NMR spectrum of **21** (CD_3OD).

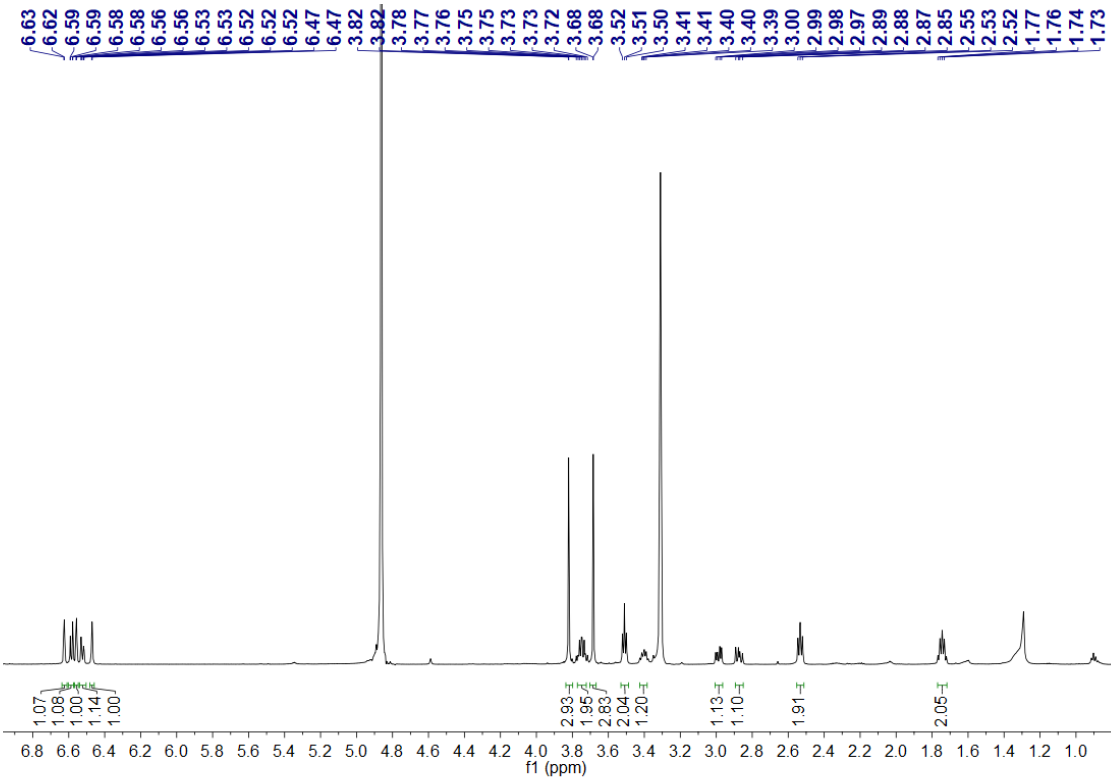

Figure S88. The ^{13}C and DEPT NMR spectra of **21** (CD_3OD).

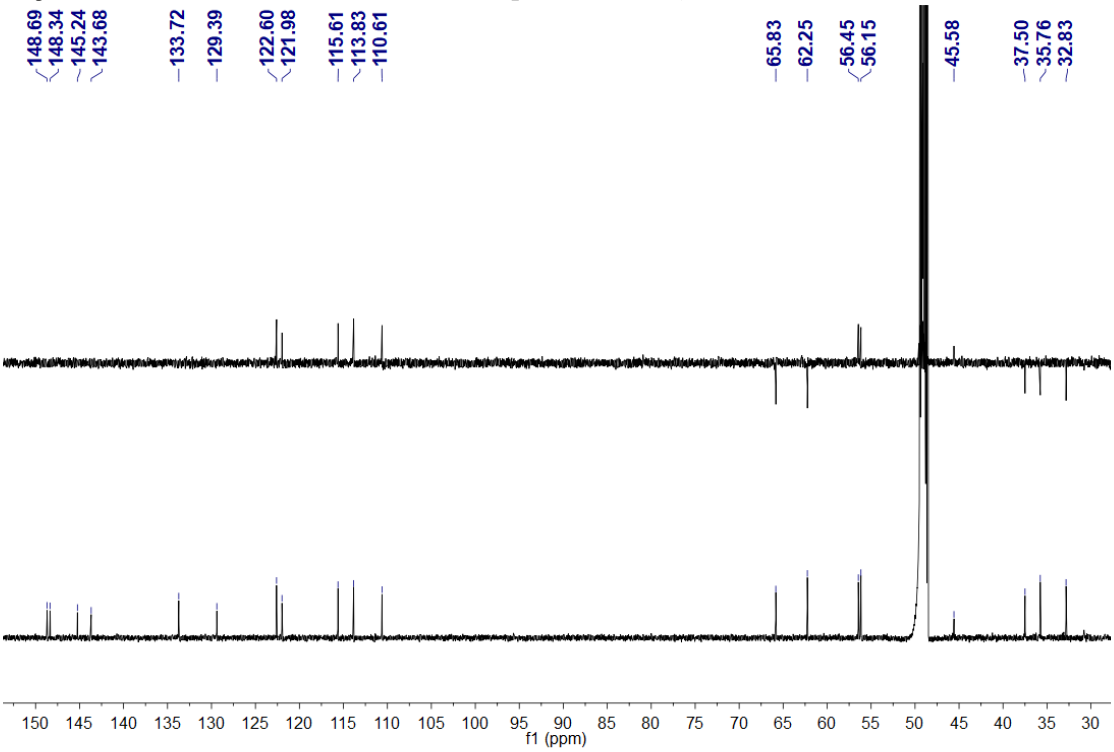

Figure S89. The ^{1}H-^{1}H COSY spectrum of **21** (CD_3OD).

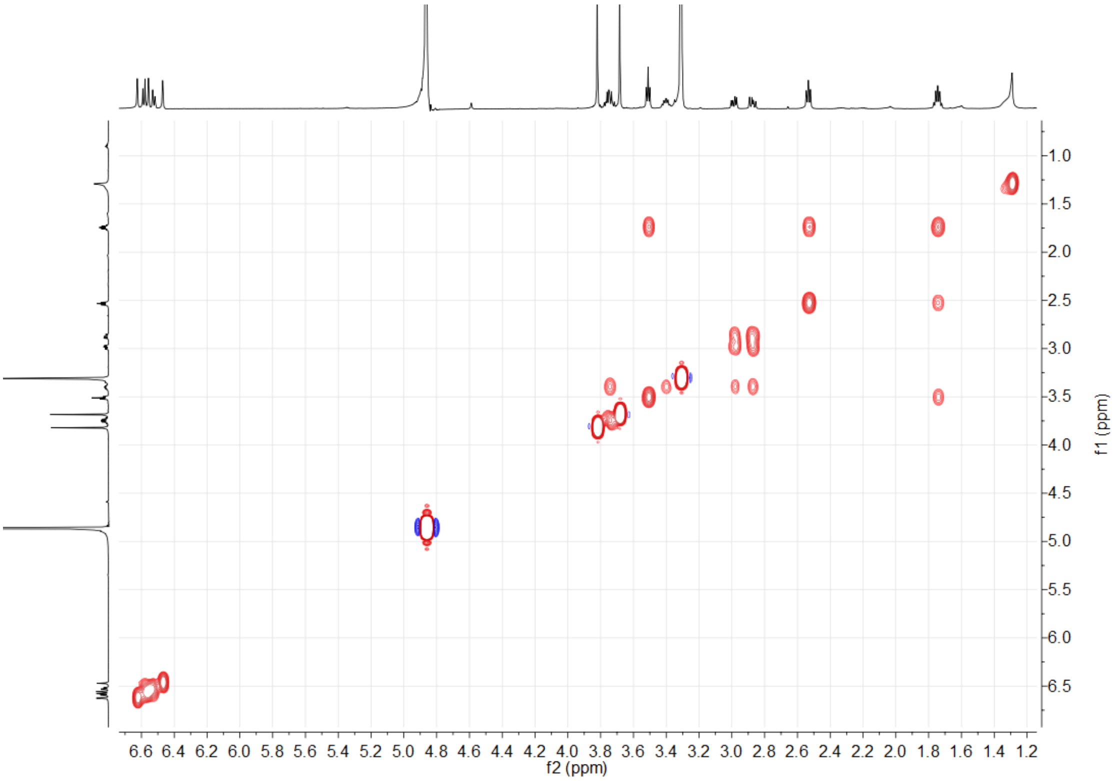

Figure S90. The HSQC spectrum of **21** (CD_3OD).

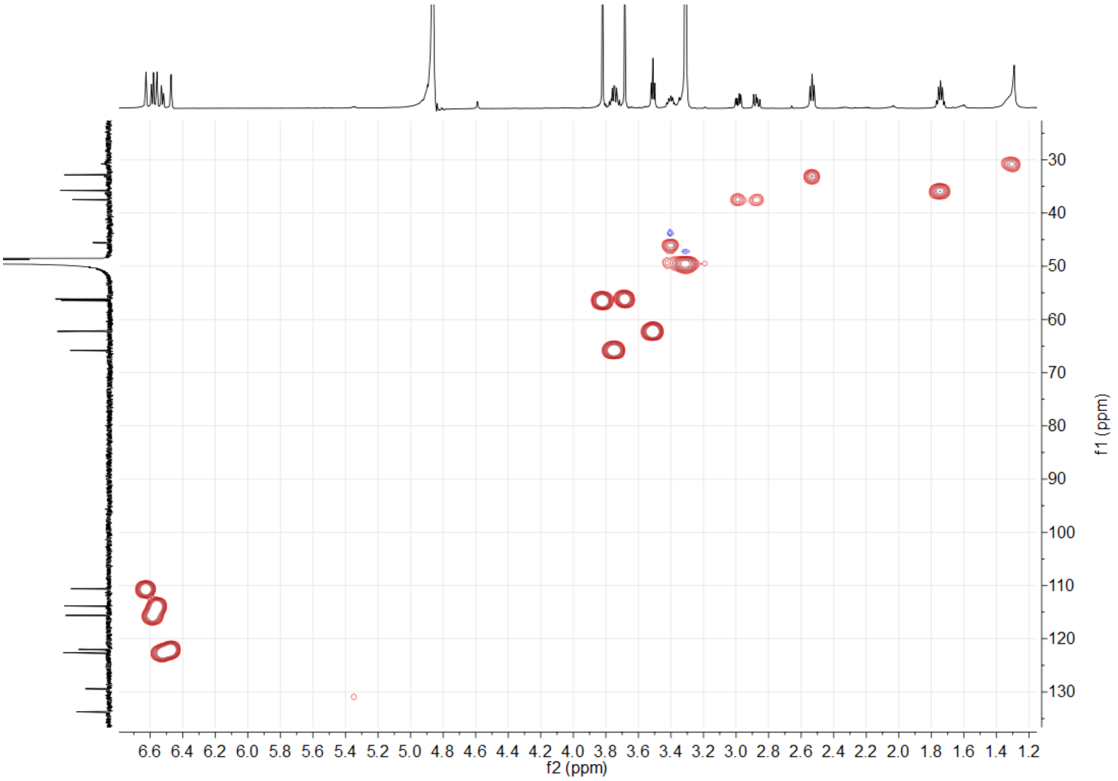

Figure S91. The HMBC spectrum of **21** (CD_3OD).

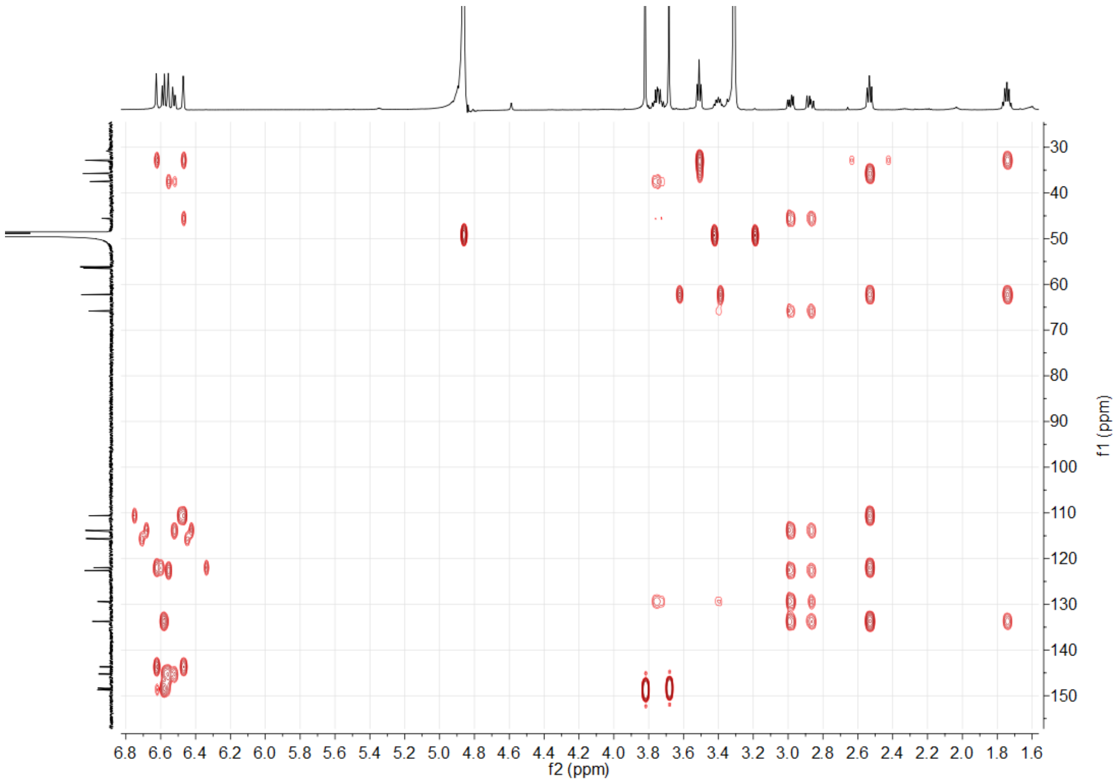

Figure S92. The NOESY spectrum of **21** (CD_3OD).

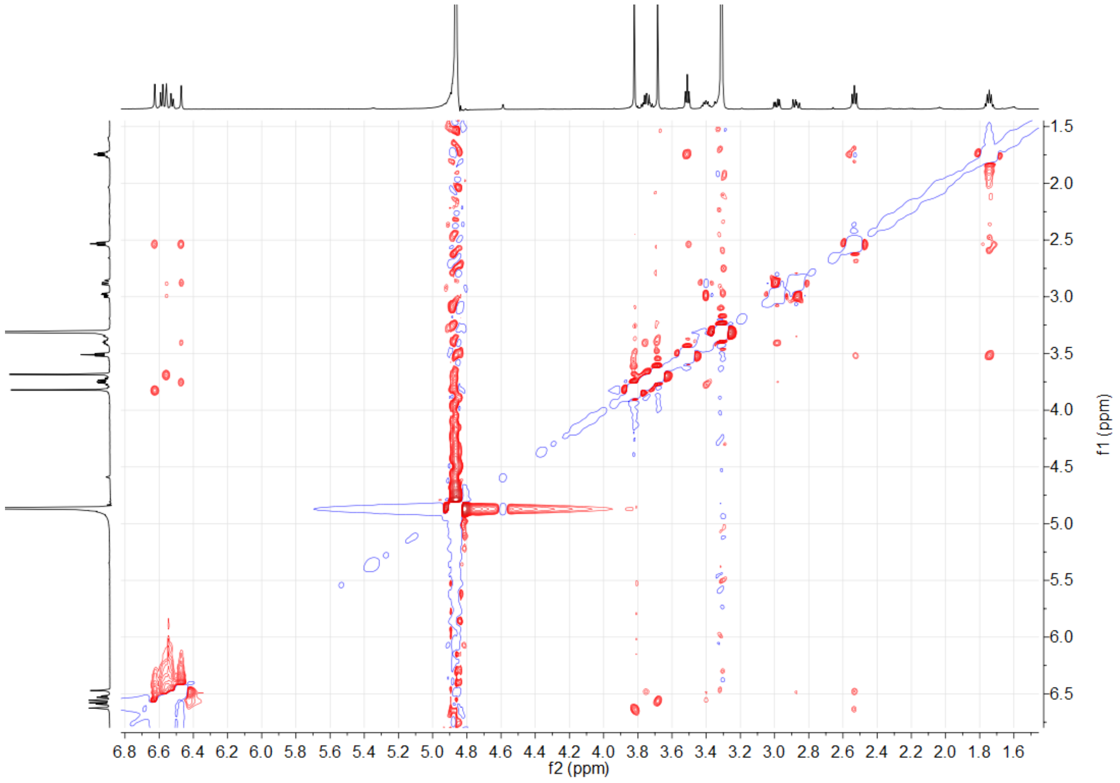

Supplement: Supplementary file 1 [file foods-12-00177-s001.zip › foods-2121512-supplementary.pdf]
